# *cis*-Oxoruthenium complexes supported by chiral tetradentate amine (N_4_) ligands for hydrocarbon oxidations†
†Electronic supplementary information (ESI) available: Experimental procedures and characterization, Scheme S1, Tables S1–S6, Fig. S1–S20. CCDC 1589975 (**1a**), CCDC 1589976 (**2a**), CCDC 1589977 (**3a**), CCDC 1589978 (**5d**), CCDC 1589979 (**6d**). For ESI and crystallographic data in CIF or other electronic format see DOI: 10.1039/c7sc05224c


**DOI:** 10.1039/c7sc05224c

**Published:** 2018-02-15

**Authors:** Chun-Wai Tse, Yungen Liu, Toby Wai-Shan Chow, Chaoqun Ma, Wing-Ping Yip, Xiao-Yong Chang, Kam-Hung Low, Jie-Sheng Huang, Chi-Ming Che

**Affiliations:** a State Key Laboratory of Synthetic Chemistry , Department of Chemistry , The University of Hong Kong , Pokfulam Road , Hong Kong , China . Email: cmche@hku.hk; b HKU Shenzhen Institute of Research and Innovation , Shenzhen , Guangdong 518053 , China; c Department of Chemistry , Southern University of Science of Technology , Shenzhen , Guangdong 518055 , China

## Abstract

We report the first examples of ruthenium complexes *cis*-[(N_4_)Ru^III^Cl_2_]^+^ and *cis*-[(N_4_)Ru^II^(OH_2_)_2_]^2+^ supported by chiral tetradentate amine ligands (N_4_), together with a high-valent *cis*-dioxo complex *cis*-[(N_4_)Ru^VI^(O)_2_]^2+^ supported by the chiral N_4_ ligand mcp (mcp = *N*,*N*′-dimethyl-*N*,*N*′-bis(pyridin-2-ylmethyl)cyclohexane-1,2-diamine). The X-ray crystal structures of *cis*-[(mcp)Ru^III^Cl_2_](ClO_4_) (**1a**), *cis*-[(Me_2_mcp)Ru^III^Cl_2_]ClO_4_ (**2a**) and *cis*-[(pdp)Ru^III^Cl_2_](ClO_4_) (**3a**) (Me_2_mcp = *N*,*N*′-dimethyl-*N*,*N*′-bis((6-methylpyridin-2-yl)methyl)cyclohexane-1,2-diamine, pdp = 1,1′-bis(pyridin-2-ylmethyl)-2,2′-bipyrrolidine)) show that the ligands coordinate to the ruthenium centre in a *cis*-α configuration. In aqueous solutions, proton-coupled electron-transfer redox couples were observed for *cis*-[(mcp)Ru^III^(O_2_CCF_3_)_2_]ClO_4_ (**1b**) and *cis*-[(pdp)Ru^III^(O_3_SCF_3_)_2_]CF_3_SO_3_ (**3c′**). Electrochemical analyses showed that the chemically/electrochemically generated *cis*-[(mcp)Ru^VI^(O)_2_]^2+^ and *cis*-[(pdp)Ru^VI^(O)_2_]^2+^ complexes are strong oxidants with *E*° = 1.11–1.13 V *vs.* SCE (at pH 1) and strong H-atom abstractors with *D*_O–H_ = 90.1–90.8 kcal mol^–1^. The reaction of **1b** or its (*R*,*R*)-mcp counterpart with excess (NH_4_)_2_[Ce^IV^(NO_3_)_6_] (CAN) in aqueous medium afforded *cis*-[(mcp)Ru^VI^(O)_2_](ClO_4_)_2_ (**1e**) or *cis*-[((*R*,*R*)-mcp)Ru^VI^(O)_2_](ClO_4_)_2_ (1e*), respectively, a strong oxidant with *E*(Ru^VI/V^) = 0.78 V (*vs.* Ag/AgNO_3_) in acetonitrile solution. Complex **1e** oxidized various hydrocarbons, including cyclohexane, in acetonitrile at room temperature, affording alcohols and/or ketones in up to 66% yield. Stoichiometric oxidations of alkenes by **1e** or 1e* in ^*t*^BuOH/H_2_O (5 : 1 v/v) afforded diols and aldehydes in combined yields of up to 98%, with moderate enantioselectivity obtained for the reaction using 1e*. The *cis*-[(pdp)Ru^II^(OH_2_)_2_]^2+^ (**3c**)-catalysed oxidation of saturated C–H bonds, including those of ethane and propane, with CAN as terminal oxidant was also demonstrated.

## Abstract 2

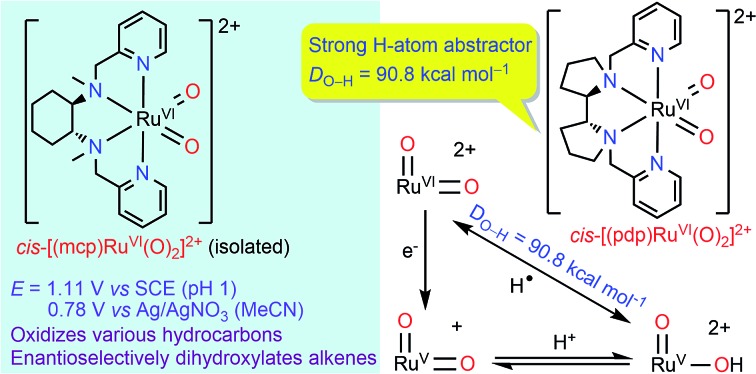
We report the first examples of *cis*-dioxo ruthenium(vi) complexes supported by chiral N_4_ ligands and their stoichiometric and catalytic reactivities with alkanes and alkenes.

## Introduction

**Fig. 1:**
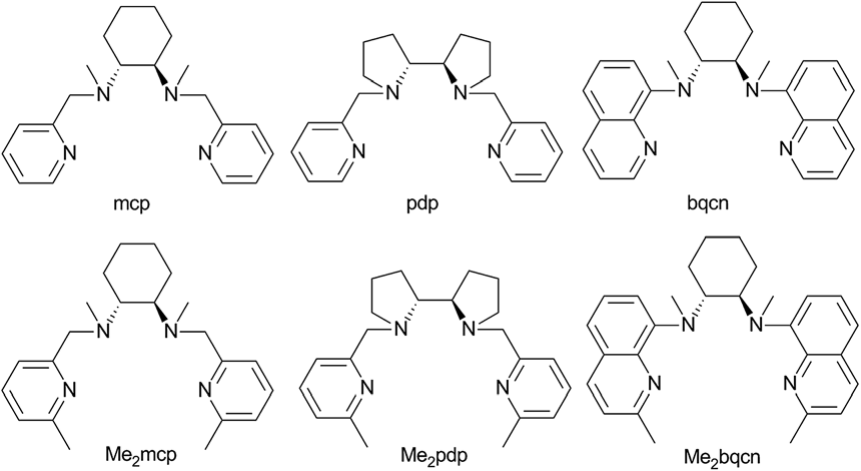
Structures and abbreviations of chiral N_4_ ligands used in this study. Ligands were prepared as either the (*R*,*R*)-enantiomer or a racemic mixture.

**Fig. 2:**
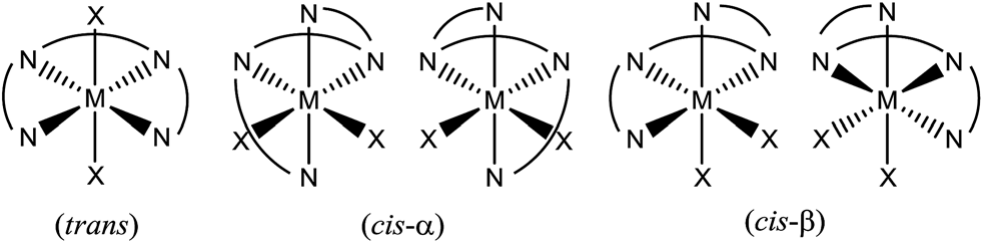
Different wrapping modes of acyclic tetradentate N_4_ ligands in an octahedral environment.

The selective oxidations of hydrocarbons^1^ including alkanes and alkenes, and oxidation of alcohols,^2^ catalysed by metal complexes under mild conditions are important reactions in chemical synthesis. Iron and manganese complexes bearing tetradentate pyridylmethyl amine or quinolylamine N_4_ ligands1b,h,i,k–o,r constitute one of the platforms for performing efficient and selective C–H and C=C functionalizations, wherein the widely employed N_4_ ligands include mcp, pdp and bqcn ligands and their derivatives (examples depicted in Fig. 1).^3^–^6^ These acyclic chiral tetradentate amine (N_4_) ligands, in most scenarios, coordinate to metal ions in a *cis*-α configuration to form octahedral metal complexes (Fig. 2), leaving a pair of *cis* sites for oxidant activation or substrate binding. Stereoretentive C–H hydroxylation,3a,b,f,4a,b enantioselective epoxidation5c–f,6a,c–e and asymmetric *cis*-dihydroxylation (AD) of alkenes5g,6b have been achieved under limiting substrate conditions. One type of proposed active metal–oxo intermediates in these oxidation reactions catalysed by metal chiral N_4_ complexes is the corresponding high-valent *cis*-dioxo complexes, *i.e.*, *cis*-M(O)_2_ species supported by chiral N_4_ ligands.6*b* In the iron-catalysed systems, *cis*-[(N_4_)Fe^V^(O)(OR)]^2+^ (R = H or acyl) active intermediates,5h,^7^,^8^,^9^ and a *cis*-[(N_4_)Fe^V^(O)_2_]^+^ active intermediate in alkene *cis*-dihydroxylation,^10^ have been proposed; isolation of these active species and elucidation of the reaction mechanisms in these iron systems have often been difficult because of the extraordinary reactivity of high-valent iron–oxo complexes. A proposed *cis*-[(N_4_)Mn^V^(O)_2_]^+^ intermediate, *cis*-[((*S*,*S*)-bqcn)Mn^V^(O)_2_]^+^,6*b* in manganese-catalysed enantioselective *cis*-dihydroxylation of alkenes was also found to be insufficiently stable for isolation. While several *cis*-[(N_4_)Re^V^(O)_2_]^+^ complexes and a *cis*-[(N_4_)Re^VI^(O)_2_]^2+^ complex have been isolated and structurally characterized in our recent work,^11^ the former were not reactive towards organic substrates, and concerning hydrocarbon oxidation reactivity, the latter only reacted with weak C–H bonds (bond dissociation energy: ∼76 kcal mol^–1^) of 1,4-cyclohexadiene, 9-10-dihydroanthracene and xanthene at 80 °C to give dehydrogenation or ketone products.

To search for isolable *cis*-dioxo metal complexes that are supported by the abovementioned chiral N_4_ ligands and are reactive towards hydrocarbon oxidation, including the *cis*-dihydroxylation of alkenes and the oxidation of strong C–H bonds at room temperature, we directed our efforts to ruthenium systems. High-valent Ru–oxo complexes are generally more stable than their iron counterparts due to their lower redox potentials as well as substitutional inertness of auxiliary ligands.^12^*cis*-Dioxoruthenium(vi) complexes can have a delicate balance between stability and reactivity that allows them to be isolated/characterized^12^,^13^ or even studied in reactions with organic substrates in a stoichiometric manner.^14^–^16^ Several cationic *cis*-dioxoruthenium(vi) complexes, including *cis*-[(Tet-Me_6_)Ru^VI^(O)_2_]^2+^ (Tet-Me_6_ = *N*,*N*,*N*′,*N*′-3,6-hexamethyl-3,6-diazooctane-1,8-diamine),14*a**cis*-[(Me_3_tacn)Ru^VI^(O)_2_(O_2_CCF_3_)]^+^ (Me_3_tacn = 1,4,7-trimethyl-triazacyclononane),15*a**cis*-[(6,6′-Cl_2_bpy)_2_Ru^VI^(O)_2_]^2+^ (6,6′-Cl_2_bpy = 6,6′-dichloro-2,2′-bipyridine),16*a**cis*-[(bpy)_2_Ru^VI^(O)_2_]^2+^ (bpy = 2,2′-bipyridine)^17^ and *cis*-[(dmp)_2_Ru^VI^(O)_2_]^2+^ (dmp = 2,9-dimethyl-1,10-phenanthroline),^18^ have been isolated and/or spectroscopically characterized. Among them, *cis*-[(Me_3_tacn)Ru^VI^(O)_2_(O_2_CCF_3_)]^+^ and *cis*-[(6,6′-Cl_2_bpy)_2_Ru^VI^(O)_2_]^2+^ are known to react with simple saturated alkanes (*e.g.*, cyclohexane) stoichiometrically.^15^,^16^ The related catalytic oxygenation of cyclohexane with ^*t*^BuOOH could be performed with [(Me_3_tacn)Ru^III^Cl_3_]^19^ and *cis*-[(Cl_2_bpy)_2_Ru^II^(OH_2_)_2_]^2+^ as catalysts.16*b* Du Bois and co-workers recently demonstrated selective C–H functionalization catalysed by [(Me_3_tacn)Ru^III^Cl_3_] with ceric ammonium nitrate (CAN) as a terminal oxidant to give tertiary C–H hydroxylation products.^20^ Using bis(bipyridine)Ru catalysts, the selective functionalization of amine derivatives was attainable with various oxidants and acid additives.^21^ These studies highlight the amendable oxidation capabilities of the *cis*-dioxoruthenium(vi) moiety and the underdeveloped potential of ruthenium catalysts in C–H oxidation.

Thus far, studies on highly oxidizing *cis*-dioxoruthenium(vi) complexes have focused on tridentate Me_3_tacn and simple bidentate aromatic diimine ligands.^15^–^18^ The Me_3_tacn ligand is not flexible for structure modification.^21^ Ruthenium complexes with aromatic diamine ligands may undergo *cis*–*trans* isomerization^22^ and ligand loss in a high oxidation state.^17^ These difficulties can potentially be resolved by utilizing the abovementioned chiral N_4_ ligands: the first coordination sphere is highly tuneable by ligand modification, as revealed by recent works from White,3*d* Costas,3b,4c,d and their co-workers; the higher rigidity and denticity can provide better conformational stability under catalytic conditions.

In this work, we aim to (i) isolate/generate *cis*-dioxoruthenium(vi) complexes bearing chiral tetradentate amine (N_4_) ligands, (ii) study the redox potentials and hydrocarbon oxidation reactions of these *cis*-[(N_4_)Ru^VI^(O)_2_]^2+^ complexes, and (iii) gain insight into the activity of chiral Ru(N_4_) complex in asymmetric oxidation reactions. Until now, studies on ruthenium complexes supported by chiral N_4_ ligands (mcp, pdp, bqcn and their derivatives) have been limited,^23^–^25^ including a report involving some data of [Ru^II^(mcp)Cl_2_]-catalysed oxidation of thioanisole with H_2_O_2_,25*a* another report involving the synthesis and crystallographic characterization of [Ru^II^((*R*,*R*)-pdp)(NCMe)_2_]^2+^,25*b* and density functional calculations on a hypothetical monooxo ruthenium(iv) species *cis*-[(bqcn)Ru^IV^(O)(NCMe)]^2+^.^26^ No examples of the corresponding *cis*-dioxo ruthenium chiral N_4_ complexes have been reported.^23^–^26^ Herein, we describe the syntheses, characterization, and electrochemical and reactivity studies of a series of chiral Ru(N_4_) complexes, including a highly reactive chiral *cis*-[(N_4_)Ru^VI^(O)_2_]^2+^ complex that can perform dihydroxylation of alkenes and oxidation of strong C–H bonds of alkanes (including cyclohexane) and oxidation of alcohols at room temperature. The studies on *cis*-[(N_4_)Ru^VI^(O)_2_]^2+^ complexes provide insight into the reactivity and electrochemical properties of the analogous highly oxidizing *cis*-[(N_4_)M(O)_2_]^*n*+^ (*n* = 1 or 2; M = Fe or Mn) species.6b,^10^

## Results

### Synthesis and characterization

**Scheme 1:**
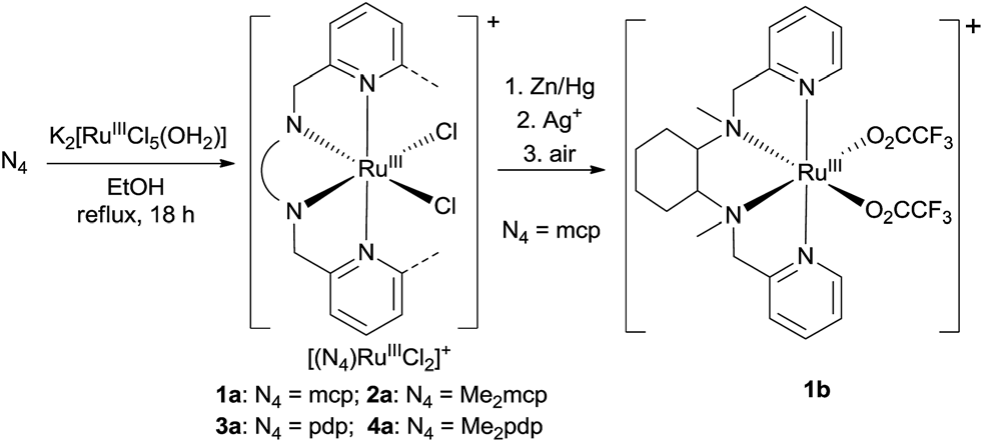
Preparation of **1a–4a** and **1b**.

**Scheme 2:**
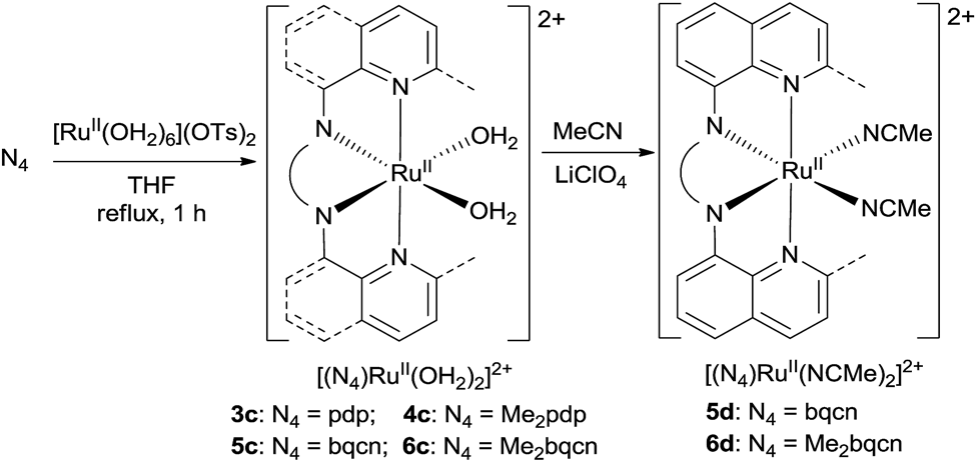
Preparation of **3c–6c**, **5d** and **6d**.

In this work, a series of ruthenium complexes bearing six chiral tetradentate amine N_4_ ligands (Fig. 1) and different auxiliary ligands were prepared (Schemes 1 and 2). The reaction of K_2_[Ru^III^Cl_5_(OH_2_)] with the mcp, Me_2_mcp, pdp or Me_2_pdp ligand in ethanol under refluxing conditions (Scheme 1) gave the corresponding *cis*-[(N_4_)Ru^III^Cl_2_]^+^ complex (**1a**, **2a**, **3a** or **4a**) in 32–97% yield.^27^ The reaction of **1a** with Zn/Hg in distilled water at 80 °C for 30 min, followed by subsequent treatment of the solution with AgOTf and 0.2 M CF_3_CO_2_H, afforded *cis*-[(mcp)Ru^III^(O_2_CCF_3_)_2_]ClO_4_ (**1b**) in 20% yield (Scheme 1).^28^ To prepare ruthenium complexes containing the bqcn and Me_2_bqcn ligands, an alternative synthetic method was developed. Treatment of bqcn or Me_2_bqcn with a slight excess (1.2 equiv.) of [Ru^II^(OH_2_)_6_](OTs)_2_ under Ar in THF furnished the OTs^–^ salt of *cis*-[(N_4_)Ru^II^(OH_2_)_2_]^2+^ (**5c** or **6c**) in good yield (up to 71%). A similar treatment using pdp or Me_2_pdp gave the OTs^–^ salt of **3c** or **4c**. Recrystallization of **5c·OTs** or **6c·OTs** in acetonitrile in the presence of LiClO_4_ produced *cis*-[(bqcn)Ru^II^(NCMe)_2_](ClO_4_)_2_ (**5d**) and *cis*-[(Me_2_bqcn)Ru^II^(NCMe)_2_](ClO_4_)_2_ (**6d**), respectively (Scheme 2).

**Fig. 3:**
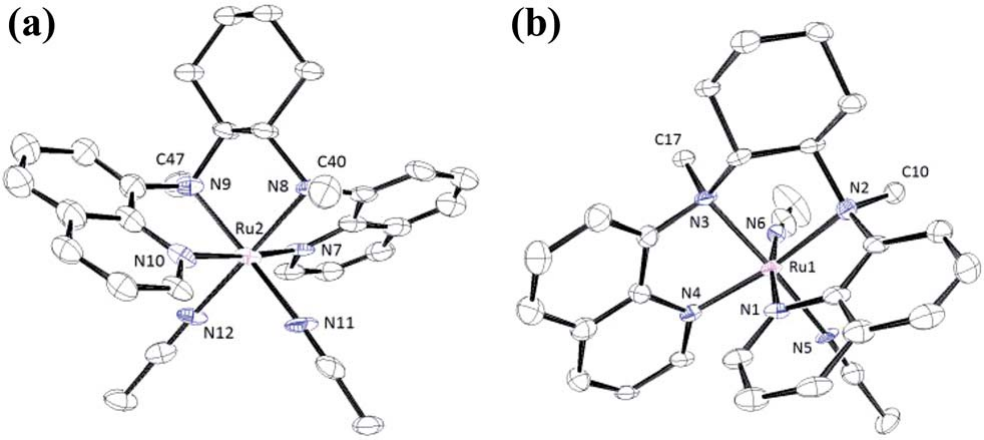
ORTEP drawings of the complex cations of *cis*-[(bqcn)Ru^II^(NCMe)_2_](ClO_4_)_2_ (**5d**). (a, left) *cis*-α isomer (α-**5d**). (b, right) *cis*-β isomer (β-**5d**). Hydrogen atoms are omitted for clarity. Thermal ellipsoids are drawn at the 30% probability level.

The structures of **1a**, **2a**, **3a**, **5d** and **6d** (as ClO_4_^–^ salts) were established by X-ray crystallography. All these complexes, except **5d**, adopt a *cis*-α configuration (Fig. 3 and S1–S5, ESI†), where the two terminal pyridyl/quinolyl groups are positioned *trans* to each other. For **5d**, its crystal structure showed that the *cis*-α and *cis*-β isomers are present in a 1 : 1 ratio in the unit cell.^29^ The two isomers could not be separated by repeated recrystallizations. Fig. 3a depicts the structure of *cis*-α-**5d**; its two methyl groups on the cyclohexane-1,2-diamine nitrogen atoms are oriented *anti* to each other (C40 and C47). In the *cis*-β isomer (Fig. 3b), the corresponding two methyl groups (C10 and C17) show the opposite (*syn*) orientation.

The ^1^H NMR spectra of **3c**, **4c** (in CD_3_CN) and **6d** show signals indicative of a *cis*-α configuration (Fig. 2) with *C*_2_ symmetry (see Experimental section); no interconversion to the *cis*-β conformer was observed by standing the solution at room temperature for days. In contrast, the ^1^H NMR spectrum of **5d** comprises a mixture of signals from the *cis*-α and *cis*-β isomers.

The UV-Vis absorption spectra of the *cis*-[(N_4_)Ru^III^Cl_2_]^+^ complexes (**1a**, **2a**, **3a** and **4a**) in acetonitrile solution are characterized by *p*_π_(Cl) → Ru(iii) LMCT transition band at *λ* 400–450 nm (*ε* = 900–2000 dm^3^ mol^–1^ cm^–1^, Fig. S6, ESI†).^27^ In aqueous solutions, the *cis*-[(N_4_)Ru^II^(OH_2_)_2_]^2+^ complexes (**3c**, **4c**, **5c** and **6c**) show intense absorption bands at 361–477 nm (*ε* = 4700–6900 dm^3^ mol^–1^ cm^–1^, Fig. S7, ESI†) assignable to *d*_π_ (Ru) → *p*_π*_ (pyridyl or quinolyl) MLCT transitions.

The *cis*-dichlororuthenium(iii) complexes display different cyclic voltammetric behaviours in acetonitrile solutions (Fig. S9, ESI†). Complexes **1a** and **3a** display a reversible couple at *E*_1/2_ = *ca.* 0 V *vs.* SCE. This is assigned to the Ru^III/II^ couple: *cis*-[(N_4_)Ru^III^Cl_2_]^+^ + e^–^ → *cis*-[(N_4_)Ru^II^Cl_2_]^0^. The Ru^IV/III^ couple was not observed at potentials up to 1.6 V *vs.* SCE. For **2a**, where the N_4_ ligand possesses a methyl substituent on the pyridyl moiety, the Ru^III/II^ couple is irreversible. The irreversible reduction of *cis*-[(N_4_)Ru^III^Cl_2_]^+^ occurs at *E*_pc_ = –0.01 V; upon the reverse scan, an oxidation wave appears at *E*_pa_ = 0.69 V, which is attributed to the oxidation of *cis*-[(Me_2_mcp)Ru^II^Cl(NCMe)]^+^ to *cis*-[(Me_2_mcp)Ru^III^Cl(NCMe)]^2+^ after a ligand exchange reaction of [(Me_2_mcp)Ru^II^Cl_2_]^0^ with the solvent.^30^,^31^ The cyclic voltammograms of **5d** and **6d** in MeCN display reversible oxidation couples at *E*_1/2_ = 1.35 V and 1.36 V *vs.* SCE, respectively (Fig. S11, ESI†). The electrochemical reaction is assigned to: *cis*-[(N_4_)Ru^III^(NCMe)_2_]^3+^ + e^–^ → *cis*-[(N_4_)Ru^II^(NCMe)_2_]^2+^.

### Aqueous electrochemistry of *cis*-[(mcp)Ru^III^(O_2_CCF_3_)_2_]ClO_4_ (**1b**) and *cis*-[(pdp)Ru^III^(O_3_SCF_3_)_2_]CF_3_SO_3_ (**3c′**)

**Fig. 4:**
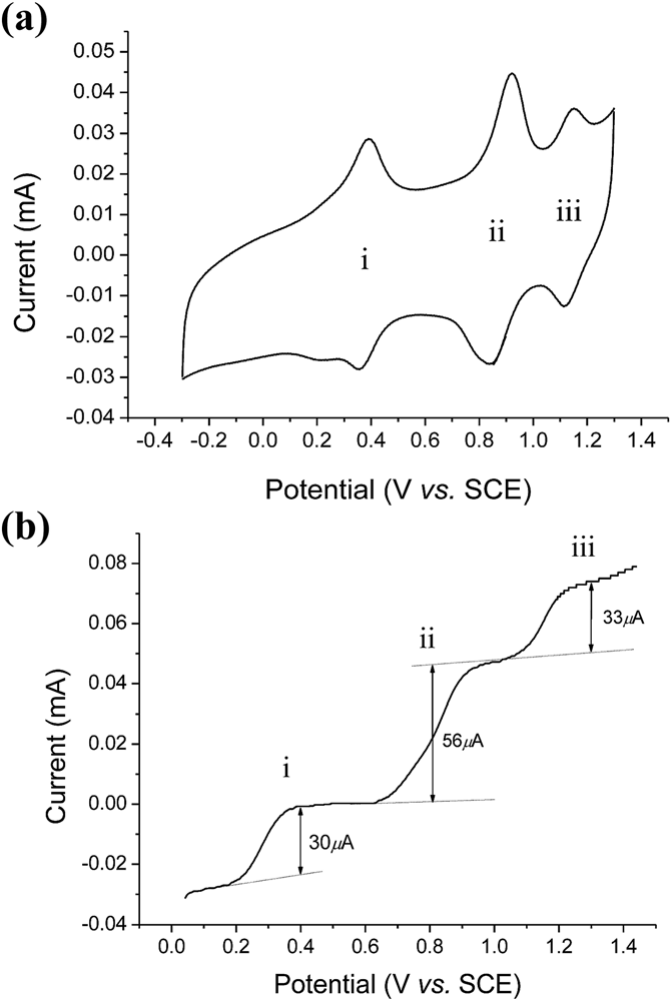
(a, upper) Cyclic voltammogram of *cis*-[(mcp)Ru^III^(O_2_CCF_3_)_2_]ClO_4_ (**1b**) at pH 1. (b, lower) Rotating-disk electrode voltammogram of *cis*-[(mcp)Ru^III^(O_2_CCF_3_)_2_]ClO_4_ (**1b**) at pH 1. Working electrode: edge-plane pyrolytic graphite for CV; glassy carbon for RDEV. Rest potentials: *ca.* 0.55 V.

**Scheme 3:**
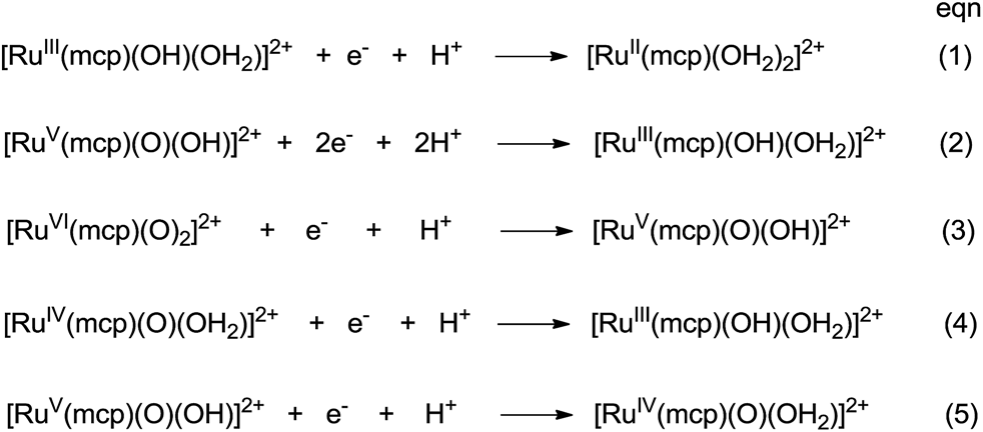
Proposed redox couples for *cis*-[(mcp)Ru^III^(O_2_CCF_3_)_2_]ClO_4_ (**1b**) in different pH buffer solutions. The *cis*-sign is omitted for clarity.

The cyclic voltammogram of *cis*-[(mcp)Ru^III^(O_2_CCF_3_)_2_]ClO_4_ (**1b**) at pH 1 displays three reversible/quasi-reversible couples (i), (ii) and (iii) at *E*_1/2_ = 0.37, 0.92 and 1.11 V *vs.* SCE, respectively (Fig. 4a). Using rotating-disk electrode voltammetry, the coulombic stoichiometries of the redox couples were determined to be 1.0, 1.9 and 1.1 for couples (i), (ii) and (iii), respectively (Fig. 4b). With reference to previous work,^27^ these couples could be assigned to Ru^III/II^, Ru^V/III^ and Ru^VI/V^ redox processes, and the electrochemical reactions (1)–(3) are depicted in Scheme 3. At pH 5, the *E*_1/2_ of couples (i) and (iii) shift to 0.25 and 0.98 V, respectively, and couple (ii) splits into two reversible one-electron couples (iv) and v at 0.65 and 0.77 V, respectively (Fig. S12, ESI†). Couples (iv) and (v) are assigned to Ru^IV/III^ and Ru^V/IV^ couples (eqn (4) and (5) in Scheme 3). The cathodic shift in the *E*_1/2_ of couple (iii) with an increasing pH is in accordance with other dioxoruthenium(vi) complexes.14a,^32^,^33^,^34^

**Fig. 5:**
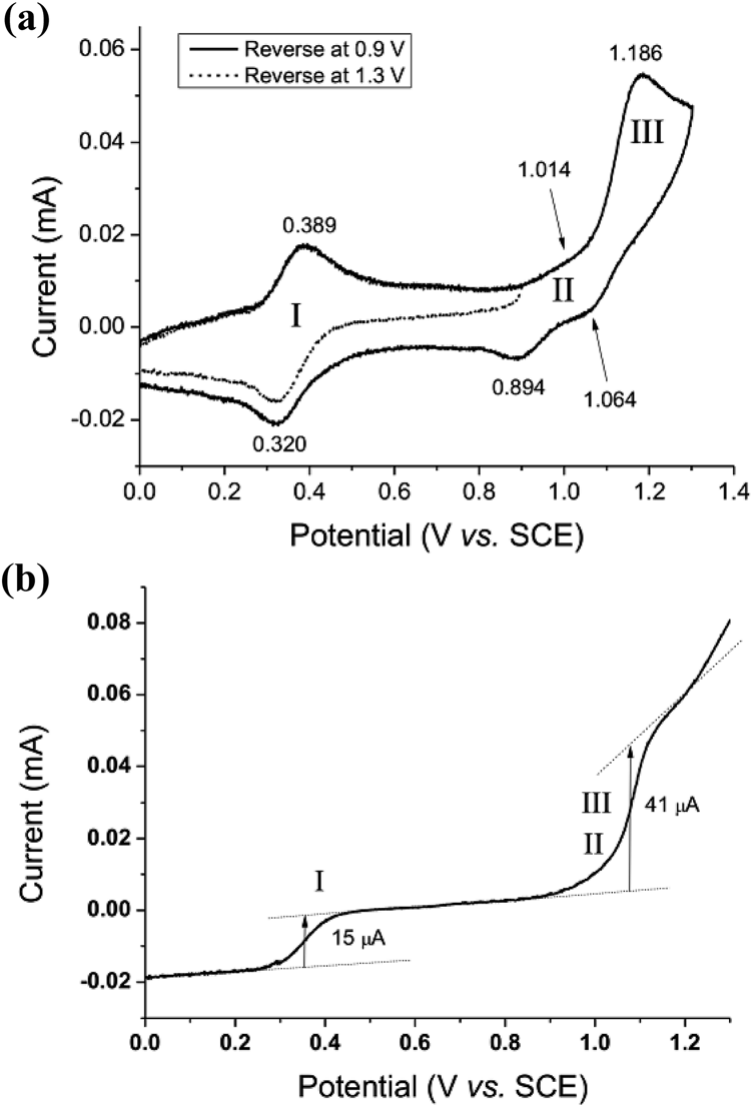
Cyclic voltammogram (a, upper) at 0.1 V s^–1^ and rotating-disk-electrode voltammogram (b, lower) at 100 rpm of **3c′** in 0.1 M CF_3_SO_3_H (pH 1). Working electrode: edge-plane pyrolytic graphite for CV; glassy carbon for RDEV.

**Scheme 4:**
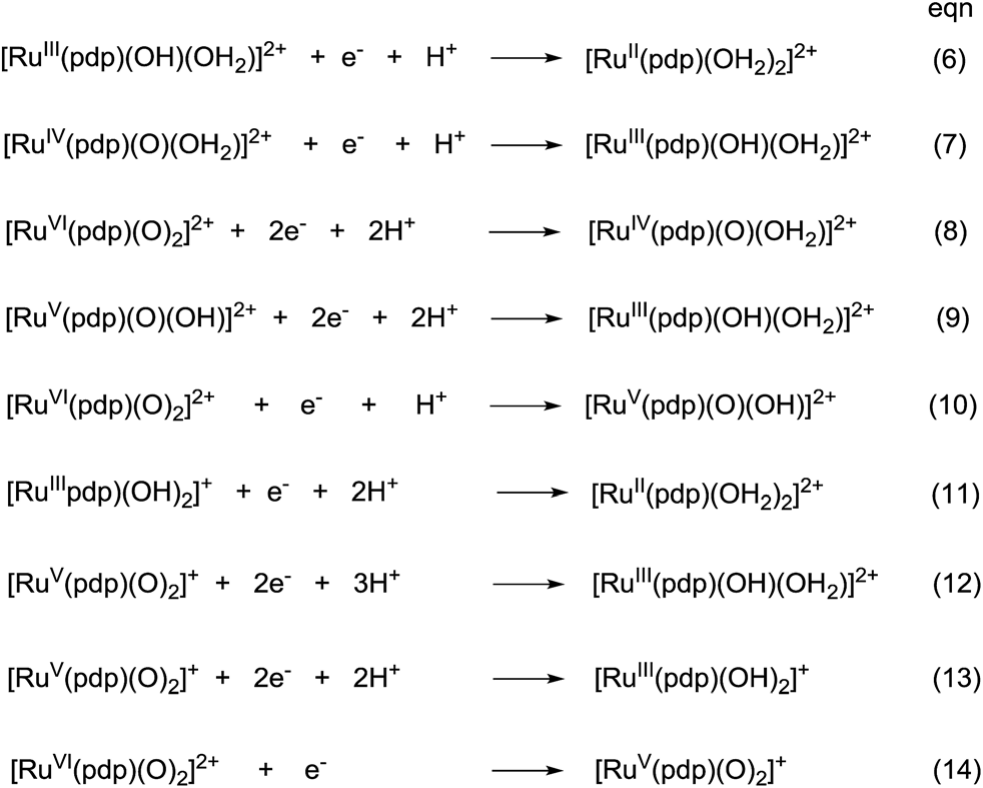
Proposed redox couples for *cis*-[(pdp)Ru^III^(O_3_SCF_3_)_2_]CF_3_SO_3_ (**3c′**) in different pH buffer solutions. The *cis*-sign is omitted for clarity.

The electrochemical properties of *cis*-[(pdp)Ru^III^(O_3_SCF_3_)_2_]-CF_3_SO_3_ (**3c′**, Scheme S1, ESI†) in 0.1 M CF_3_SO_3_H at pH 1 are reminiscent of that of **1b**. As depicted in Fig. 5a, **3c′** shows a reversible couple **I** at *E*_1/2_ = 0.36 V and a quasi-reversible couple **III** at *E*_1/2_ = 1.13 V (*E*_pa_ = 1.19 V) *vs.* SCE. Notably, at the foot of couple **III**, there is a less defined couple **II** at *E*_1/2_ = 0.95 V. Couple **I** (Δ*E*_p_ ∼ 60 mV; *i*_pa_/*i*_pc_ ∼ 1) is attributed to a Ru^III/II^ couple (eqn (6) in Scheme 4). Couple **II** is assigned as a Ru^IV/III^ couple (eqn (7)). Its much smaller current measured relative to the Ru^III/II^ couple is attributed to the rate-determining deprotonation of [Ru^III^(OH)] or [Ru^III^(OH_2_)] prior to the oxidation of Ru^III^ to Ru^IV^.^35^ Couple **III** is assigned as a Ru^VI/IV^ couple (eqn (8)).^36^ The natures of couples **I**, **II** and **III** were examined by rotating-disk electrode voltammetry (Fig. 5b), showing that the limiting current/number of electrons involved in couples **I** and (**II** and **III**) has a ratio of 1 to 2.7.^37^

The complex *cis*-[(pdp)Ru^II^(OH_2_)_2_](OTs)_2_ (**3c·OTs**) similarly shows a reversible couple at *E*_1/2_ = 0.36 V and an irreversible oxidation wave at *E*_pa_ = 1.14 V at pH 1 (Fig. S14a, ESI†).^38^ At pH 1, *cis*-[(bqcn)Ru^II^(OH_2_)_2_](OTs)_2_ (**5c·OTs**) shows a reversible Ru^III/II^ couple at *E*_1/2_ = 0.45 V and a shoulder oxidation wave at *E*_pa_ = 1.15 V (Fig. S14b, ESI†), while *cis*-[(Me_2_bqcn)Ru^II^(OH_2_)_2_](OTs)_2_ (**6c·OTs**) shows a reversible Ru^III/II^ couple at *E*_1/2_ = 0.49 V and a shoulder oxidation wave at *E*_pa_ = 1.08 V (Fig. S14c, ESI†). The σ-donating ability of the N_4_ ligands follows the order of mcp ≈ pdp > bqcn > Me_2_bqcn, as revealed by the *E*_1/2_ values of the Ru^III/II^ couples (Table 1).^39^ However, varying the structure of the N_4_ ligand has a minor effect on the redox potentials of the electrochemically generated *cis*-dioxoruthenium(vi) complexes (Δ*E*_pa_ ∼ 70 mV).

**Table 1:** Redox potentials of ruthenium N_4_ complexes in aqueous solution at pH 1 (ref. 39)

Complex	*E*_1/2_ of Ru^III/II^ couple (V *vs.* SCE)	Ru^VI/V^ or Ru^VI/IV^ oxidation (V *vs.* SCE)
**1b** (N_4_ = mcp)	0.37	*E*_1/2_ = 1.11
**3c′** (N_4_ = pdp)	0.36	*E*_1/2_ = 1.13
**5c·OTs** (N_4_ = bqcn)	0.45	*E*_pa_ = 1.15
**6c·OTs** (N_4_ = Me_2_bqcn)	0.49	*E*_pa_ = 1.08

**Fig. 6:**
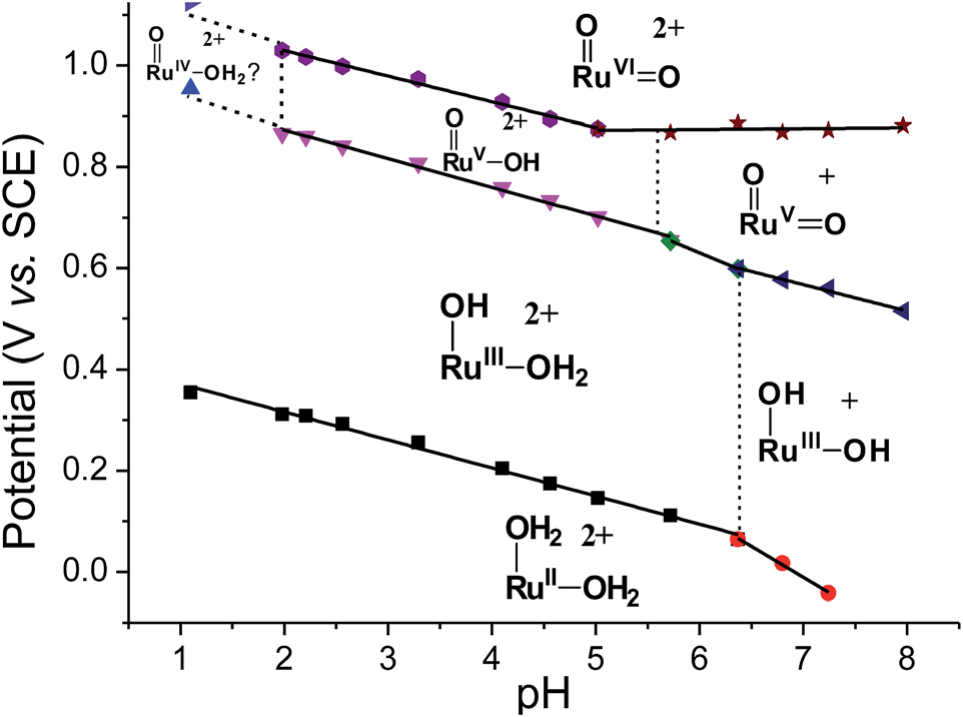
Pourbaix diagram of **3c′**. Data points at pH 1 were extracted from the cyclic voltammogram in 0.1 M CF_3_SO_3_H (*i.e.*, Fig. 5a), while data points at pH ≥ 2 were extracted from variable-pH measurements in Britton–Robinson buffer.

Variable-pH cyclic voltammetry of **3c′** was conducted in Britton–Robinson buffer.^40^–^42^ Selected voltammograms at pH = 2.56, 5.02 and 6.37 are displayed in Fig. S15 (ESI†). Above pH 1.98, couple **III** splits into two one-electron couples (Ru^V/IV^ and Ru^VI/V^); the former, which merges with couple **II** to form a new couple **IV**, can be assigned as a Ru^V/III^ couple (eqn (9)). The latter one is designated as couple **V** (eqn (10)). The Pourbaix diagram from pH 1 to 7.96 is shown in Fig. 6. For couple **I** (Ru^III/II^), there are two straight-line fragments with slopes of –56 and –122 mV per pH unit at 1 < pH < 6.37 and 6.37 < pH < 7.24, respectively, corresponding to the electrochemical reactions described in eqn (6) and (11). The breakpoint (pH = 6.4) of the plot for couple **I** is logically the p*K*_a_ value of *cis*-[(pdp)Ru^III^(OH)(OH_2_)]^2+^, which is comparable to that of *cis*-[(Tet-Me_6_)Ru^III^(OH)(OH_2_)]^2+^ (p*K*_a_ = 6.5).14*a* For couple **IV** (Ru^V/III^), three linear segments with slopes of –57, –85 and –52 mV per pH unit are found at 1.98 < pH < 5.72, 5.72 < pH < 6.37 and 6.37 < pH < 7.96, respectively. The corresponding electrochemical reactions are described in eqn (9), (12) and (13). For couple **V** (Ru^VI/V^), its potential shifts cathodically with a slope of –51 mV pH^–1^ at 1.98 < pH < 5.02. This is in line with its one-proton one-electron nature (equation (10)). At 5.02 < pH < 7.96, it becomes insensitive to pH, suggesting a one-electron process that does not involve proton loss (equation (14)). From this observation, together with the breakpoint of the plot of couple **IV**, the p*K*_a_ value of *cis*-[(pdp)Ru^V^(O)(OH)]^2+^ is estimated to be 5.6.

**Scheme 5:**
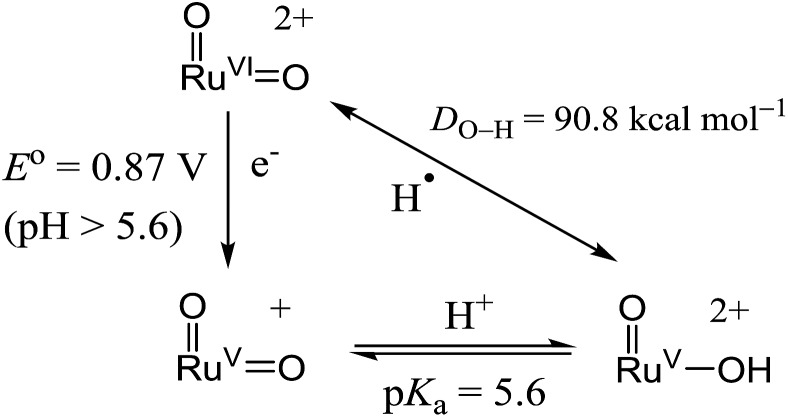
Thermochemical cycle of *cis*-[(pdp)Ru^VI^(O)_2_]^2+^.

With the above electrochemical information in hand, the bond dissociation energy (*D*_O–H_) for *cis*-[(pdp)Ru^V^(O)(O–H)]^2+^ to form *cis*-[(pdp)Ru^VI^(O)_2_]^2+^ can be obtained from eqn (15), based on the thermochemical method developed by Mayer and Bordwell.^43^,^44^ The *D*_O–H_ value is calculated to be 90.8 kcal mol^–1^ for *cis*-[(pdp)Ru^VI^(O)_2_]^2+^ (Scheme 5) and 90.1 kcal mol^–1^ for *cis*-[(mcp)Ru^VI^(O)_2_]^2+^.

15 *D*_O–H_ = 23.06*E*° + 1.37p*K*_a_ + *C*

^45^

### Isolation or generation of *cis*-dioxoruthenium(vi) complexes *via* chemical oxidation

**Fig. 7:**
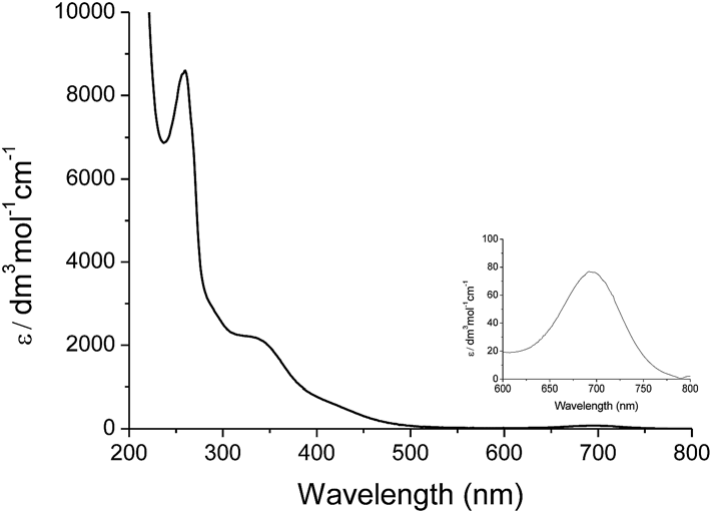
UV-Vis absorption spectrum of *cis*-[(mcp)Ru^VI^(O)_2_](ClO_4_)_2_ (**1e**) in acetonitrile.

**Scheme 6:**
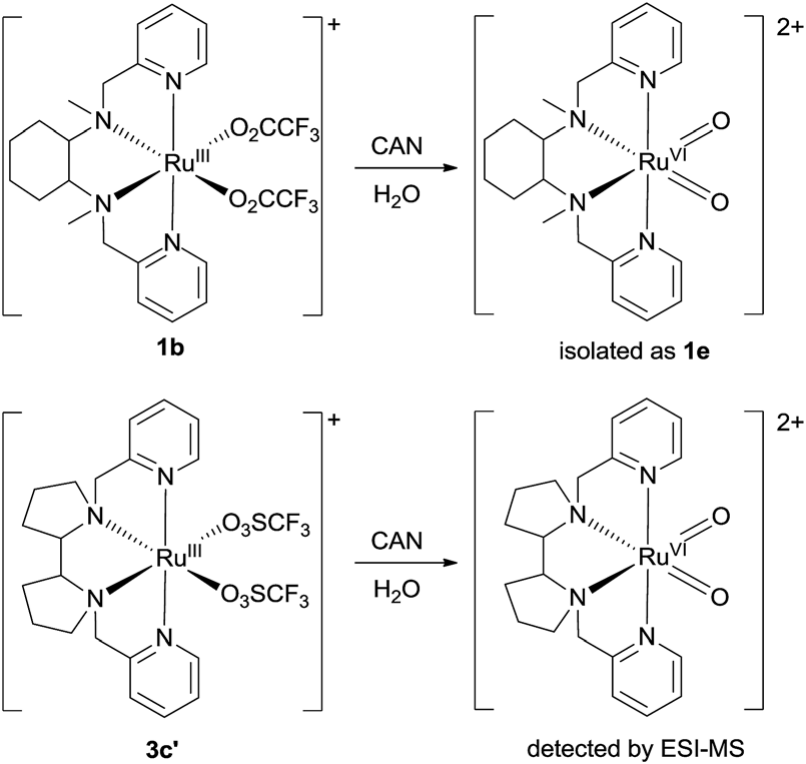
Preparation of **1e** and generation of *cis*-[(pdp)Ru^VI^(O)_2_]^2+^.

Treatment of *cis*-[(mcp)Ru^III^(O_2_CCF_3_)_2_]^+^ (**1b**) with excess CAN in aqueous solution gave *cis*-[(mcp)Ru^VI^(O)_2_]^2+^ (**1e**), which was isolated as a pale green perchlorate salt in 66% yield (Scheme 6, see Experimental section for details). The UV-visible absorption spectrum of a freshly prepared solution of *cis*-[(mcp)Ru^VI^(O)_2_](ClO_4_)_2_ (Fig. 7) in acetonitrile shows a prominent absorption peak at *λ*_max_ = 260 nm (*ε* = 8700 dm^3^ mol^–1^ cm^–1^), a broad shoulder band at 340 nm (*ε* = 2210 dm^3^ mol^–1^ cm^–1^) and a weak absorption band at 700 nm (*ε* = 80 dm^3^ mol^–1^ cm^–1^). The high-resolution ESI mass spectrum of **1e** shows a prominent ion species centred at *m*/*z* = 229.0631 that matches the formulation and the isotope distribution pattern of [(mcp)Ru(O)_2_]^2+^ (Fig. S16, ESI†). The IR spectrum of **1e** shows two peaks at 845 and 868 cm^–1^, which are assigned to the symmetric and asymmetric stretches of the *cis*-dioxoruthenium(vi) moiety.14a,15a,16a Complex **1e** is diamagnetic, as revealed by its ^1^H NMR signals. Notably, **1e** is stable at –15 °C under argon for a few hours but decomposes in aqueous *tert*-butanol or acetonitrile within 30 min to give a dark brown solution, the ESI-MS analysis of this solution showed peaks centred at *m*/*z* = 460.1, which corresponds to [(mcp)Ru(OH)_2_]^+^ in aqueous *tert*-butanol, and *m*/*z* = 254.1, which corresponds to [(mcp)Ru(NCMe)_2_]^2+^ in acetonitrile. In aqueous solution at pH 1, *cis*-[(mcp)Ru^VI^(O)_2_](ClO_4_)_2_ (**1e**) shows an identical cyclic voltammogram as that as **1b**. The cyclic voltammogram of **1e** in acetonitrile shows a reversible one-electron couple at *E*_1/2_ = 0.78 V *vs.* Ag/AgNO_3_ (0.1 M in MeCN), attributed to a Ru^VI/V^ couple: *cis*-[(mcp)Ru^VI^(O)_2_]^2+^ + e^–^ → [(mcp)Ru^V^(O)_2_]^+^. Based on this redox potential, **1e** is a stronger oxidant than *cis*-[(Tet-Me_6_)Ru^VI^(O)_2_](ClO_4_)_2_ (*E*_1/2_ = 0.53 V *vs.* Ag/AgNO_3_).14*a*

**Fig. 8:**
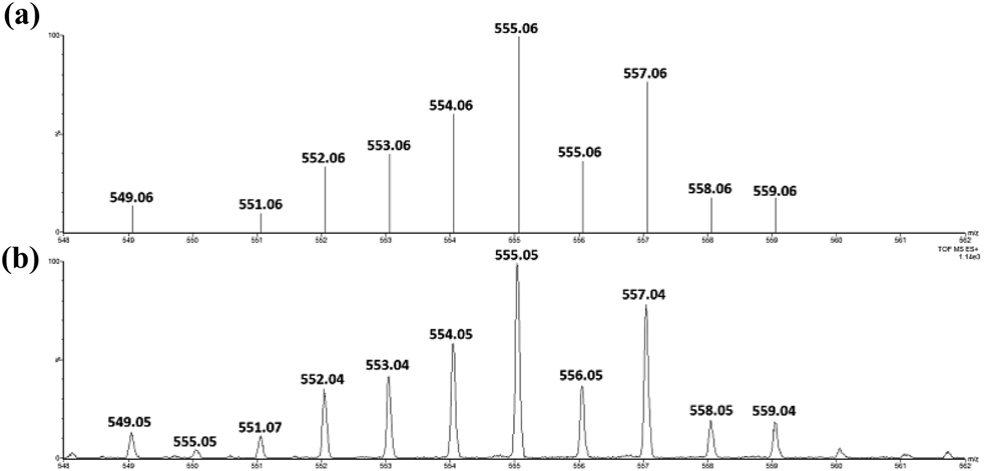
(Upper) Simulated ESI-MS pattern of {[(pdp)Ru^VI^(O)_2_]ClO_4_}^+^. (Lower) Experimental ESI-MS signals for a reaction mixture of **3c·CF_3_SO_3_** and 6 equiv. of Ce^IV^(ClO_4_)_4_, [Ru] = 1 × 10^–4^ M.

Similar oxidation of *cis*-[(pdp)Ru^III^(O_3_SCF_3_)_2_]^+^ (**3c′**) or *in situ* generated *cis*-[(pdp)Ru^II^(OH_2_)_2_]^2+^ by CAN did not furnish isolable *cis*-[(pdp)Ru^VI^(O)_2_](ClO_4_)_2_ (Scheme 6). Upon addition of the CAN solution into an ice-cooled solution of **3c′**, a dark brown solution resulted, and subsequent addition of ClO_4_^–^ or PF_6_^–^ did not induce solid formation. Small-scale reactions of **3c·CF_3_SO_3_** with a Ce^IV^ oxidant were performed in water and monitored by high-resolution ESI-MS. Under dilute conditions ([Ru] = 1 × 10^–4^ M), treatment of **3c·CF_3_SO_3_** with 4 equiv. of Ce^IV^(ClO_4_)_4_ generated predominant ruthenium signals at *m*/*z* = 220.05 and 228.56. These signals are attributed to [(pdp)Ru^IV^(O)]^2+^ and [(pdp)Ru^V^(O)(OH)]^2+^ species (Fig. S17, ESI†). When a slight excess of Ce^IV^(ClO_4_)_4_ (6 equiv.; 150% for a 4e^–^ oxidation process) was employed, a new signal that corresponds to {[(pdp)Ru^VI^(O)_2_]ClO_4_}^+^ was observed at *m*/*z* = 555.05 (Fig. 8). Notably, its signal intensity dropped significantly after *ca.* 1 min (Fig. S18, ESI†). A *cis*-dioxo-Ru(v) species was also detected at *m*/*z* = 456.10 (Fig. S19a, ESI†), with its signal intensity remaining constant for at least 3 min (Fig. S19b†). At a higher concentration of **3c·CF_3_SO_3_** ([Ru] = 1 × 10^–3^ M), a complicated spectrum dominated by noise signals was obtained with just 4 equiv. of Ce^IV^(ClO_4_)_4_. Most likely, the decomposition of Ru(pdp) complexes under oxidizing condition is significantly fast with [Ru] ≥ 1 mM. This may account for the difficult isolation of *cis*-[(pdp)Ru^VI^(O)_2_](ClO_4_)_2_ in the large-scale preparative experiment.

### Stoichiometric oxidation of hydrocarbons by *cis*-[(mcp)Ru^VI^(O)_2_](ClO_4_)_2_ (**1e**)

The results of the electrochemical studies suggest that *cis*-[(mcp)Ru^VI^(O)_2_](ClO_4_)_2_ (**1e**) is a strong oxidant (*E*° = 1.11 V *vs.* SCE at pH 1). In aqueous *tert*-butanol, freshly prepared **1e** could stoichiometrically oxidize cyclooctene to give a mixture of *cis*-cyclooctane-1,2-diol (27%) and 1,8-octanedialdehyde (70%) (Table 2, entry 1).^46^ Compared with our previous works, [(Me_3_tacn)Ru^VI^(O)_2_(O_2_CCF_3_)]ClO_4_ oxidized cyclooctene stoichiometrically to give *cis*-cyclooctane-1,2-diol and 1,8-octanedialdehyde in 85% and 5% yields, respectively, whereas use of *cis*-[(Tet-Me_6_)Ru^VI^(O)_2_](ClO_4_)_2_ gave 22% *cis*-cyclooctane-1,2-diol and 60% 1,8-octanedialdehyde.^47^ Apart from the organic products, a green ruthenium compound was isolated at the end of the reaction of **1e** with cyclooctene. ESI-MS analysis revealed a prominent ion peak at *m*/*z* = 460.1; its *m*/*z* ratio and isotopic distribution pattern are consistent with a [(mcp)Ru^III^(OH)_2_]^+^ formulation.

**Table 2:** Stoichiometric oxidation of alkenes by *cis*-[((*R*,*R*)-mcp)Ru^VI^(O)_2_](ClO_4_)_2_ (1e*) in aqueous *tert*-butanol^*a*^

Entry	Alkene subtrate	Product(s)	% Yield^*b*^ (% ee)
1	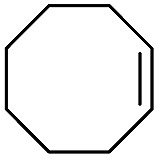	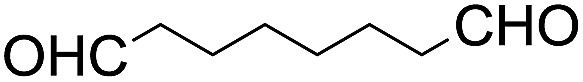	70^*c*^
		*cis*-Diol	27
2	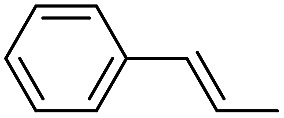	*syn*-Diol	20 (24)
		*anti*-Diol	28 (35)
		PhCHO	45^*c*^
3	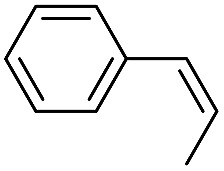	*syn*-Diol	21 (30)
		*anti*-Diol	25 (36)
		PhCHO	52^*c*^
4	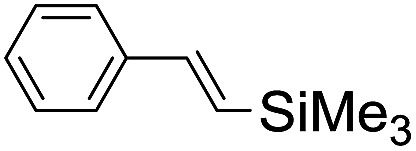	*syn*-Diol	19 (28)
		*anti*-Diol	26 (33)
		PhCHO	53^*c*^
5	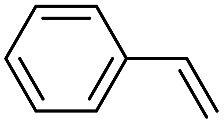	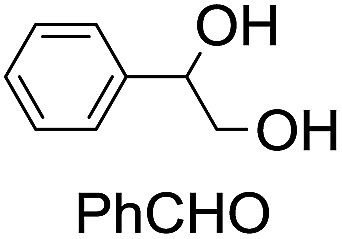	42 (27)
			50^*c*^
6	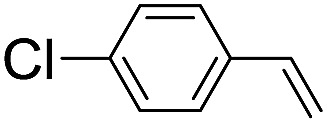	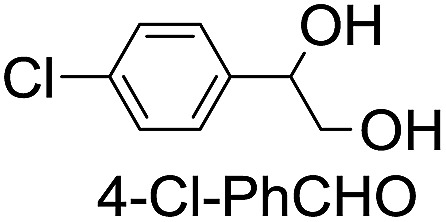	39 (33)
			51^*c*^
7	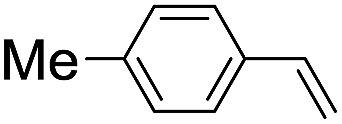	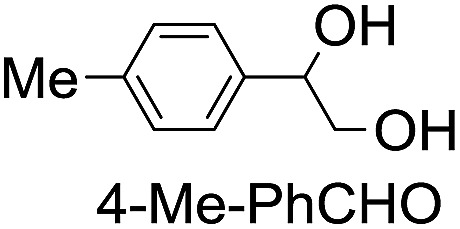	43 (28)
			48^*c*^
8	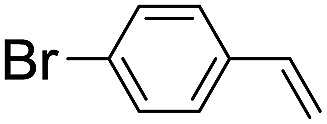	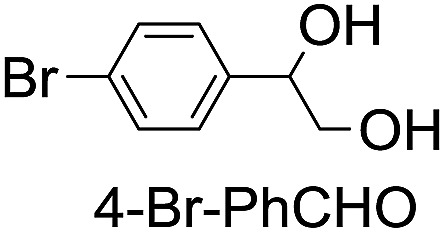	45 (34)
			47^*c*^

^*a*^Reaction conditions: 1e* (0.3 mmol), substrate (30 mmol), ^*t*^BuOH/H_2_O (5 : 1 v/v, 12 mL), under argon, room temperature, and 30 min.

^*b*^Isolated yield, calculated as mmol of product per mmol of **1e**.

^*c*^Determined by GC.

Using chiral (*R*,*R*)-mcp as a ligand, the chiral *cis*-dioxoruthenium(vi) complex, *cis*-[((*R*,*R*)-mcp)Ru^VI^(O)_2_](ClO_4_)_2_ (1e*), was prepared. Several stoichiometric alkene oxidation reactions were performed by reacting 1e* (0.3 mmol) with excess alkene substrate (30 mmol, 100 equiv.) in a degassed (5 : 1 v/v) *tert*-butanol/H_2_O mixture (12 mL) under argon at room temperature for 30 min (Table 2). Aryl alkenes were oxidized to their corresponding diols (39–48% yields) with ee values ranging from 24 to 36%, accompanied by the formation of C=C bond cleavage products in considerable amounts (45–53%). In the reaction of 1e* with styrene, for instance, a 42% yield of styrene glycol (27% ee) and 50% yield of benzaldehyde were obtained (entry 5, Table 2). Similarly, *trans*-β-(trimethylsilyl)styrene reacted with 1e* to afford a 19% yield of *syn*-diol (28% ee) and 26% yield of *anti*-diol (33% ee) along with a 53% yield of benzaldehyde (entry 4, Table 2). There is no major difference in the reactions of 1e* with *trans*-β-methylstyrene and with *cis*-β-methylstyrene, which afforded the enantio-enriched *syn*-diol in 20% yield (24% ee) and 21% yield (30% ee), *anti*-diol in 28% yield (35% ee) and 25% yield (36% ee), benzaldehyde in 45% and 52% yields, respectively (entries 2 and 3, Table 2). The effects of *para*-substituents on the enantioselectivity of *p*-substituted styrenes in the reaction with 1e* were examined (entries 5–8, Table 2); the *para*-substituents CH_3_, Cl and Br had no significant effect on either the yields (39–45%) or ee (28–34%) of the diol products.

The stoichiometric oxidations of alcohols and alkanes by **1e** were studied. When **1e** was treated with benzyl alcohol (100 equiv.) in acetonitrile at room temperature for 30 min, benzaldehyde was formed in 90% yield (Table 3, entry 1). Similarly, other primary alcohols such as 1-heptanol and 1-octanol were oxidized by **1e** to give a mixture of aldehyde and carboxylic acid (entries 2 and 3, Table 3). Under these conditions, cyclooctene reacted with **1e** to afford cyclooctene oxide and 1,8-octanedialdehyde in 30% and 58% yields, respectively (entry 4, Table 3). Complex **1e** could also oxidize saturated C–H bonds. For instance, ethylbenzene (BDE_C–H_ = 85.4 kcal mol^–1^)^48^ was oxidized by **1e** in acetonitrile to give acetophenone (55% yield) and 1-phenylethanol (26% yield) (entry 5, Table 3). Notably, cyclohexane (BDE_C–H_ = 99.5 kcal mol^–1^)^48^ was oxidized to give cyclohexanone in 62% yield (entry 6, Table 3). Similar to the reported *cis*-dioxoruthenium(vi) complexes,14a,16a when adamantane was employed as a substrate, C–H oxidation occurred primarily at the 3° carbon; 1-adamantanol was formed as the sole product in 58% yield (entry 7, Table 3). The oxidation of *cis*-4-methylcyclohexyl benzoate afforded the tertiary alcohol in moderate yield (66%) with complete retention of the configuration; no epimerized product was observed (entry 8, Table 3). Reaction of 1e* with the two racemic substrates in entries 9 and 10 (Table 3) predominantly gave oxygenated products at the tertiary C–H bonds; however, chiral HPLC analysis of the tertiary alcohol product revealed no kinetic resolution effect (ee <2%). These organic transformations were accompanied by the reduction of *cis*-dioxoruthenium(vi) to *cis*-[(mcp)Ru^II^(NCMe)_2_](ClO_4_)_2_ (**1d**), which was isolated and characterized (ESI†).

**Table 3:** Stoichiometric organic oxidations by *cis*-[(mcp)Ru^VI^(O)_2_](ClO_4_)_2_ (**1e**) in acetonitrile^*a*^

Entry	Substrate	Product(s)	Yield^*b*^ (%)
1	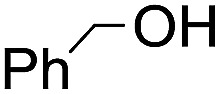	PhCHO	90^*c*^
2	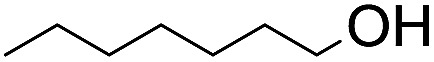	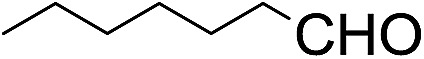	60
		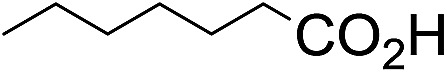	22
3	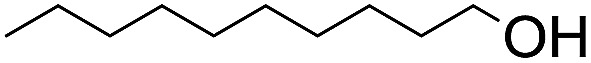	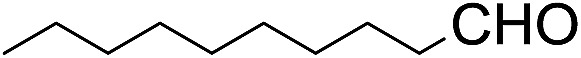	55
		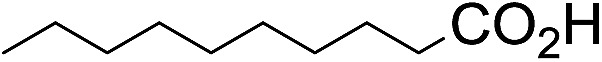	26
4	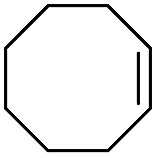	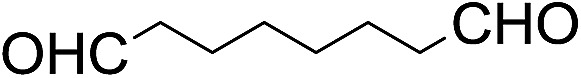	58^*c*^
		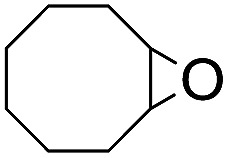	30
5	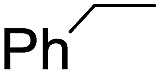	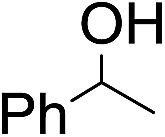	26^*c*^
		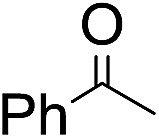	55^*c*^
6	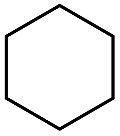	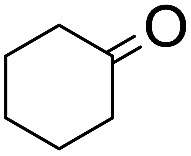	62^*c*^
7	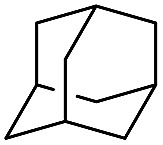	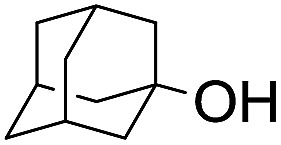	58
8	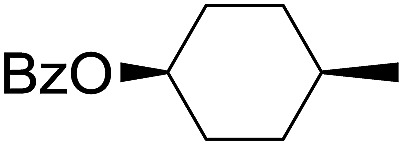	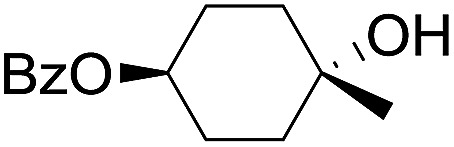	66
9^*d*^	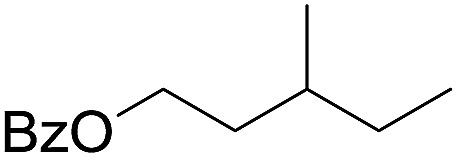	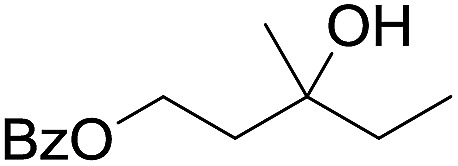	58
10^*d*^	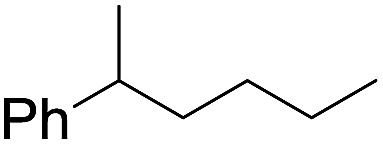	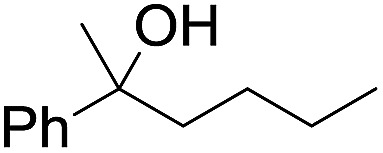	60

^*a*^Reaction conditions: **1e** (0.3 mmol), substrate (30 mmol), MeCN (12 mL), under argon, room temperature, and 30 min.

^*b*^Isolated yield, calculated as mmol of product per mmol of **1e**.

^*c*^Determined by GC.

^*d*^ 1e* instead of **1e** was used.

### Catalytic oxidation of alkanes with CAN mediated by *cis*-[(pdp)Ru^II^(OH_2_)_2_]^2+^ (**3c**)

The catalytic activities of the *cis*-[(mcp)Ru^III^(O_2_CCF_3_)_2_]^+^ (**1b**) and *cis*-diaquoruthenium(ii) complexes (**3c–6c**) towards the hydroxylation of C(sp^3^)–H bonds were examined using CAN as a terminal oxidant. *cis*-1,2-Dimethylcyclohexane (**S1**) was chosen as an initial test substrate (Table 4). The reaction of **S1** with 2 mol% **3c·OTs** and 3 equiv. of CAN for 15 min at room temperature in aqueous *tert*-butanol afforded a tertiary alcohol product (**P1**) in 64% yield based on 80% conversion (entry 2, Table 4).^49^ The stereogenic centres are retained in the alcohol product, indicating that the hydroxylation reaction does not involve long-lived carbon-based radicals that can epimerize. Among the screened ruthenium catalysts, **3c·OTs** showed the highest catalytic activity. When **4c·OTs** or **6c·OTs** was employed as the catalyst, particularly, the substrate conversion was <10% (entries 3 and 5, Table 4).^50^ Therefore, subsequent studies focused on the use of **3c·OTs** as a catalyst. In a control experiment, in which the ruthenium catalyst was replaced by [Ru^II^(OH_2_)_6_](OTs)_2_, **S1** remained intact for a 30 min reaction (Table 5, entry 2). Subsequent addition of **3c·OTs** to this reaction mixture followed by stirring for 15 min afforded **P1** in 64% yield based on 61% conversion.

**Table 4:** Oxidation of *cis*-1,2-dimethylcyclohexane with CAN catalysed by **1b** and *cis*-[(N_4_)Ru^II^(OH_2_)_2_]^2+^ complexes^*a*^

Entry	Catalyst	Reaction time (min)	Conversion (%)	Product yield (%) based on conversion
1	**1b**	15	60	62
2	**3c·OTs**	15	80	64
3	**4c·OTs**	30	8	55
4	**5c·OTs**	15	73	61
5	**6c·OTs**	30	6	61

^*a*^Reaction conditions: substrate (0.25 mmol), catalyst (2 mol%), CAN (0.75 mmol), ^*t*^BuOH/H_2_O (1 : 1 v/v, 4 mL), and room temperature.

**Table 5:** Oxidation of tertiary and benzylic C–H bonds with CAN catalysed by **3c·OTs**^*a*^

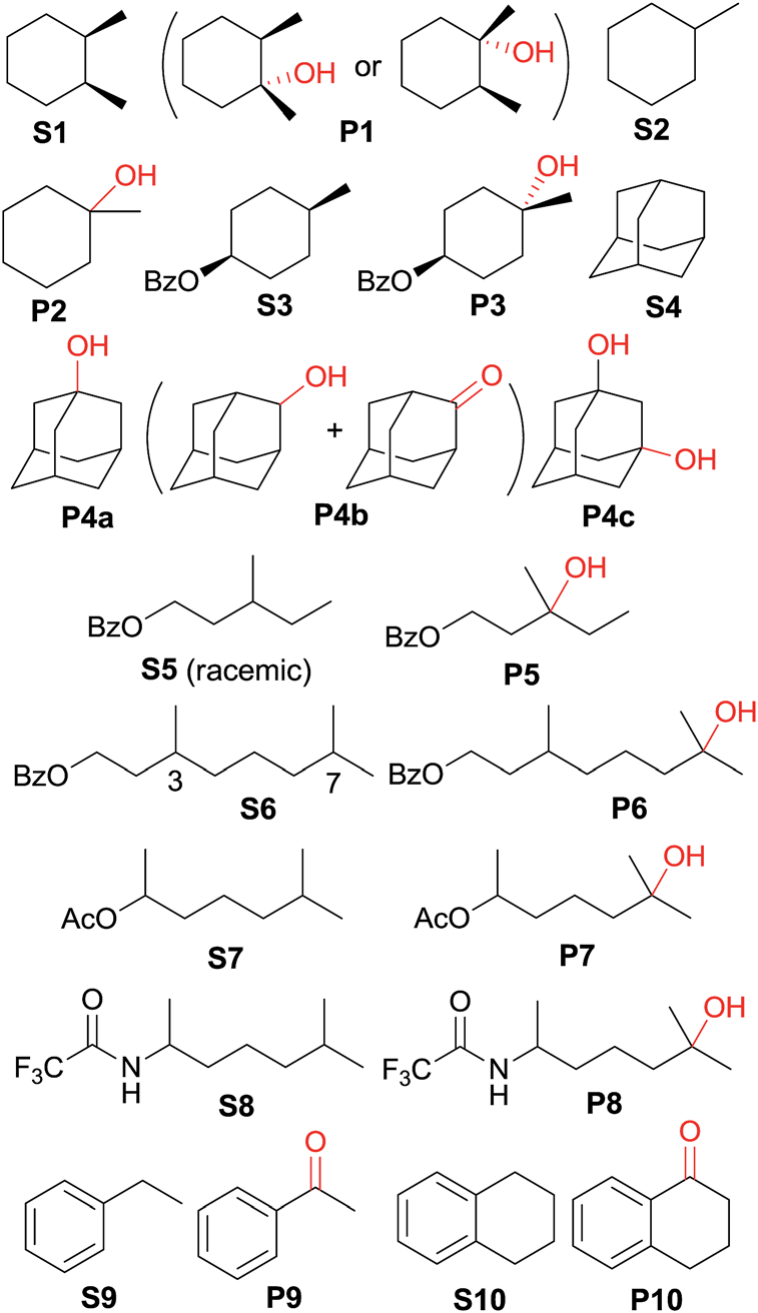				
Entry	Substrate	Reaction time	Conversion (%)	Products (yield in % based on conversion)
1	**S1**	15 min	80	**P1** (64)
2^*b*^	**S1**	30 min	<1	—
3^*c*^	**S2**	45 min	51	**P2** (96)
4^*d*^	**S3**	1 h	52	**P3** (84)
5^*c*^	**S4**	1.5 h	74	**P4a** (47), **P4b** (3), **P4c** (32)
6^*d*^	**S5**	1 h	59	**P5** (83)
7	**S6**	1.5 h	40	**P6 (**80)
8	**S7**	1 h	60	**P7** (85)
9	**S8**	1 h	65	**P8** (89)
10	**S9**	15 min	28	**P9** (91)
11^*c*^ ^,^^*e*^	**S10**	40 min	84	**P10** (89)

^*a*^Reaction conditions: substrate (0.25 mmol), catalyst (2 mol%), CAN (0.75 mmol), ^*t*^BuOH/H_2_O (1 : 1 v/v, 4 mL), and room temperature.

^*b*^[Ru^II^(OH_2_)_6_](OTs)_2_ was used as the catalyst.

^*c*^ ^*t*^BuOH/H_2_O (3 : 1 v/v, 4 mL) was used as the solvent.

^*d*^CF_3_CH_2_OH/H_2_O (3 : 1 v/v, 4 mL) was used as the solvent.

^*e*^1.5 mmol CAN was used.

Oxidation of methylcyclohexane (**S2**) gave a tertiary alcohol product (**P2**) with high selectivity (96%) based on 52% conversion (Table 5, entry 3). Similarly, **S3** was oxidized to **P3** with good selectivity (entry 4, Table 5). For the oxidation of adamantane (**S4**), apart from ordinary oxygenation products, such as Ad-1-ol (**P4a**, 47% yield) and “Ad-2-ol + Ad-2one” (**P4b**, 3% yield), adamantan-1,3-diol (**P4c**) was also formed in 32% yield (entry 5, Table 5). Most likely, the initial hydroxylation of **S4** gives **P4a**; the latter, being more soluble, was efficiently further hydroxylated to yield **P4c**.^51^ The normalized 3°/2° selectivity is as high as 79 : 1, showing the strong preference of the active oxidant to attack 3° over 2° C–H bonds. Following this preference, the oxidation of racemic **S5** produced **P5** in 48% isolated yield (entry 6, Table 5).^52^ Compound **S6** has two possible sites (C3 and C7) for tertiary C–H hydroxylation. Analysis of the crude reaction mixture by ^1^H NMR spectroscopy revealed the C7 : C3 selectivity to be a ratio of >10 : 1. After purification, a C7-hydroxylated product (**P6**) was obtained in 80% yield (entry 7, Table 5). Reactions of **S7** and **S8** similarly occurred at the 3° C–H bond which were remote from the electron-withdrawing ester/amide groups, in 51% and 58% isolated yields, respectively (entries 8 and 9, Table 5). In entries 10 and 11 (Table 5), the benzylic C–H bonds in ethylbenzene and tetralin were oxidized to give acetopheone (**P9**) and tetralone (**P10**), respectively. No aromatic ring degradation products were found. Thus, the possible involvement of RuO_4_ was unlikely as it is known to degrade aromatic rings.^53^ A kinetic isotope effect (KIE) of *k*_H_/*k*_D_ = 5.2 was found in the competitive oxidation of an equimolar mixture of ethylbenzene and *d*_10_-ethylbenzene, indicative of C–H bond cleavage in the rate-determining step (RDS) or in a product-determining step following the RDS.^54^

For more complex substrates, the catalyst loading was increased to 5 mol% to furnish oxidation products in isolated yields ranging from 37% to 76% (55–93% based on conversion, Table 6). In general, sterically unhindered tertiary C–H bonds were preferred over unactivated methylene centres (entries 1 and 4, Table 6). For the oxidation of **S12** that contains both tertiary and benzylic C–H bonds, only the ketone product **P12** was formed (entry 2, Table 6). Notably, the reaction of **S13** gave desaturation product **P13** (entry 4, Table 6), presumably *via* an alcohol intermediate.^55^

**Table 6:** Oxidation of pharmaceutical ingredients and natural product derivatives with CAN catalysed by **3c·OTs**^*a*^

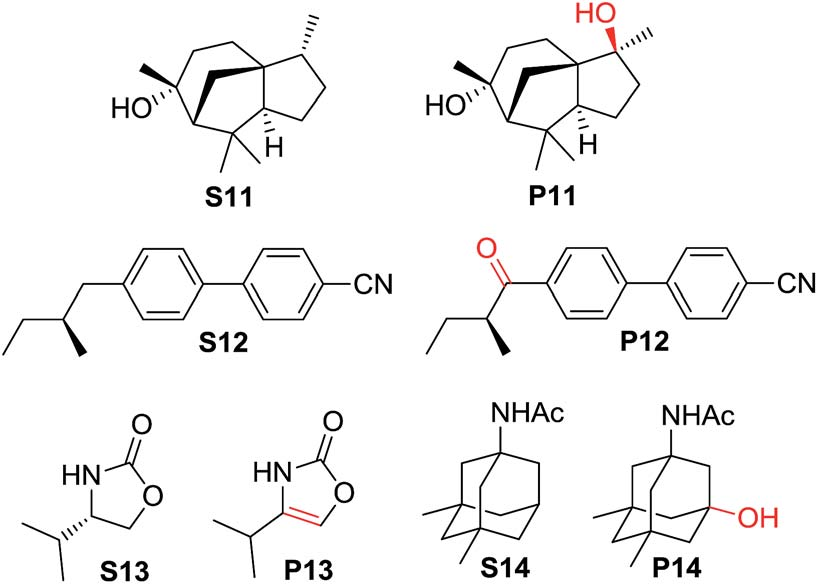				
Entry	Substrate	Reaction time	Conversion (%)	Products (yield in % based on conversion)
1	**S11**	40 min	50	**P11** (84)
2	**S12**	40 min	82	**P12** (93)
3	**S13**	20 min	68	**P13** (55)
4	**S14**	13 h	73	**P14** (78)

^*a*^Reaction conditions: substrate (0.2 mmol), catalyst (5 mol%), CAN (1.2 mmol), ^*t*^BuOH/H_2_O (1 : 1 v/v, 4 mL), and room temperature.

Interestingly, this catalytic protocol was also found capable of oxidizing strong secondary and primary C–H bonds of light alkanes (Table 7). The oxidation of cyclooctane (BDE_C–H_ = 95.7 kcal mol^–1^)^48^ for 1.5 h gave cyclooctanone in 95% yield based on 40% conversion (entry 1, Table 7). Similarly, cyclohexane (BDE_C–H_ = 99.5 kcal mol^–1^)^48^ was oxidized to cyclohexanone with a turnover number (TON) of 9 (entry 2, Table 7). The oxidation of propane (BDE_C–H_ = 98.1 kcal mol^–1^)^48^ afforded acetone with a TON = 8 for a 3 h reaction (entry 3, Table 7). Lastly, oxidation of ethane (BDE_C–H_ = 100.5 kcal mol^–1^)^48^ afforded acetic acid with a TON = 3 (entry 4, Table 7).^56^

**Table 7:** Oxidation of secondary and primary C–H bonds with CAN catalysed by **3c·OTs**^*a*^

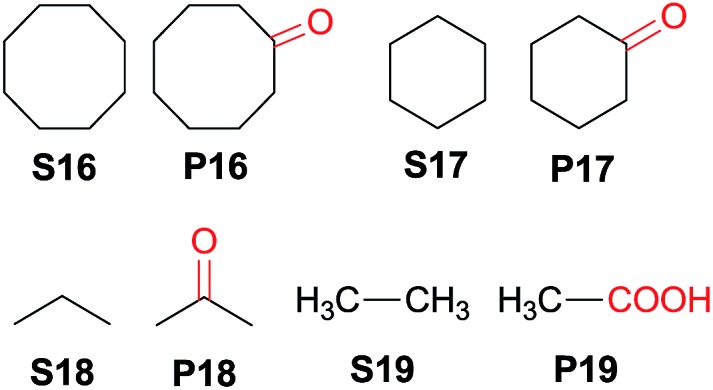				
Entry	Substrate	Reaction time (h)	Conversion (%)	Products (yield in % based on conversion)
1^*b*^	**S16**	1.5	40	**P16** (95)
2^*b*^ ^,^^*c*^	**S17**	1.5	—^*d*^	**P17** (TON = 9)
3^*e*^	**S18**	3	—	**P18** (TON = 8)
4^*e*^	**S19**	3	—	**P19** (TON = 3)

^*a*^Reaction conditions: substrate (0.25 mmol), catalyst (2 mol%), CAN (0.75 mmol), ^*t*^BuOH/H_2_O (1 : 1 v/v, 4 mL), and room temperature.

^*b*^ ^*t*^BuOH/H_2_O (3 : 1 v/v, 4 mL) was used as the solvent.

^*c*^1.5 mmol CAN was used.

^*d*^Conversion was not determined because of the high volatility of the substrate.

^*e*^Gaseous substrate used in excess (100 psi).

## General remarks/discussion

### General properties of the ruthenium N_4_ complexes

Two series of ruthenium complexes, *cis*-[(N_4_)Ru^III^Cl_2_]^+^ (**1a–4a**) and *cis*-[(N_4_)Ru^II^(OH_2_)_2_]^2+^ (**3c–6c**), were prepared. Owing to the lability of aqua ligands, [Ru^II^(OH_2_)_6_](OTs)_2_ is an efficient precursor for the synthesis of *cis*-[(N_4_)Ru^II^(OH_2_)_2_]^2+^ complexes (**3c–6c**). The *cis*-diaquoruthenium(ii) complexes were isolated as ditosylate (OTs^–^) salts and are air sensitive. In aqueous solutions under aerobic conditions, they are susceptible to oxidation, as determined by the depletion of the characteristic MLCT transition band at 360–480 nm. Accompanying the UV-Vis spectral changes, the predominant species observed in ESI-MS analysis changed from [(N_4_)Ru^II^(OTs)]^+^ to [(N_4_)Ru^III^(OH)_2_]^+^. Complexes without *ortho*-methyl substituents on the pyridyl/quinolyl moieties (**3c** and **5c**) are less prone to aerobic oxidation; the process requires hours to complete. In contrast, complexes **4c** and **6c** are readily oxidized to Ru(iii) species within 1 h.

The structural analyses of the *cis*-[(N_4_)Ru^III^Cl_2_]^+^ complexes by X-ray crystallography show that the *cis*-α configuration is the predominantly preferred geometry. ^1^H NMR spectroscopy of the *cis*-[(N_4_)Ru^II^(OH_2_)_2_]^2+^ complexes in CD_3_CN or the bis(acetonitrile)ruthenium(ii) complex revealed that the coordination geometry depends on the ligand structure. In particular, the bqcn ligand coordinates to the ruthenium centre in an unselective manner affording a mixture of *cis*-α and *cis*-β isomers, which do not interconvert in acetonitrile solution. In the X-ray crystal structures of *cis*-α-**5d** and *cis*-β-**5d**, the *N*-methyl groups have different orientations (*anti* or *syn*). Thus, interconversion between the two isomers requires (i) breakage of the Ru–N(quinolyl) bond, (ii) breakage of the Ru–N(amine) bond, and (iii) epimerization of the *N*-methyl group followed by migration of the acetonitrile ligand. These are expected to have large kinetic barriers, therefore, interconversion between the two isomeric forms is slow, and the ligand topology is likely determined at the synthetic stage of **5c·OTs**. Similar arguments have been addressed by Nam, Shin and co-workers; they found that *cis*-α- or *cis*-β-[(bqcn)Fe^II^(NCMe)_2_]^2+^ could be independently obtained with different synthetic methods and that these isomers do not interconvert in solution at room temperature.^57^

### Electrochemistry/reduction potentials

Aqueous electrochemical measurements (at pH 1) of *cis*-[(mcp)Ru^III^(O_2_CCF_3_)_2_]ClO_4_ (**1b**) and *cis*-[(pdp)Ru^III^(O_3_SCF_3_)_2_]-CF_3_SO_3_ (**3c′**) revealed the strong oxidizing powers of their corresponding *cis*-dioxoruthenium(vi) species. The highly anodic redox potentials (*E*° = 1.11–1.13 V *vs.* SCE) are comparable to those of *cis*-[(6,6′-Cl_2_bpy)_2_Ru^VI^(O)_2_]^2+^ (1.17 V)^58^ and electrochemically generated *cis*-[(TPA)Ru^VI^(O)_2_]^2+^ (1.1 V, TPA = tris(2-pyridylmethyl)amine).^42^

The aqueous electrochemical data allow the determination of the hydrogen-atom affinity of the *cis*-dioxoruthenium(vi) complexes. The *D*_O–H_ values are calculated to be 90.8 kcal mol^–1^ for *cis*-[(pdp)Ru^VI^(O)_2_]^2+^ and 90.1 kcal mol^–1^ for *cis*-[(mcp)Ru^VI^(O)_2_]^2+^. Referring to Table 8, these values are comparable to those of *cis*-[(bpy)_2_Ru^VI^(O)_2_]^2+^ (93.5 kcal mol^–1^)^17^ and [(TSMP)Fe^IV^(O)] (90 kcal mol^–1^, H_2_TSMP = *meso*-tetrakis(sulfonatomesityl)porphyrin),^59^,^60^ but are considerably larger than those of several (mono)oxoruthenium(iv) complexes (82.7–84.8 kcal mol^–1^),^61^,^62^ *trans*-dioxoruthenium(vi) complexes (76.3–82.8 kcal mol^–1^),^63^,^64^ another *cis*-dioxoruthenium(vi) complex supported by the Me_3_tacn ligand (87.5 kcal mol^–1^)^65^ and several Mn–oxo complexes (79–84.3 kcal mol^–1^).^66^–^69^

**Table 8:** Hydrogen-atom affinity of selected metal–oxo complexes

Complex	*D*_O–H_	Reference
*cis*-[(bpy)_2_Ru^VI^(O)_2_]^2+^	93.5^*a*^	17
*cis*-[(pdp)Ru^VI^(O)_2_]^2+^	90.8	This work
*cis*-[(mcp)Ru^VI^(O)_2_]^2+^	90.1	This work
[(TSMP)Fe^IV^(O)]	90	59, 60
*cis*-[(Me_3_tacn)Ru^VI^(O)_2_(O_2_CCF_3_)]^2+^	87.5^*a*^	65
[(N4Py)Ru^IV^(O)(OH_2_)]^2+^	84.8	62
[(bpy)_2_(py)Ru^IV^(O)]^2+^	84	61
*trans*-[(N_2_O_2_)Ru^VI^(O)_2_]^2+^	82.8	63
[(TPA)Ru^IV^(O)(OH_2_)]^2+^	82.7	62
[(Me_2_EBC)Mn^IV^(O)(OH)]^+^	84.3	66, 67
Mn^VII^O_4_^–^	80	68
[(phen)_2_Mn^IV^(μ-O)_2_Mn^III^(phen)_2_]^3+^	79	69
*trans*-[(14-TMC)Ru^VI^(O)_2_]^2+^	76.3	64

^*a*^Calculated from the reported electrochemical data.

Another piece of interesting information can be extracted from the pH-dependent cyclic voltammogram of **3c′**, where the Ru^V/III^ couple was observed over the pH range of 1.98 to 7.96. At pH 4.1, for example, the potential of the redox couple *cis*-[(pdp)Ru^V^(O)(OH)]^2+^ + 2e^–^ + 2H^+^ → *cis*-[(pdp)Ru^III^(OH)(OH_2_)]^2+^ occurs at 0.76 V *vs.* SCE. This can provide a basis to estimate the redox potential of the putative *cis*-[(pdp)Fe^V^(O)(OH)]^2+^ or *cis*-[(pdp)Fe^V^(O)_2_]^+^ species, which are perceived to be strong oxidants but have not been reported in the literature. We previously reported a density functional theory (DFT) study of *trans*-dioxo complexes of iron, ruthenium and osmium, *trans*-[(NH_3_)_2_(NMeH_2_)_2_M^VI^(O)_2_]^2+^ (M = Fe, Ru, Os), where the reduction potentials of the corresponding Fe^VI/V^ and Ru^VI/V^ couples were estimated to be 1.3 V and 0.56 V *vs.* NHE, respectively.^70^ This theoretical study implies that a O=Fe=O complex would be ∼0.7 V more oxidizing than the corresponding O=Ru=O complex with the same ligand system. If the same relationship can be applied to Fe/Ru(N_4_) complexes in a *cis*-configuration, the potential of the redox couple *cis*-[(pdp)Fe^V^(O)(OH)]^2+^ + 2e^–^ + 2H^+^ → *cis*-[(pdp)Fe^III^(OH)(OH_2_)]^2+^ (or *cis*-[(pdp)Fe^V^(O)_2_]^+^ + 2e^–^ + 2H^+^ → *cis*-[(pdp)Fe^III^(OH)_2_]^+^, depending on the p*K*_a_ value) would occur at approximately 1.4–1.5 V *vs.* SCE at pH 4.1, which is equivalent to 1.6–1.7 V *vs.* SCE at pH 1. This suggests that a *cis*-dioxoiron(v) species, if it exists, would be much more reactive than the *cis*-dioxoruthenium(vi) counterpart. The highly anodic/oxidizing reduction potential of the *cis*-dioxoiron(v) species may not be favourable for alkene dihydroxylation, as side reactions (*e.g.*, C=C cleavage) may become dominant. In comparison, the Fe^V/III^ couple of *cis*-[(L-N_4_Me_2_)Fe^V^(O)_2_]^+^ (L-N_4_Me_2_ = *N*,*N*′-dimethyl-2,11-diaza[3.3](2,6)pyridinophane), an intermediate proposed to be involved in alkene dihydroxylation,^10^ was computed to occur at 1.34 V *vs.* SCE at pH 1.^71^

### Reactivity of *cis*-dioxoruthenium(vi)

The results presented in this work show the strong oxidizing power of *cis*-dioxoruthenium(vi) complexes containing chiral N_4_ ligands by electrochemical analysis and their reactivity with hydrocarbons. Although several *cis*-dioxoruthenium(vi) complexes are known,14a,15a,16a,^17^ chiral ones have not been reported in the literature to the best of our knowledge. In this work, we isolated and spectroscopically characterized the chiral complex *cis*-[((*R*,*R*)-mcp)Ru^VI^(O)_2_](ClO_4_)_2_ (1e*). Complex 1e* could effect the stoichiometric oxidations of alcohols, alkanes and alkenes, as was found for other *cis*-dioxoruthenium(vi) complexes. In the reaction of 1e* with alkenes, considerable amounts of dihydroxylation products were obtained with moderate enantioselectivities (∼30% ee, Table 2), albeit with the predominant products being C=C bond cleavage ones, such as carbonyl compounds. In addition, a mixture of *syn*- and *anti*-diols was obtained, which possibly indicates the non-concerted nature of the dihydroxylation reaction.^72^,^73^ The reactivity/selectivity in the Ru((*R*,*R*)-mcp)-mediated asymmetric *cis*-dihydroxylation (AD) reaction of alkenes is in great contrast to some of the known, highly efficient chiral Fe(N_4_) or Mn(N_4_) catalysts. For instance, *cis*-[((*R*,*R*)-Me_2_bqcn)Fe^II^(OTf)_2_] and *cis*-[((*S*,*S*)-bqcn)Mn^II^Cl_2_] gave *cis*-diols in up to 95% yields and 99.8% ee *via* proposed *cis*-[((*R*,*R*)-Me_2_bqcn)Fe^III^(OOH)]^2+^ and *cis*-[((*S*,*S*)-bqcn)Mn^V^(O)_2_]^+^ intermediates, respectively.5g,6b

Based on the stoichiometric reaction of *cis*-[(mcp)Ru^VI^(O)_2_]^2+^ (**1e**) with alkenes, a related catalytic reaction was developed using NaIO_4_ as a terminal oxidant. *cis*-[(mcp)Ru^III^(O_2_CCF_3_)_2_]ClO_4_ (**1b**) turned out to be an efficient catalyst for the oxidative scission of aryl alkenes to carbonyl compounds (Table S5, ESI†, 6 examples). At a catalyst loading of 1 mol%, aryl C=C bonds are cleaved to aldehydes or ketones in high conversions (83–100%) and high yields (89–100%).^74^ Over-oxidation of aldehydes to carboxylic acids was not observed by controlling the stoichiometry of NaIO_4_ (10% excess). The timespan of the reaction (1 h) is comparable to that (30 min) reported by Bera and co-workers using an abnormal-NHC–Ru(ii) catalyst.^75^

Using *cis*-[(mcp)Ru^III^(O_2_CCF_3_)_2_]ClO_4_ (**1b**) as a catalyst and H_2_O_2_ as a terminal oxidant, we also developed a catalytic protocol for the oxidation of alcohols (Table S6, ESI†, 14 examples). Alcoholic substrates were effectively oxidized to carbonyl compounds or carboxylic acids in yields up to 98% (see the ESI† for a more detailed description). ESI-MS analysis of a mixture of **1b** and H_2_O_2_ did not reveal formation of **1e** or other high-valent ruthenium–oxo complexes. The active intermediate could be hydroperoxo- or peroxo-Ru(iii) species, which has yet to be clarified.

Reports on the oxidation of alkanes catalysed by ligand-supported ruthenium complexes are sparse in the literature.^76^ In 2010, Du Bois and co-workers developed a RuCl_3_/pyridine/KBrO_3_ protocol for the hydroxylation of various substituted alkane substrates;^77^ In 2012, they improved the yield and allowed a lower catalyst loading by employing [(Me_3_tacn)Ru^III^Cl_3_] as a catalyst in combination with AgClO_4_ as an additive and CAN as a terminal oxidant.^20^ Using desorption electrospray ionization mass spectrometry (DESI-MS), *cis*-[(Me_3_tacn)Ru^VI^(O)_2_(OH)]^+^ was identified to be a plausible reactive hydroxylating agent, but the possible involvement of Ru(v) and/or Ru(iv) species could not be discounted.^78^ In this work, stoichiometric reactions between **1e** and several alkane substrates (Table 3) provided direct evidence that *cis*-dioxoruthenium(vi) preferentially oxidizes the tertiary C–H bonds in hydrocarbons. The same selectivity was observed in catalytic experiments. Aqueous electrochemical and ESI-MS experiments showed that *cis*-[(pdp)Ru^VI^(O)_2_]^2+^ is accessible *via* the successive oxidative deprotonation of a low-valent precursor, such as **3c** or **3c′**. The *D*_O–H_ values of *cis*-[(Me_3_tacn)Ru^VI^(O)_2_(O_2_CCF_3_)]^2+^ and *cis*-[(pdp)Ru^VI^(O)_2_]^2+^ are calculated to be 87.5 and 90.8 kcal mol^–1^, respectively (*vide supra*). We anticipate that the Ru(pdp) complex, with an additional driving force of 3.3 kcal mol^–1^, would be as reactive as the Ru(Me_3_tacn) complex in alkane oxidation reactions. Additionally, the use of a chiral N_4_ supporting ligand might incorporate chirality into the oxygenated products.3c,4d A catalytic system for the oxidation of alkanes by *cis*-[(pdp)Ru^II^(OH_2_)_2_]^2+^ (**3c**) with CAN is herein reported. Compared to the [(Me_3_tacn)Ru^III^Cl_3_]/AgClO_4_/CAN system, our system avoids the use of a Ag^+^ salt as a chloride scavenger, and no pretreatment of the catalyst is required.^79^ In general, 2–5 mol% catalyst (**3c**) and 3–6 equiv. of CAN afforded 3° C–H hydroxylated products in isolated yields of *ca.* 50% (Tables 4 and 5), which are comparable to those in other Ru-catalysed C–H hydroxylation protocols (*e.g.*, [(Me_3_tacn)Ru^III^Cl_3_]/AgClO_4_/CAN and *cis*-[(^*t*^Bu_2_bpy)_2_Ru^II^Cl_2_]/H_5_IO_6_/CF_3_SO_3_H).^20^,^21^ In C–H oxidation with substrates containing a mixture of tertiary and secondary C–H bonds, the reaction occurs preferentially at the tertiary position and is highly stereoretentive (*e.g.*, oxidation of **S1** to **P1** in Table 5, **S11** to **P11** in Table 6), which is a fundamentally defining feature in C–H functionalization rendering this method of synthetic value. When the substrate contains multiple tertiary C–H bonds (**S8–S10**, Table 5), hydroxylation preferentially occurs at the most electron-rich site, as was also observed in other Fe/Mn-catalysed C–H hydroxylation systems (*e.g.*, *cis*-[(pdp)Fe^II^(NCMe)_2_]^2+^/H_2_O_2_/AcOH).3a,b,4b This similar reactivity pattern implies the common electrophilic nature of *cis*-dioxoruthenium(vi) and the active oxidant in Fe(pdp)-catalysed reactions. In literature, the identity of the latter was investigated by multiple research groups which has led to different formulations.5d,^7^,^8^,^9^ Talsi, Bryliakov and co-workers identified an *S* = 1/2 species by EPR and assigned it to [(pdp)Fe^V^(O)(OAc)]^2+^.5*d* Based on computational results, Wang, Que, Shaik and co-workers suggested a cyclic Fe(iii) peracetate complex that undergoes O–O bond cleavage to a transient oxoiron(iv)–AcO˙ species which performs efficient C–H hydroxylations.^7^

We also demonstrated the strong oxidizing power of this catalytic system in the reaction with propane and ethane (Table 7). Although the turnover numbers are not impressive, identification of appreciable amounts of the various oxidation products is significant, as light alkanes often exhibit resistance to oxidation. To the best of our knowledge, this represents a rare example of ruthenium-catalysed/mediated oxidation of light alkanes (<C4), except Drago's reported work on the *cis*-[(dmp)Ru^II^(S)_2_]^2+^ (S = MeCN or H_2_O)-catalysed hydroxylation of methane with H_2_O_2_.^80^

Some issues remain to be resolved/explored that are worth being addressed. First, the stability/robustness of the highly oxidizing *cis*-dioxoruthenium(vi) species is of concern. In CAN-driven catalytic oxidation of alkanes, the turnover number based on **3c** is typically less than 30. Post-reaction analysis of the mixture revealed that the catalyst had degraded/decomposed almost completely. A likely deactivation pathway of the catalyst is the oxidation of the ligand by the strongly oxidizing Ru–oxo intermediate. Indeed, it was noted that complexes **4c** and **6c** showed much poorer activities than **3c** and **5c** (Tables 4 and S4, ESI†), presumably due to the intramolecular oxidation of the *ortho*-Me group by the Ru–oxo moiety.^50^ Although our recent work on the Fe(N_4_)-catalysed AD reaction showed that installation of an *ortho*-Me group could substantially improve the catalyst activity (particularly the enantioselectivity),5*g* this strategy cannot be directly transplanted to the ruthenium chemistry. For the Fe((*R*,*R*)-Me_2_bqcn)-catalysed AD reaction, the active intermediate was proposed to be [((*R*,*R*)-Me_2_bqcn)Fe^III^(OOH)]^2+^ rather than dioxoiron(v).5*g* From ESI-MS experiments, it was also demonstrated that the decomposition of Ru(pdp) complexes under oxidizing condition is considerably fast when [Ru] ≥ 1 mM. Thus, a delicate balance between the oxidizing power and stability of the active intermediate is yet to be achieved for efficient ruthenium-catalysed C–H oxidation. Moreover, either stoichiometrically or catalytically, the studied chiral ruthenium complexes (1e*, 3c–6c*) did not show noticeable enantioselectivity in reactions with racemic tertiary alkane substrates (*e.g.*, entries 9, 10, Table 3; entry 6, Table 5). This suggests, without any directing group,3*c* there is not sufficient chiral differentiation between the two isomeric forms by kinetic resolution at the chiral ruthenium centre.

## Conclusions

In this work, we reported the preparation and electrochemistry of several ruthenium complexes bearing tetradentate N_4_ ligands including *cis*-[(mcp)Ru^III^(O_2_CCF_3_)_2_]ClO_4_ (**1b**) and *cis*-[(pdp)Ru^III^(O_3_SCF_3_)_2_]CF_3_SO_3_ (**3c′**). Complex *cis*-[(mcp)Ru^VI^(O)_2_](ClO_4_)_2_ (**1e**) was obtained from CAN oxidation of **1b** in aqueous solution. Complex **1e** is a powerful oxidant with *E*(Ru^VI/V^) = 0.78 V (*vs.* Ag/AgNO_3_) in acetonitrile or *E*° = 1.11 V (*vs.* SCE) at pH 1. In aqueous *tert*-butanol, [((*R*,*R*)-mcp)Ru^VI^(O)_2_](ClO_4_)_2_ (1e*) underwent stoichiometric alkene *cis*-dihydroxylation to afford *cis*-diol in 24% ee for *trans*-β-methylstyrene oxidation. With high hydrogen-atom affinities (*D*_O–H_ = 90.1–90.8 kcal mol^–1^), **1e** and chemically generated *cis*-[(pdp)Ru^VI^(O)_2_]^2+^ are active oxidants for C–H oxidation. *cis*-[(pdp)Ru^II^(OH_2_)_2_]^2+^ (**3c**), in combination with CAN as a terminal oxidant, catalysed the oxidation of unactivated C–H bonds including those of some pharmaceutical ingredients and natural product derivatives. This work demonstrates that efficient oxidation catalysts can be constructed based on the *cis*-dioxoruthenium(vi) moiety on a N_4_ ligand platform. The diversity and flexibility of chiral N_4_ ligand design will direct subsequent efforts to improve the reaction selectivities.4*d* Further studies are also directed to gain a better understanding of the reaction mechanism in hydrocarbon oxidations and to explore other catalytic activities of chiral Ru(N_4_) complexes.

## Conflicts of interest

There are no conflicts to declare.

## Supplementary Material

Supplementary information

Crystal structure data
