# Supplementary material for: cis-Oxoruthenium complexes supported by chiral tetradentate amine (N4) ligands for hydrocarbon oxidations
Source: Chem Sci. 2018 Feb 15;9(10):2803–16. doi: 10.1039/c7sc05224c (PMC5943683; doi:10.1039/c7sc05224c)
Supplement: Supplementary file 1 [file SC-009-C7SC05224C-s001.pdf]

Electronic Supplementary Material (ESI) for Chemical Science.
This journal is © The Royal Society of Chemistry 2018

Electronic Supplementary Information

cis-Oxoruthenium complexes supported by chiral tetradentate amine (N_4) ligands for hydrocarbon oxidations

Chun-Wai Tse,[ab] Yungen Liu,[ac] Toby Wai-Shan Chow,[a] Chaoqun Ma,[c] Wing-Ping Yip,[a] Xiao-Yong Chang,[a] Kam-Hung Low,[a] Jie-Sheng Huang,[a] and Chi-Ming Che*[abc]

[a.] State Key Laboratory of Synthetic Chemistry and Department of Chemistry, The University of Hong Kong, Pokfulam Road, Hong Kong, China. E-mail: cmche@hku.hk

[b.] HKU Shenzhen Institute of Research and Innovation, Shenzhen, Guangdong 518053, China

[c.] Department of Chemistry, Southern University of Science of Technology, Shenzhen, Guangdong 518055, China

Table of Contents

Experimental Section ······ S3
Materials ······ S3
Instrumentation ······ S3
Synthesis of ruthenium complexes ······ S4
Procedure for stoichiometric organic oxidations ······ S9
Procedure for catalytic studies ······ S9
Scheme S1 Preparation of **3c′** ······ S11
Table S1 Selected bond lengths (Å) and angles (°) for **1a**, **2a**, and **3a·ClO$_4$** ······ S11
Table S2 Crystallographic data of **1a** and **2a** ······ S12
Table S3 Crystallographic data of **3a**·ClO$_4$, **5d** and **6d** ······ S13
Table S4 Oxidation of racemic 2-phenylhexane with CAN catalyzed by *cis*-$[(N_4)Ru^{II}(OH_2)_2]^{2+}$ complexes ······ S14
Table S5 Catalytic alkene oxidations by **1b** in aqueous *tert*-butanol with $NaIO_4$ ······ S15
Table S6 Catalytic alcohol oxidations by **1b** in aqueous *tert*-butanol with H_2O_2 ······ S16
Fig. S1 ORTEP drawing of the cation of **1a** ······ S18
Fig. S2 ORTEP drawing of the cation of **2a** (**I**) ······ S18
Fig. S3 ORTEP drawing of the cation of **2a** (**II**) ······ S19
Fig. S4 ORTEP drawing of the cation of **3a·ClO$_4$** ······ S19
Fig. S5 ORTEP drawing of the cation of **6d** ······ S19
Fig. S6 UV-Vis absorption spectra of **1a**, **2a**, **3a** and **4a** in acetonitrile ······ S20
Fig. S7 UV-Vis absorption spectra of **3c**, **4c**, **5c** and **6c** in water ······ S20
Fig. S8 Cyclic voltammograms of **1a** and **2a** in DMF ······ S20
Fig. S9 Cyclic voltammograms of **1a**, **2a**, **3a** and **4a** in acetonitrile ······ S21
Fig. S10 Cyclic voltammograms of **2a** in acetonitrile at different scan rates ······ S21
Fig. S11 Cyclic voltammograms of **5d** and **6d** in acetonitrile ······ S22
Fig. S12 Cyclic voltammogram of **1b** in acetate buffer (pH 5) ······ S22
Fig. S13 Cyclic voltammograms of **3c′** at pH 1 in the presence of organic substrates ······ S23
Fig. S14 Cyclic voltammogram in 0.1 M CF_3SO_3H (pH 1) of **3c·OTs**, **5c·OTs** and **6c·OTs** ······ S24
Fig. S15 Cyclic voltammograms of **3c′** in Britton-Robinson buffer ······ S25
Fig. S16 ESI-MS signals of **1e** in water ······ S26
Fig. S17 ESI-MS signals for a reaction mixture of **3c·CF$_3$SO$_3$** and 4 equiv. of $Ce^{IV}(ClO_4)_4$ in water ······ S26
Fig. S18 Time trace from of the signal intensity of *m/z* = 555.04 ion ······ S27
Fig. S19 ESI-MS signals for a reaction mixture of **3c·CF$_3$SO$_3$** and 6 equiv. of $Ce^{IV}(ClO_4)_4$ in water and time trace from 0 to 3 min of the signal intensity of *m/z* = 456.10 ion ······ S27
Fig. S20 ESI-MS signals for a reaction mixture of **4c·OTs** with CAN ······ S28
NMR spectra of oxidation products **P11–P14** ······ S29
Notes and references ······ S33

Experimental Section

Materials

Solvents were purified according to reported methods.[1] Ligands *N*,*N*′-dimethyl-*N*,*N*′-bis(pyridin-2-ylmethyl)cyclohexane-1,2-diamine (mcp),[2] *N*,*N*′-dimethyl-*N*,*N*′-bis((6-methylpyridin-2-yl)methyl)cyclohexane-1,2-diamine (Me_2mcp),[3] 1,1'-bis(pyridin-2-ylmethyl)-2,2'-bipyrrolidine (pdp),[4] 1,1'-bis((6-methylpyridin-2-yl)methyl)-2,2'-bipyrrolidine (Me_2pdp),[5] *N*,*N*′-dimethyl-*N*,*N*′-di(quinolin-8-yl)cyclohexane-1,2-diamine (bqcn),[6] and *N*,*N*′-dimethyl-*N*,*N*′-bis(2-methylquinolin-8-yl)cyclohexane-1,2-diamine (Me_2bqcn),[7] and ruthenium complex $[Ru^{II}(OH_2)_6](OTs)_2$ were prepared as reported.[8,9] Silver trifluoro-methanesulfonate (99+%, Acros) was used as received. Cerium(IV) ammonium nitrate (CAN) was purchased from Acros Organics; its purity was determined by iodometric titration to be > 98%. $Ce^{IV}(ClO_4)_4$ (~ 0.5 N in perchloric acid) was obtained from Aldrich; its $[Ce^{IV}]$ and $[H^+]$ were determined to be 0.485 M and 7 M, respectively, by iodometric and acid-base titrations. Organic substrates were obtained from commercial sources and used as received, except that **S3**,[10] **S5** (racemic),[11] **S6**,[11] **S7**,[11] and **S8**[11] were prepared using published protocols.

Instrumentation

NMR spectra were obtained using Bruker DPX-300, Advance 400, 500, or 600 FT-NMR spectrometers. Chemical shifts in ppm were referenced to residual non-deuterated solvents. Infrared spectra were recorded as a KBr disc or Nujol mull on a Nicolet 20 SXC FT-IR spectrophotometer. UV-visible absorption spectra were recorded on a Hewlett-Packard 8453A diode array spectrophotometer. Fast atom bombardment mass spectrometry (FAB-MS) was performed with a Thermo Scientific DFS high resolution spectrometer or a Finnigan MAT 95 mass spectrometer. Electrospray ionization mass spectrometry (ESI-MS) was performed with a Finnigan MAT LCQ spectrometer or a Waters Micromass Q-Tof Premier quadrupole time-of-flight tandem mass spectrometer (Waters Corporation, Milford, USA). Elemental analyses were performed at the Institute of Chemistry of the Chinese Academy of Sciences. Gas chromatography-mass spectrometry (GC-MS) analyses were performed with an Agilent Technologies 7890B Network GC System equipped

with an Agilent Technologies 5977A Network Mass Selective Detector (MSD). An Agilent Technologies HP-5MS Ultra Inert (30.0 m × 0.25 mm, 0.25 micron) capillary column was used to analyze the reaction mixture in oxidation reactions. Helium was used as carrier gas. Chlorobenzene or 1,4-dichlorobenzene, depending on the molecular weight/retention time of oxidized products, was used as internal standard for quantification. The X-ray diffraction data of single crystals were collected on MAR, Bruker D8 VENTURE Dual Source Photon100 CMOS, or Bruker APEX-II CCD diffractometers.

Synthesis of ruthenium complexes

***cis*-[(mcp)Ru^{III}Cl$_2$]ClO$_4$ (1a)**. An ethanolic solution of mcp (0.5 g, in 250 mL) was added dropwise over 3 h to a stirred ethanolic suspension (250 mL) of $K_2[RuCl_5(OH_2)]$ (0.5 g) under refluxing condition. Upon complete addition of ligand, the mixture was further refluxed for 15 h. The reaction mixture was then cooled, with addition of a few drops of concentrated HCl, filtered, and evaporated to dryness. The residue was dissolved in water (5 mL), followed by addition of saturated $NaClO_4$ solution (10 mL). The crude product was recrystallized from hot HCl (0.1 M, 100 mL) to afford **1a** as a red crystalline solid. Yield: 55%. Anal. Calcd. for $C_{20}H_{28}N_4O_4Cl_3Ru$: C, 40.31; H, 4.74; N, 9.40. Found: C, 40.34; H, 4.76; N, 9.43. FAB-MS: *m*/*z* 496.1 (M^+). UV-Vis (MeCN) λ_{max}/nm (ε_{max}/dm^{3}mol^{-1} cm^{-1}): 415 (1920), 299 (7440).

***cis*-[(mcp)Ru^{III}(O$_2$CCF$_3$)$_2$]ClO$_4$ (1b)**. A mixture of [(mcp)Ru^{III}Cl$_2$]ClO$_4$ (**1a**, 0.3 g) and zinc amalgam (1 g) in distilled water (30 mL) was heated at 80 °C for 30 min, giving a dark green solution. Zinc amalgam was removed, and the remaining solution was treated with CF_3CO_2H (0.2 M, 20 mL) and silver trifluoromethanesulfonate (0.3 g). Insoluble AgCl was removed by filtration, and the volume was slowly reduced to *ca*. 15 mL by heating at 80 °C to give a yellow solution. Addition of saturated $NaClO_4$ solution (5 mL) induced precipitation of **1b** as a pale yellow solid. The crude product was recrystallized in aqueous CF_3CO_2H (0.1 M, 20 mL). Yield: 20%. Anal. Calcd. for $C_{24}H_{28}F_6O_8N_4ClRu$: C, 38.38; H, 3.76; N, 7.46. Found: C, 38.53; H, 3.84; N, 7.53. IR (KBr): 1712, 1613 and 1393 cm^{-1}. FAB-MS: *m/z* 652.1 (M^+). UV-Vis (MeCN) λ_{max}/nm (ε_{max}/dm^{3}mol^{-1}cm^{-1}): 269 (7040).

***cis*-[(mcp)Ru^{II}(NCMe)$_2$](ClO$_4$)$_2$** (**1d**). This complex was obtained as a by-product in the stoichiometric oxidation of hydrocarbons by *cis*-[(mcp)Ru^{VI}(O)$_2$](ClO$_4$)$_2$ (**1e**), after diethyl ether diffusion into an acetonitrile solution. Anal. Calcd. for $C_{24}H_{34}N_6O_8Cl_2Ru$: C 40.80; H, 4.85; N 11.89. Found: C 40.31; H, 4.74; N 11.96. UV-Vis (MeCN) λ_{max}/nm (ε_{max}/dm^3 mol^{-1}cm^{-1}): 245 (9900), 347 (9750). FAB-MS: *m/z* 607.2 ([M + ClO$_4$]$^+$). ^{1}H NMR (300 MHz, CD$_3$CN): δ 0.93–1.02 (*m*, 2H), 1.12–1.26 (*m*, 2H), 1.60–1.62 (*m*, 2H), 2.06–2.16 (*m*, 2H), 2.21–2.31 (*m*, 2H), 2.32 (*s*, 6H), 2.45 (*s*, 6H), 4.24 (*d*, 2H, *J* = 16.0 Hz), 4.50 (*d*, 2H, *J* = 16.0 Hz), 7.50 (*t*, 2H, *J* = 6.6 Hz), 7.59 (*d*, 2H, *J* = 7.8 Hz), 7.93 (*t*, 2H, *J* = 7.7 Hz), 8.94 (*d*, 2H, *J* = 5.6 Hz).

***cis*-[(mcp)Ru^{VI}(O)$_2$](ClO$_4$)$_2$** (**1e**). To an ice-cooled solution of **1b** (0.1 g) in H_2O (10 mL) was added a solution of $(NH_4)_2[Ce^{IV}(NO_3)_6]$ (1 g, in 2 mL water). The solution gradually turned pale green. Upon addition of a saturated solution of $NaClO_4$ (4 mL), a pale green solid was formed. The solid was collected on a frit and air-dried. Yield: 66%. UV-Vis (MeCN) λ_{max}/nm (ε_{max}/dm^3 mol^{-1}cm^{-1}): 261 (8700), 344 (2210). FAB-MS: *m/z* 458.1 (M$^+$). ^{1}H NMR (300 MHz, CD$_3$CN): δ_H 0.91–0.96 (*m*, 2H), 1.14–1.22 (*m*, 2H), 1.51–1.62 (*m*, 2H), 1.90–1.98 (*m*, 2H), 2.05–2.15 (*m*, 2H), 2.20 (*s*, 6H), 3.87 (*d*, 2H, *J* = 16 Hz), 4.44 (*d*, 2H, *J* = 16 Hz), 7.40 (*t*, 2H, *J* = 6.4 Hz), 7.53 (*d*, 2H, *J* = 7.4 Hz), 7.84 (*t*, 2H, *J* = 7.7 Hz), 8.79 (*d*, 2H, *J* = 5.6 Hz).

***cis*-[(Me$_2$mcp)Ru^{III}Cl$_2$]ClO$_4$ (2a)**. The procedure was similar to that for the preparation of **1a**, except that 6-Me$_2$mcp, instead of mcp, was employed as ligand. Recrystallization of the crude product by slow diffusion of diethyl ether into an acetonitrile solution afforded **2a** as an orange-red crystalline solid. Yield: 32%. Anal. Calcd. for $C_{22}H_{32}N_4O_4Cl_3Ru$: C, 42.35; H, 5.17; N, 8.98. Found: C, 42.32; H, 5.37; N, 8.71. FAB-MS: *m/z* 524.1 (M$^+$). UV-Vis (MeCN) λ_{max}/nm (ε_{max}/dm^{3}mol^{-1} cm^{-1}): 430 (1850), 277 (7785).

***cis*-[(pdp)Ru^{III}Cl$_2$]Cl (3a·Cl).** An ethanolic solution (200 mL) of pdp (0.523 g) was added dropwise over 3 h to a stirred ethanolic suspension (250 mL) of $K_2[RuCl_5(OH_2)]$ (0.761 g) under refluxing condition. Upon complete addition of ligand, the mixture was further refluxed for 15 h. The reaction mixture was then cooled, with addition of 5 drops of concentrated HCl, filtered, and evaporated to dryness. The brown oil thus

obtained was re-dissolved in methanol followed by addition of excess diethyl ether. Complex **3a·Cl** was isolated as a brown hygroscopic solid. Yield: 78%. Anal. Calcd. (%) for $C_{20}H_{26}N_4RuCl_3 \cdot 3HCl$: C 37.58, H 4.57, N 8.76; found: C 37.29, H 4.53, N 8.42. ESI-MS (H_2O): *m/z* 494.1 (M^+). UV-Vis (MeCN) λ_{max}/nm (ε_{max}/dm^{3}mol^{-1} cm^{-1}): 301 (7220), 404 (2070).

***cis*-[(pdp)Ru^{III}Cl$_2$]ClO$_4$ (3a·ClO$_4$).** This complex was prepared by recrystallizing **3a·Cl** (0.6 g) by vapor diffusion of diethyl ether into its acetonitrile solution in the presence of 0.1 M $LiClO_4$. The product was obtained as a yellow crystalline solid including a diffraction-quality crystal suitable for X-ray crystallography. Yield: 82%. Anal. Calcd. (%) for $C_{20}H_{26}N_4RuCl_3O_4$: C 40.45, H 4.41, N 9.43; found: C 40.20, H 4.41, N 9.45. ESI-MS (MeCN): *m/z* 494.1 (M^+). UV-Vis (MeCN) λ_{max}/nm (ε_{max}/dm^{3}mol^{-1} cm^{-1}): identical to that of **3a·Cl**.

***cis*-[(pdp)Ru^{II}(OH$_2$)$_2$](OTs)$_2$ (3c·OTs).** The pdp ligand (129 mg) was dissolved in distilled THF (40 mL). [Ru^{II}(OH$_2$)$_6$](OTs)$_2$ (265 mg) was then added under positive pressure of argon. The resultant mixture was heated under argon for 1 h. After cooling and evacuation of solvent under vacuum, the crude product was dissolved in dichloromethane, filtered to remove excess [Ru^{II}(OH$_2$)$_6$](OTs)$_2$, and vacuum-dried. The product thus obtained was generally pure analytically. In rare cases where ESI-MS analysis showed the presence of unreacted ligand (detected as *m/z* LH$^+$), the product was further purified by washing its aqueous solution (40 mL) with chloroform (40 mL × 3) using a separating funnel. Complex **3c·OTs** was obtained as a green micro-crystalline solid upon evaporation of the aqueous solution under vacuum. Yield: 68%. Anal. Calcd. (%) for $C_{34}H_{44}N_4RuS_2O_8$: C 50.92, H 5.53, N 6.99; found: C 51.24, H 5.69, N 6.81. ESI-MS (H_2O): *m/z* 595.1 ([(pdp)Ru^{II}(OTs)]$^+$). UV-Vis (H_2O) λ_{max}/nm (ε_{max}/dm^{3}mol^{-1}cm^{-1}): 249 (10043), 394 (6880). UV-Vis (MeCN) λ_{max}/nm (ε_{max}/dm^{3}mol^{-1}cm^{-1}): 248 (11560), 362 (6730). UV-Vis (CH_2Cl_2) λ_{max}/nm (ε_{max}/dm^{3}mol^{-1}cm^{-1}): 251 (9930), 414 (7270). ^{1}H NMR (300 MHz, CD_3CN): δ 1.31–1.37 (m, 2H), 1.80–1.84 (m, 2H), 2.10–2.19 (m, 4H), 2.42 (s, 6H), 2.43–2.48 (m, 2H), 2.64–2.65 (m, 2H), 3.20–3.28 (m, 2H), 4.07–4.12 (d, 2H, *J* = 15.4 Hz), 4.28–4.33 (d, 2H, *J* = 15.5 Hz), 7.48–7.54 (m, 4H), 7.88–7.94 (dt, 2H, *J* = 7.8 Hz), , 8.99–9.01 (d, 2H, *J* = 5.4 Hz).[12]

***cis*-[(pdp)Ru^{II}(OH$_2$)$_2$](CF$_3$SO$_3$)$_2$ (3c·CF$_3$SO$_3$)**. A mixture of **3a·Cl** (0.25 g) and zinc amalgam (0.8 g) in distilled water (23.5 mL) was heated at 80 °C for 30 min under argon to give a dark green solution. The reaction mixture was cooled to room temperature, filtered under argon, and treated with silver trifluoromethanesulfonate (0.363 g). After heating at 80 °C for another 30 min under argon, the reaction mixture was cooled to room temperature and filtered under argon to remove AgCl. After removal of solvents under vacuum followed by exhaustive vacuum-drying, the product was isolated as a highly hygroscopic dark-green solid. Yield: 88%. ESI-MS (H_2O): *m/z* 573.1 ([(pdp)Ru^{II}(O_3SCF_3)])$^+$. UV-Vis (H_2O) λ_{max}/nm (ε_{max}/dm^{3}mol^{-1}cm^{-1}): 249 (9600), 395 (6850). Cautious note: This complex was not fully characterized and may contain Zn(CF_3SO_3)$_2$ as an impurity.

***cis*-[(pdp)Ru^{III}(O$_3$SCF$_3$)$_2$]CF$_3$SO$_3$ (3c′)**. Following the procedure for the synthesis of **3c·CF$_3$SO$_3$**, after Zn reduction and dechlorination by Ag^+, the reaction mixture was exposed to air and cooled to room temperature. Insoluble AgCl was removed by filtration, and the solvents were evaporated under vacuum. The resulting green oil was washed thoroughly with diethyl ether and dried under vacuum for 24 h to produce **3c′** as a highly hygroscopic green solid. Yield: 83%. Anal. Calcd. (%) for $C_{23}H_{26}F_9N_4O_9RuS_3\cdot5H_2O$: C 28.75, H 3.78, N 5.83; found: C 28.70, H 3.83, N 5.71. ESI-MS (H_2O): *m/z* 458.1 ([(pdp)Ru^{III}(OH)$_2$])$^+$. UV-Vis (H_2O) λ_{max}/nm (ε_{max}/dm^{3}mol^{-1}cm^{-1}): 246 (9190), 296 (5400).

***cis*-[(Me$_2$pdp)Ru^{III}Cl$_2$]Cl (4a·Cl).** This complex was prepared in a manner similar to that of **3a·Cl** from Me$_2$pdp (0.42 g) and $K_2[RuCl_5(OH_2)]$ (0.54 g). The product was isolated as a brown solid. Yield: 97%. Anal. Calcd. (%) for $C_{22}H_{30}N_4RuCl_3\cdot H_2O$: C 45.88, H 5.60, N 9.73; found: C 45.44, H 5.80, N 9.26. ESI-MS (MeOH or H_2O): *m/z* 522 (M^+). UV-Vis (MeCN) λ_{max}/nm (ε_{max}/dm^{3}mol^{-1} cm^{-1}): 258 (8320), 383 (3000).

***cis*-[(Me$_2$pdp)Ru^{II}(OH$_2$)$_2$](OTs)$_2$ (4c·OTs).** This complex was prepared in a manner similar to that of **3c·OTs** except that Me$_2$pdp (70 mg), instead of pdp, was used as ligand. Complex **4c·OTs** was obtained as a brown-green solid. Yield: 76%. Anal. Calcd. (%) for $C_{36}H_{48}N_4RuS_2O_8$: C 52.10, H 5.83, N 6.75; found: C 51.96, H 6.03, N 6.74. ESI-MS (H_2O): *m/z* 623.2 ([(Me$_2$pdp)Ru^{II}(OTs)]$^+$). UV-Vis (H_2O) λ_{max}/nm

(ε_{max}/dm^{3}mol^{-1}cm^{-1}): 258 (12960), 361 (5490). UV-Vis (MeCN) λ_{max}/nm (ε_{max}/dm^{3}mol^{-1}cm^{-1}): 254 (13500), 336 (6920). ^{1}H NMR (400 MHz, CD_3CN): δ 1.36–1.39 (m, 2H), 1.77–1.79 (m, 2H), 2.09–2.11 (m, 4H), 2.40–2.42 (m, 2H), 2.66–2.70 (m, 2H), 2.75 (s, 6H), 3.45–3.50 (m, 2H), 4.36 (s, 4H), 7.33–7.38 (m, 4H), 7.72–7.79 (m, 2H).[12]

***cis*-[(bqcn)Ru^{II}(OH$_2$)$_2$](OTs)$_2$ (5c·OTs).** This complex was prepared in a manner similar to that of **3c·OTs** except that bqcn (158 mg, 0.4 mmol), instead of pdp, was used as ligand. The reaction was conducted at room temperature and the reaction time was extended to 18 h. Complex **5c·OTs** was obtained as a red microcrystalline solid. Yield: 71%. Anal. Calcd. (%) for $C_{40}H_{46}N_4O_8RuS_2\cdot H_2O$: C 53.74, H 5.41, N 6.27; found: C 53.65, H 5.29, N 5.90. ESI-MS (H_2O): *m/z* 669.1 ([(bqcn)Ru^{II}(OTs)]$^+$). UV-Vis (H_2O) λ_{max}/nm (ε_{max}/dm^{3}mol^{-1}cm^{-1}): 287 (11220), 477 (6880).

***cis*-[(bqcn)Ru^{II}(NCMe)$_2$](ClO$_4$)$_2$ (5d).** This complex was prepared by successive vapor diffusion of diethyl ether into an acetonitrile solution of **5c·OTs** in the presence of 0.1 M $LiClO_4$. The product **5d** was obtained as a yellow crystalline solid. Yield: 70%. Anal. Calcd. (%) for $C_{30}H_{34}N_6RuCl_2O_8Cl_2\cdot CH_3CN$: C 46.89, H 4.55, N 8.65; found: C 47.01, H 4.55, N 8.77. ESI-MS (MeCN): *m/z* 290.1 ([M]$^{2+}$). UV-Vis (MeCN) λ_{max}/nm (ε_{max}/dm^{3}mol^{-1}cm^{-1}): 278 (12100), 415 (6900). ^{1}H NMR (400 MHz, CD_3CN): this compound exists as a mixture of *cis*-α and *cis*-β isomers which gives highly complicated NMR signals, see in-text discussion.

***cis*-[(Me$_2$bqcn)Ru^{II}(OH$_2$)$_2$](OTs)$_2$ (6c·OTs).** This complex was prepared in a manner similar to that of **3c·OTs** except that Me$_2$bqcn (170 mg), instead of pdp, was used as ligand. The reaction was conducted at room temperature and the reaction time was extended to 18 h. Complex **6c·OTs** was obtained as a reddish-brown solid. Yield: 41%. Anal. Calcd. (%) for $C_{42}H_{50}N_4RuS_2O_8$: C 55.80, H 5.57, N 6.20; found: C 55.33, H 5.70, N 5.90. ESI-MS (H_2O): *m/z* 697.1 ([(Me$_2$bqcn)Ru^{II}(OTs)]$^+$). UV-Vis (H_2O) λ_{max}/nm (ε_{max}/dm^{3}mol^{-1}cm^{-1}): 287 (13680), 453 (4690).

***cis*-[(Me$_2$bqcn)Ru^{II}(NCMe)$_2$](ClO$_4$)$_2$ (6d).** This complex was prepared by successive vapor diffusion of diethyl ether into an acetonitrile solution of **6c·OTs** in the presence

of 0.1 M $LiClO_4$. The product **6d** was obtained as a yellow crystalline solid. Yield: 70%. Anal. Calcd. (%) for $C_{32}H_{38}N_6RuCl_2O_8Cl_2$: C 47.65, H 4.75, N 10.42; found: C 47.86, H 4.80, N 10.36. ESI-MS (MeCN): *m/z* 304.1 ($[M]^{2+}$). UV-Vis (MeCN) λ_{max}/nm (ε_{max}/$dm^3mol^{-1}cm^{-1}$): 272 (14685), 402 (5500). ^1H NMR (400 MHz, CD_3CN): δ 0.77–0.82 (m, 2H), 1.50–1.53 (m, 2H), 1.58–1.60 (m, 2H), 1.96–2.00 (m, 2H), 2.85–2.88 (m, 2H), 3.07 (s, 6H), 3.50 (s, 6H), 7.57–7.58 (d, 2H, *J* = 8.5 Hz), 7.66–7.70 (t, 2H, *J* = 8.0 Hz), 7.93–7.95 (d, 2H, *J* = 8.1 Hz), 8.0–8.02 (d, 2H, *J* = 7.7 Hz), 8.34–8.36 (d, 2H, *J* = 8.5 Hz).

Stoichiometric organic oxidations by 1e

To a 100-mL Schlenck flask was added alkene (30 mmol), *tert*-butanol (10 mL) and distilled water (2 mL). The mixture was degassed by three freeze-pump-thaw cycles and filled with argon. The *cis*-dioxoruthenium complex **1e** (300 μmol) was added under a positive pressure of argon, and the reaction mixture was stirred magnetically at room temperature for 30 min. To work-up, the organic products were separated from the reaction mixture by diethyl ether extraction (3 × 50 mL). After washing with brine (2 × 10 mL) and drying over $MgSO_4$, the volume of the ethereal extract was reduced to about 3 mL for GC analysis and/or column chromatographic purification. Similar experimental procedures were employed when acetonitrile was used as solvent for the alkene oxidation. The organic products (e.g. *cis*/*trans*-diols, carbonyl compounds) were identified by ^1H and ^{13}C NMR spectroscopy by comparing with authentic or literature data. Enantiomeric excess of the diol products was determined by chiral HPLC.

Similar procedures were employed in the oxidation of alcohols and alkanes (Table 3 in main text), whereas acetonitrile was used as solvent.

Catalytic studies

Catalytic alkene oxidation by 1b

To a mixture of *tert*-butanol (2 mL) and distilled water (1 mL) containing substrate (0.5 mmol) was added catalyst **1b** (1 mol%). Then, $NaIO_4$ (1.1 mmol, 2.2 equiv.) was added to the reaction mixture all at once. The reaction mixture was reacted at room temperature for 1 h. To work-up, any unreacted oxidant was quenched by saturated

Na_2SO_3 solution (2 mL), followed by extraction with ethyl acetate (3 × 10 mL). The combined organic extracts were dried over $MgSO_4$, and subjected to GC analysis and/or column chromatographic purification. The organic products were identified by GC by comparing with authentic sample and/or NMR spectroscopy of the isolated product.

Catalytic alcohol oxidation by 1b

In a 25-mL flask, alcohol (1 mmol) was dissolved in aqueous *tert*-butanol (3 mL, *tert*-butanol/water = 2:1 v/v), followed by addition of **1b** (7.5 mg, 10 μmol) into the solution. The reaction mixture was then heated to reflux. Under refluxing condition, H_2O_2 solution (0.35 mL, 4 equiv.) was added via syringe pump over 8 h. Upon complete H_2O_2 addition, the mixture was further refluxed for 4 h. To work-up, any unreacted H_2O_2 was quenched by saturated $NaHSO_3$ solution (2 mL), followed by extraction with diethyl ether (5 × 20 mL). The combined ethereal extracts were dried over $MgSO_4$, and subjected to GC analysis and/or column chromatographic purification. The organic products were identified by GC by comparing with authentic sample and/or NMR spectroscopy of the isolated product.

Catalytic alkane oxidation by *cis*-$[(N_4)Ru^{II}(OH_2)_2]^{2+}$ complexes

To a mixture of substrate (0.2–0.25 mmol) and Ru catalyst (2–5 mol%) in *tert*-butanol (2 mL) and distilled water (2 mL) was added CAN (3 equiv.). In some entries depicted in the Tables, after the intense orange-red color of CAN had disappeared (in 10 min to 1 h), another portion of CAN (3 equiv.) was added to the system. The reaction mixture was stirred at room temperature for the time specified in each entry. To work-up, the mixture was treated with saturated Na_2SO_3 solution (2 mL), followed by extraction with ethyl acetate (3 × 10 mL). Organic products were identified and quantified by GC-MS (internal standard = 1,4-dichlorobenzene) by comparing with authentic samples, or by NMR spectroscopy after purification/isolation by chromatography on silica gel.

Scheme S1 Preparation of **3c′**.

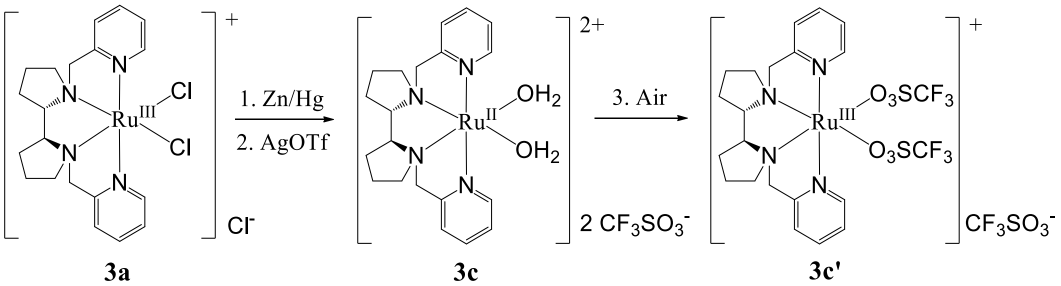

Table S1 Selected bond lengths (Å) and angles (°) for **1a**, **2a**, and **3a·ClO$_4$**.

	**1a**	**2a (I)**	**2a (II)**	**3a·ClO$_4$**
Ru–N$_1$	2.070(2)	2.125(3)	2.161(3)	2.090(2)
Ru–N$_4$	2.079(2)	2.125(3)	2.161(3)	2.087(2)
Ru–N$_2$	2.112(2)	2.142(6)	2.137(6)	2.108(2)
Ru–N$_3$	2.117(2)	2.142(6)	2.137(6)	2.113(2)
Ru–Cl$_1$	2.3373(8)	2.3571(16)	2.3445(16)	2.3499(8)
Ru–Cl$_2$	2.3331(8)	2.3571(16)	2.3445(16)	2.3378(7)
α	92.67(3)	95.25(8)	93.52(9)	95.64(3)
β	83.85(9)	83.5(3)	82.2(3)	82.59(9)

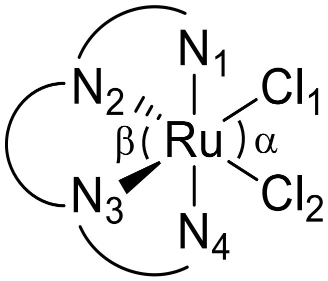

Table S2 Crystallographic data of **1a** and **2a**.

	**1a**	**2a**
Empirical Formula	$C_{20}H_{28}Cl_3N_4O_4Ru$	$C_{22}H_{32}Cl_3N_4O_4Ru$
Formula weight	595.88	623.94
Temperature/K	200	253
Crystal system	Monoclinic	Monoclinic
Space group	P 2_1/c	P 2
a (Å)	11.0085(6)	10.505(2)
b (Å)	13.2457(7)	10.579(2)
c (Å)	15.8552(9)	11.649(2)
α (°)	90	90
β (°)	92.636(2)	101.09(3)
γ (°)	90	90
V (Å^3)	2309.5(2)	1270.4(4)
Z	4	2
Diffractometer	Bruker D9 Venture	MAR
D_c (g cm^{-3})	1.714	1.631
μ (mm^{-1})	1.06	0.969
F(000)	1212	638
Crystal size (mm^3)	0.35 × 0.23 × 0.21	0.6 × 0.25 × 0.15
Radiation	MoKα (λ = 0.71073)	MoKα (λ = 0.71073)
2Θ range for data collection/°	4.8 to 50.0	0 to 50.94
Index ranges	$-12 \le h \le 13$, $-15 \le k \le 15$, $-18 \le l \le 18$	$-12 \le h \le 12$, $-12 \le k \le 12$, $-14 \le l \le 14$
Refractions collected	24259	7611
Independent reflections	4088	4197
No. of parameters	291	337
R_1[a]	0.029	0.0291
wR_2[a]	0.066	0.082
Goodness-of-fit	1.03	1.037
Flack parameter		0.02(4)

[a] $R_1 = \Sigma || F_o | - | F_c || \Sigma | F_o |$, $wR_2 = [\Sigma_W(|F_o| - |F_c|)^2/\Sigma w F_o^2]^{1/2}$, in which $w = 4F_o^2/\sigma^2(F_o^2)$.

Table S3 Crystallographic data of **3a**·ClO_4, **5d** and **6d**.

	**3a**·ClO_4·MeCN	**5d**·MeCN	**6d**
Empirical formula	$C_{22}H_{29}Cl_3N_5O_4Ru$	$C_{32}H_{37}Cl_2N_7O_8Ru$	$C_{32}H_{38}Cl_2N_6O_8Ru$
Formula weight	634.92	819.65	806.65
Temperature/K	100	100	100
Crystal system	Orthorhombic	Triclinic	Monoclinic
Space group	$P2_12_12_1$	P-1	$P2_1$
a (Å)	8.6233(10)	9.9120(7)	8.1503(6)
b (Å)	12.5838(16)	17.5103(14)	16.4241(13)
c (Å)	23.552(4)	21.2883(17)	12.8050(11)
α (°)	90	81.245(5)	90
β (°)	90	80.941(6)	99.016(3)
γ (°)	90	83.022(6)	90
V (Å^3)	2555.8(6)	3588.2(5)	1692.9(2)
Z	4	4	2
D_c (g cm^{-3})	1.650	1.517	1.582
μ (mm^{-1})	8.185	5.409	5.712
F(000)	1292.0	1680.0	828.0
Crystal size (mm^3)	0.3 × 0.05 × 0.04	0.2 × 0.05 × 0.01	0.25 × 0.12 × 0.02
Radiation	CuKα (λ = 1.54178)	CuKα (λ = 1.54178)	CuKα (λ = 1.54178)
2Θ range for data collection/°	7.506 to 135.434	4.242 to 101.422	6.99 to 135.37
Index ranges	-10 ≤ h ≤ 9, -14 ≤ k ≤ 14, -27 ≤ l ≤ 28	-7 ≤ h ≤ 9, -17 ≤ k ≤ 16, -21 ≤ l ≤ 21	-9 ≤ h ≤ 9, -19 ≤ k ≤ 19, -15 ≤ l ≤ 15
Reflections collected	36238	15663	27030
Independent reflections	4555 [R_{int} = 0.0491, R_{sigma} = 0.0278]	7361 [R_{int} = 0.0653, R_{sigma} = 0.0923]	5959 [R_{int} = 0.0493, R_{sigma} = 0.0382]
No. of parameters	317	950	458
Goodness-of-fit	1.068	1.031	1.055
Final *R* indexes (I>=2σ (I))	R_1 = 0.0188, wR_2 = 0.0483	R_1 = 0.0684, wR_2 = 0.1769	R_1 = 0.0273, wR_2 = 0.0688
Final *R* indexes [all data]	R_1 = 0.0188, wR_2 = 0.0485	R_1 = 0.0967, wR_2 = 0.1946	R_1 = 0.0274, wR_2 = 0.0689
Flack parameter	0.051(3)		0.028(5)

Table S4 Oxidation of racemic 2-phenylhexane with CAN catalyzed by *cis*-$[(N_4)Ru^{II}(OH_2)_2]^{2+}$ complexes.[a]

Entry	Catalyst	Product TON	Product *ee*
1	**1b**	9	1%
2	**3c·OTs**	14	5%
3	**4c·OTs**	trace	1%
4	**5c·OTs**	10	2%
5	**6c·OTs**	trace	0%

[a] Substrate (0.5 mmol), catalyst (0.005 mmol), CAN (0.75 mmol), ^{t}BuOH/H_2O (1:1 v/v, 4 mL), 0 °C, 30 min.

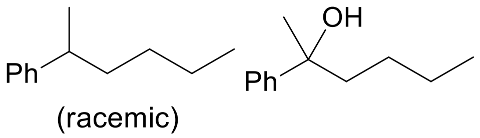

Oxidation of racemic 2-phenylhexane by "(*R*,*R*)-**3c** + CAN" was conducted at 0 °C and compared with other ruthenium catalysts (**1b**, **4c**–**6c**). Complex **3c** gave the best result which afforded the tertiary alcohol product in quantitative yield based on 14% conversion (TON = 14). However, none of the catalysts gave product with significant enantiomeric excess (*ee* <5%).

Table S5 Catalytic alkene oxidations by *cis*-[(mcp)Ru^{III}(O_2CCF_3)$_2$]ClO_4 (**1b**) in aqueous *tert*-butanol with $NaIO_4$.[a]

Entry	Substrates	Conversion (%)	C=O Products	Yield (%)[b]	Epoxidation products	Yield (%)[b]
1	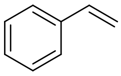	100	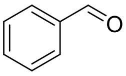	89[c]		
2	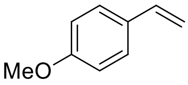	83	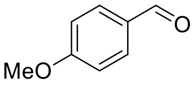	98		
3	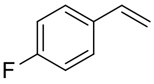	100	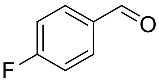	97		
4	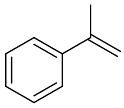	96	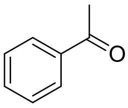	99		
5	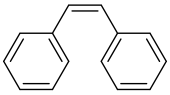	84	2 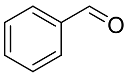	90		
6	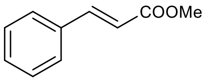	88	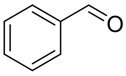	100		
7	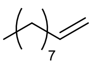	77	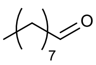	0	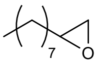	83
8	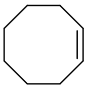	100	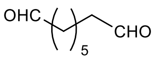	0	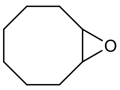	95

[a] Reaction conditions: To a mixture of *tert*-butanol (2 mL) and distilled water (1 mL) containing substrate (0.5 mmol) was added catalyst **1b** (0.005 mmol, 1 mol%). $NaIO_4$ (1.1 mmol, 2.2 equiv.) was added to the reaction mixture all at once. The reaction mixture was reacted at room temperature for 1 h. [b] Yield based on conversion. [c] When 2.2 mmol of $NaIO_4$ (4.4 equiv.) was used, the product yield of benzaldehyde dropped to 43%; with concomitant formation of benzoic acid in 55% yield.

Table S6 Catalytic alcohol oxidations by *cis*-[(mcp)$Ru^{III}(O_2CCF_3)_2]ClO_4$ (**1b**) in aqueous *tert*-butanol with H_2O_2.[a]

Entry	Substrates	Conversion (%)	Product(s)[b]	Yield (%)	Entry	Substrates	Conversion (%)	Product(s)[b]	Yield (%)
1[c]	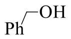	20	PhCHO	78	9	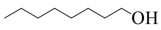	100	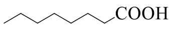	95
2	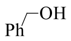	100	$PhCO_2H$<br>PhCHO	91<br>5	10	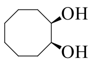	100	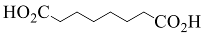	80
3	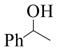	100	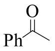	85	11	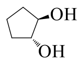	100	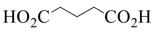	90
4	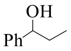	100	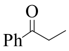	88	12	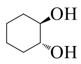	100	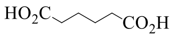	87
5	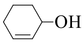	100	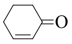	83	13	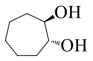	100	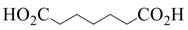	91
6	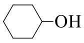	100	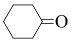	98	14	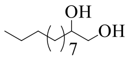	100	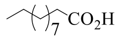	65
7	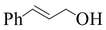	100	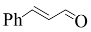<br>PhCHO	57<br>32	15	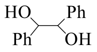	100	PhCHO<br>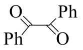	80<br>15
8	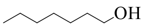	100	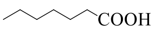	85					

[a] Reaction conditions: To a mixture of *tert*-butanol (2 mL) and distilled water (1 mL) containing substrate (1 mmol) was added catalyst **1b** (0.01 mmol, 1 mol%). Aqueous H_2O_2 (2.2 equiv. for entries 1–9; 4 equiv. for entries 10–15) was added to the refluxing reaction mixture over 8 h. The reaction mixture was further refluxed for 4 h. [b] Aldehydes / ketones were identified and quantified by GC; while carboxylic acids were obtained by column chromatography and characterized by ^{1}H NMR spectroscopy. [c] H_2O_2 was added all at once instead of added dropwise.

Catalytic alcohol oxidations by *cis*-[$Ru^{III}(mcp)(O_2CCF_3)_2]ClO_4$ (1b) with H_2O_2

Previously, we showed that [$Ru^{III}(Me_3tacn)(O_2CCF_3)_2(OH_2)]CF_3CO_2$ is an effective catalyst for the oxidation of alcohols using *tert*-butylhydroperoxide or hydrogen peroxide as terminal oxidant.[13,14] Prompted by these findings and the reactivity of **1e**, we have examined the catalytic activities of **1b** for oxidation of alcohols using H_2O_2 as a terminal oxidant. When 35% aqueous H_2O_2 (0.22 mL, 2.2 mmol) was added dropwise *via* syringe pump to a mixture of benzyl alcohol (1 mmol) and **1b** (10 µmol; 1 mol%) in refluxing aqueous *tert*-butanol, benzoic acid (91%) and benzaldehyde (5%) were formed with 100% substrate conversion (Table S6, entry 2). However, when the reaction was conducted without dropwise addition of H_2O_2, the substrate conversion was markedly reduced to only 20% with benzaldehyde obtained in 78% yield (based on conversion, Table S6, entry 1).

Secondary alcohols such as 1-phenylethanol and 1-phenyl-1-propanol were converted to the corresponding ketones in 85% and 88% yields with complete substrate consumption (Table S6, entries 3 and 4). Likewise, the catalytic oxidation of 2-cyclohexen-1-ol and cyclohexanol produced 2-cyclohexen-1-one (83% yield) and cyclohexanone (98% yield), respectively (Table S6, entries 5 and 6). Oxidation of *trans*-cinnamyl alcohol produced 57% yield of cinnamyl aldehyde along with 32% yield of benzaldehyde, presumably *via* C=C bond cleavage reaction (Table S6, entry 7). Terminal alcohols such as 1-heptanol and 1-octanol were effectively oxidized to the corresponding carboxylic acids in 85–95% yields (Table S6, entries 8 and 9). When *cis*-cyclooctane-1,2-diol was treated with aqueous H_2O_2 (4 equiv.) and **1b** (1 mol%) in refluxing aqueous *tert*-butanol solution for 12 h, octane-1,8-dicarboxylic acid was obtained in 80% yield (Table S6, entry 10). Other 1,2-diols such as *trans*-cyclopentane-1,2-diol, *trans*-cyclohexane-1,2-diol, *trans*-cycloheptane-1,2-diol and docecane-1,2-diol also underwent oxidative cleavage to form terminal carboxylic acids in 65-91% yields under the Ru-catalyzed conditions (Table S6, entries 11-14). Oxidation of 1,2-diphenylethane-1,2-diol gave predominantly benzaldehyde in 80% yield (Table S6, entry 15).

ESI-MS analysis of a mixture of **1b** (1×10^{-4} M) and H_2O_2 (10 equiv.) in water gave an intense signal attributable to $[(mcp)Ru^{III}(OH)_2]^+$. No signals assignable to $[Ru^{VI}(O)_2]$, $[Ru^{V}(O)_2]$, $[Ru^{IV}(O)]$, or $[Ru^{III}(OOH)]$ species could be detected.

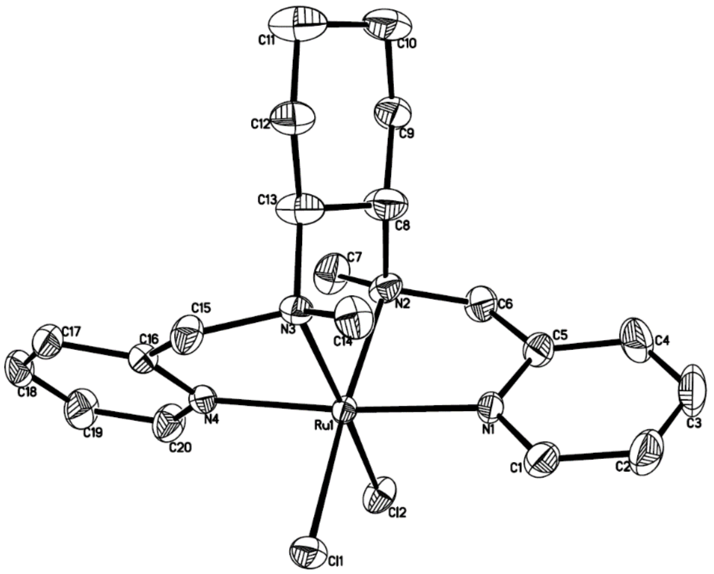

Fig. S1 ORTEP drawing of the cation of *cis*-[(mcp)$Ru^{III}Cl_2$]ClO_4 (**1a**). Thermal ellipsoids are at 50% probability level.

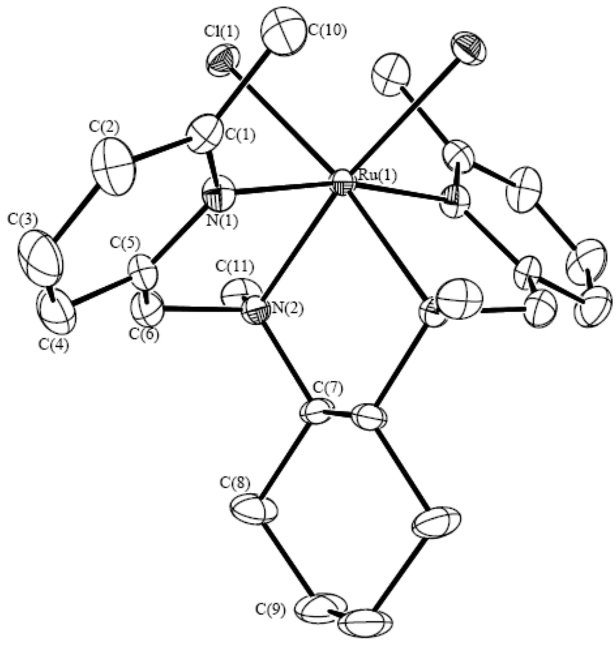

Fig. S2 ORTEP drawing of one (**I**) of the two crystallographically independent cations present in the crystals of *cis*-[(Me_2mcp)$Ru^{III}Cl_2$]ClO_4 (**2a**). Thermal ellipsoids are at 30% probability level.

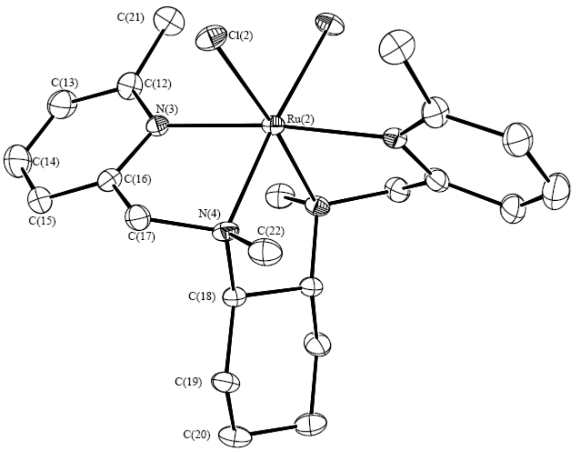

Fig. S3 ORTEP drawing of one (**II**) of the two crystallographically independent cations present in the crystals of *cis*-[(Me$_2$mcp)Ru^{III}Cl$_2$]ClO$_4$ (**2a**). Thermal ellipsoids are at 30% probability level.

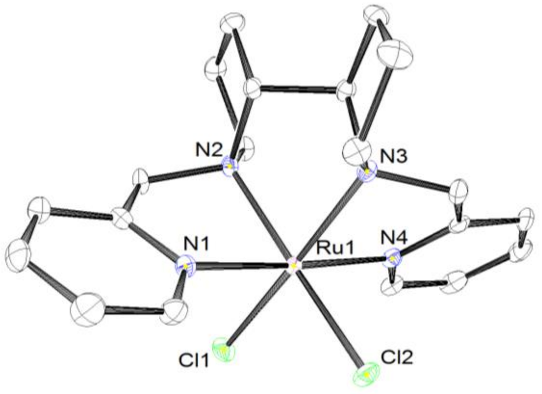

Fig. S4 ORTEP drawing of the cation of *cis*-[(pdp)Ru^{III}Cl$_2$]ClO$_4$ (**3a·ClO$_4$**). Thermal ellipsoids are at 50% probability level.

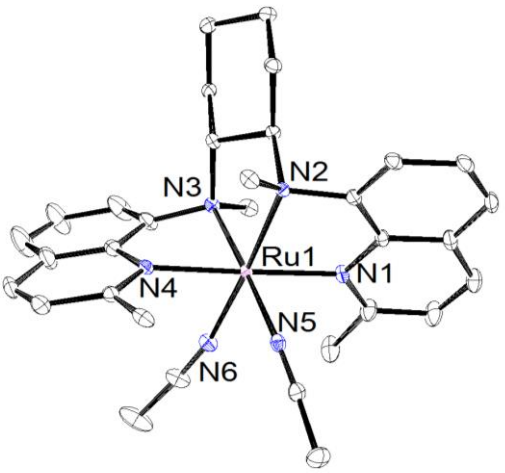

Fig. S5 ORTEP drawings of the cation of *cis*-[(Me$_2$bqcn)Ru^{II}(NCMe)$_2$](ClO$_4$)$_2$ (**6d**). Thermal ellipsoids are at 50% probability level.

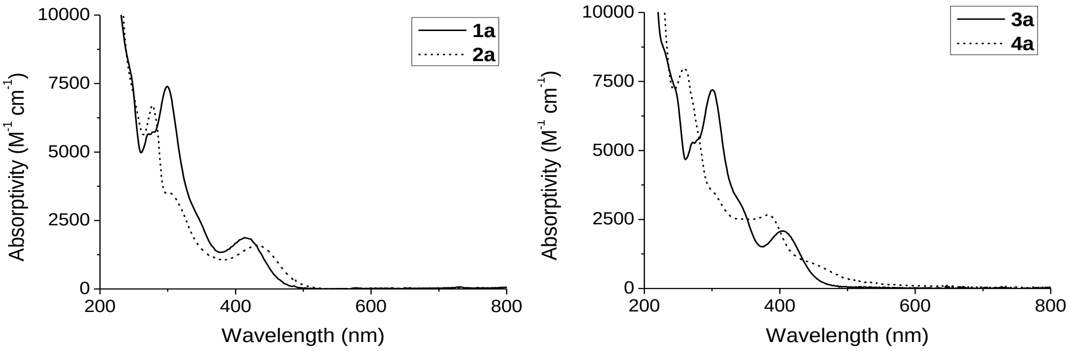

Fig. S6 UV-Vis absorption spectra of **1a** and **2a** (left) and **3a** and **4a** (right) in acetonitrile.

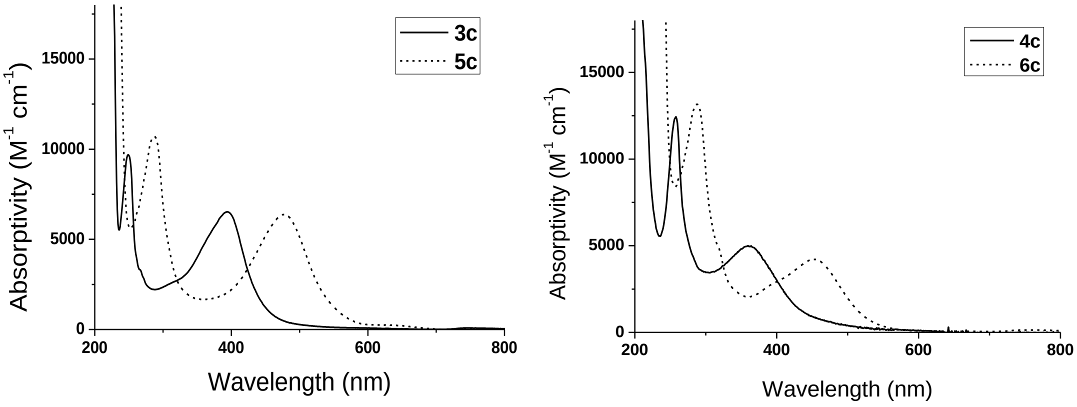

Fig. S7 UV-Vis absorption spectra of **3c** and **5c** (left) and **4c** and **6c** (right) in water.

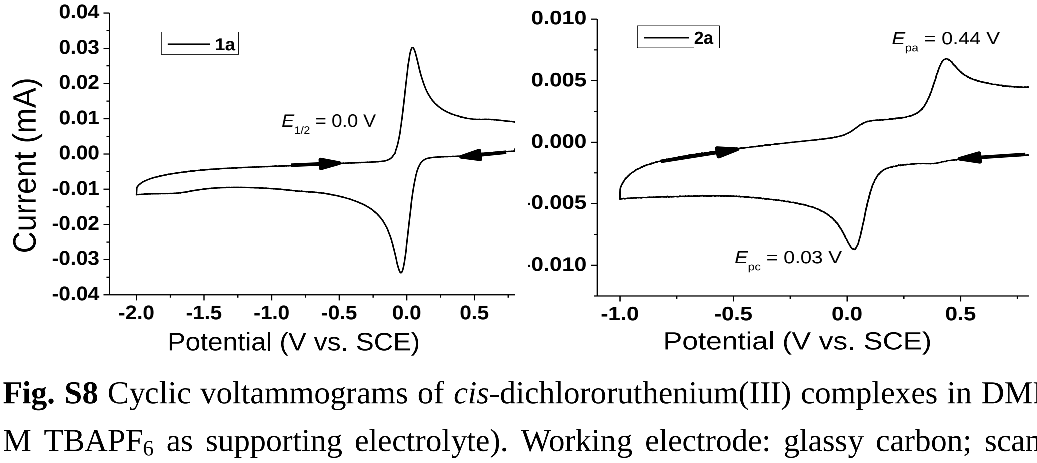

Fig. S8 Cyclic voltammograms of *cis*-dichlororuthenium(III) complexes in DMF (0.1 M $TBAPF_6$ as supporting electrolyte). Working electrode: glassy carbon; scan rate: 0.1 V s^{-1}. Initial potential: 0.8 V. Initial scan direction: reduction.

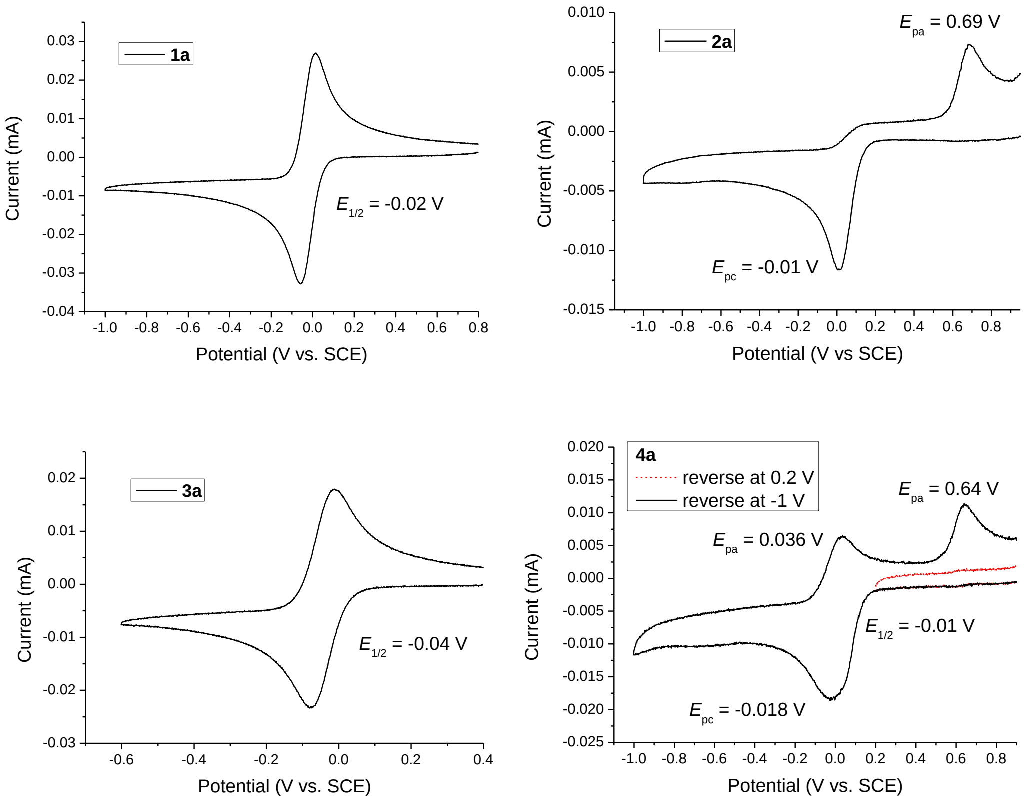

Fig. S9 Cyclic voltammograms of *cis*-dichlororuthenium(III) complexes **1a** – **4a** in acetonitrile (0.1 M $TBAPF_6$ as supporting electrolyte). Working electrode: glassy carbon; scan rate: 0.1 V s^{-1}. Initial potential: 0.8 – 1.0 V. Initial scan direction: reduction.

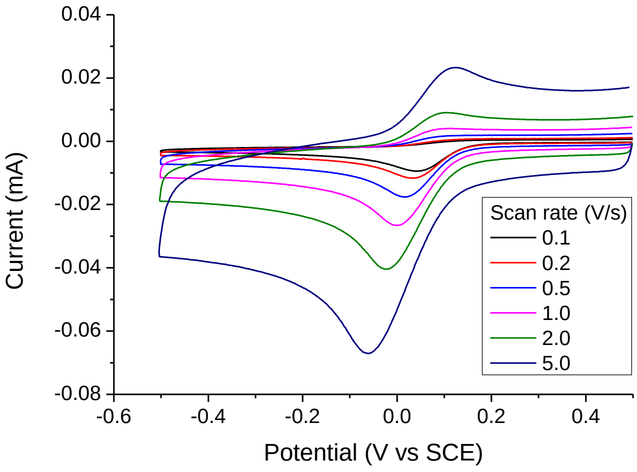

Fig. S10 Cyclic voltammograms of **2a** in acetonitrile (0.1 M $TBAPF_6$ as supporting electrolyte) at different scan rates (0.1–5.0 V s^{-1}).

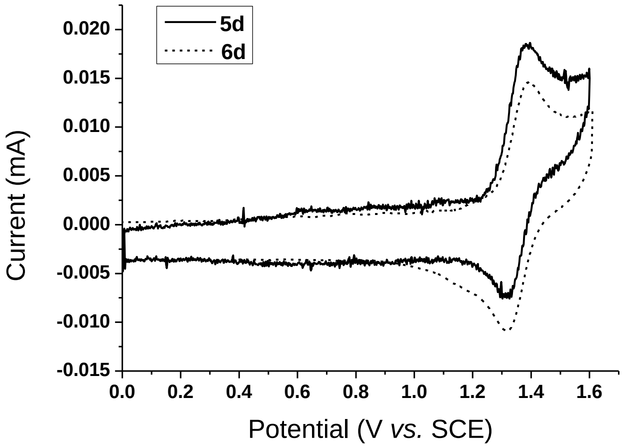

Fig. S11 Cyclic voltammograms of **5d** and **6d** in acetonitrile (0.1 M $TBAPF_6$ as supporting electrolyte). Working electrode: glassy carbon; scan rate: 0.1 V s^{-1}.

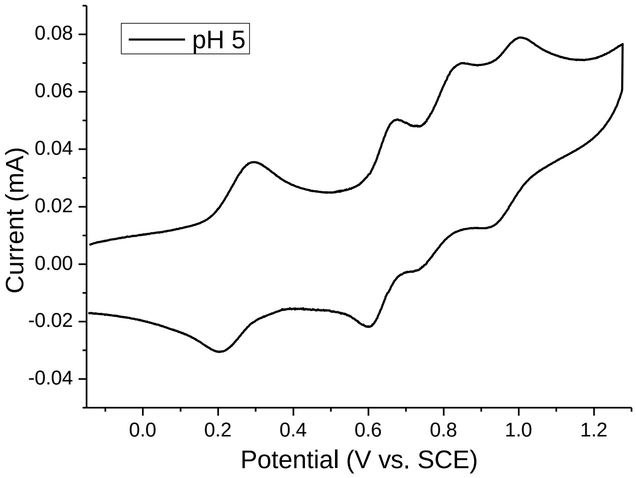

Fig. S12 Cyclic voltammogram of *cis*-[(mcp)$Ru^{III}(O_2CCF_3)_2$]ClO_4 (**1b**) at pH 5 (acetate buffer). Working electrode: edge-plane pyrolytic graphite.

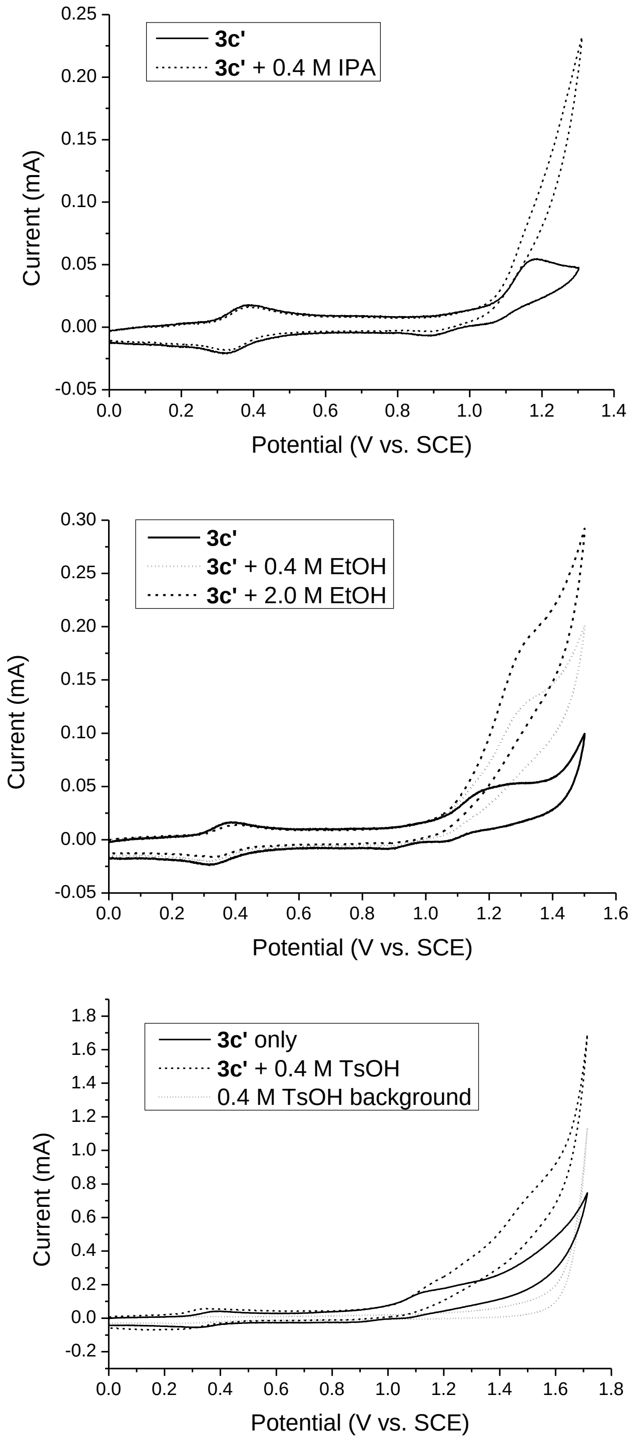

Fig. S13 Cyclic voltammograms of **3c′** in 0.1 M CF_3SO_3H (pH 1) at 0.1 V s^{-1} in the absence or presence of propan-2-ol (IPA; top), ethanol (EtOH; middle) and tosylic acid (TsOH; bottom). Working electrode: edge-plane pyrolytic graphite.

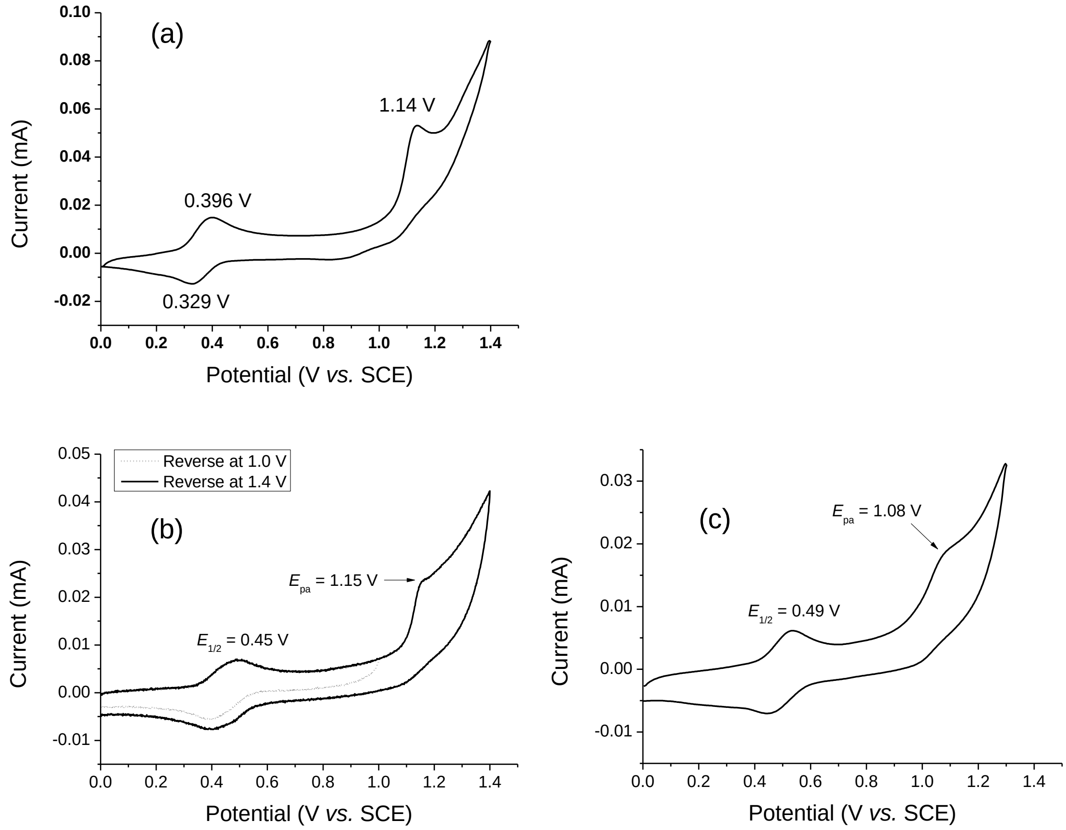

Fig. S14 Cyclic voltammograms in 0.1 M CF_3SO_3H (pH 1) of (a) *cis*-[(pdp)$Ru^{II}(OH_2)_2$](OTs)$_2$ (**3c·OTs**), (b) *cis*-[(bqcn)$Ru^{II}(OH_2)_2$](OTs)$_2$ (**5c·OTs**) and (c) *cis*-[(Me_2bqcn)$Ru^{II}(OH_2)_2$](OTs)$_2$ (**6c·OTs**); scan rate: 0.1 V s^{-1}.

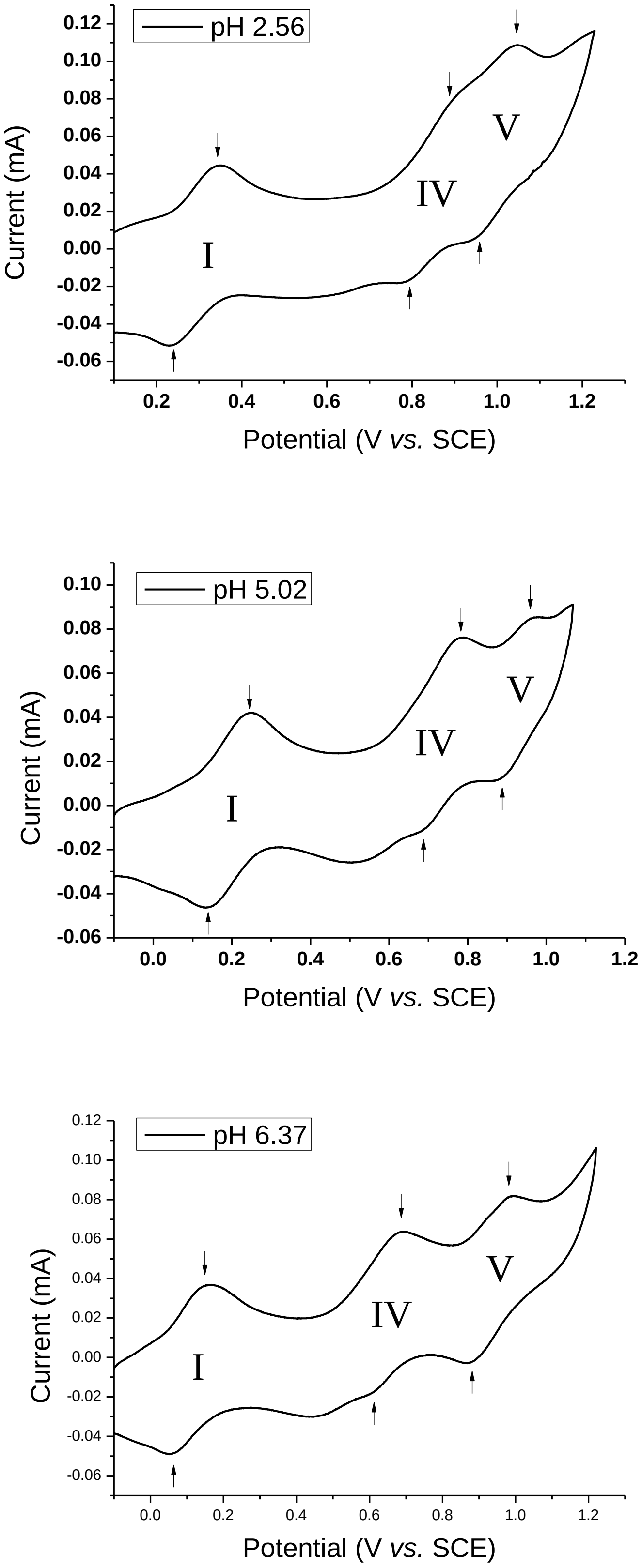

Fig. S15 Cyclic voltammograms at 0.1 V s^{-1} of **3c′** in Britton-Robinson buffer at pH 2.56 (top), pH 5.02 (middle) and pH 6.37 (bottom).

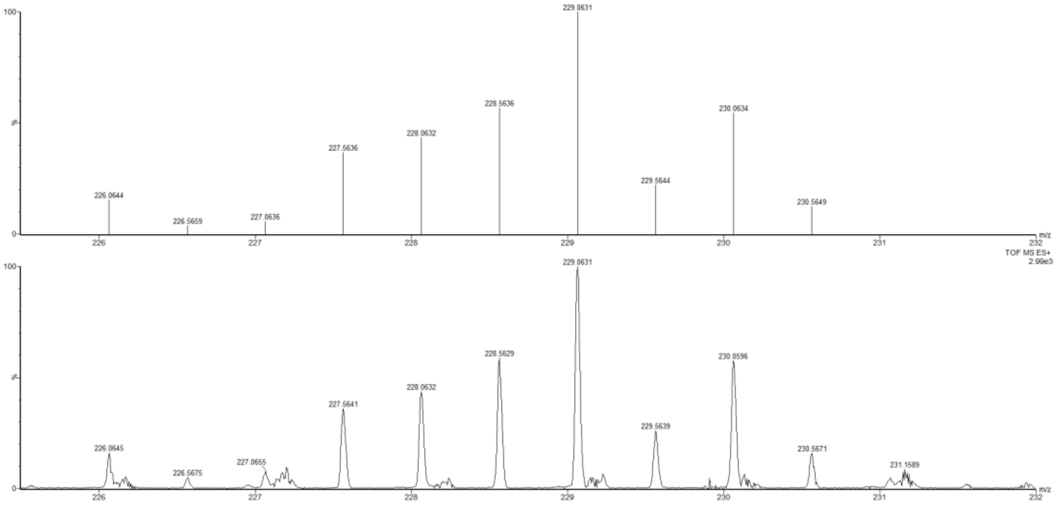

Fig. S16 (Top) Simulation of $[(mcp)Ru^{VI}(O)_2]^{2+}$. (Bottom) Experimental ESI-MS signals of **1e** in water.

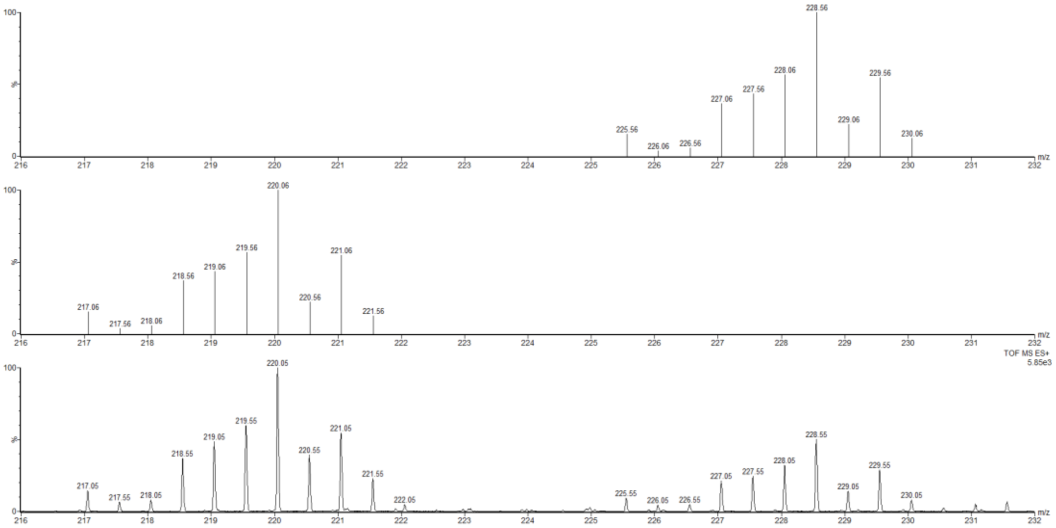

Fig. S17 (Top) Simulation of $[(pdp)Ru^{V}(O)(OH)]^{2+}$. (Middle) Simulation of $[(pdp)Ru^{IV}(O)]^{2+}$. (Bottom) Experimental ESI-MS signals for a reaction mixture of **3c·CF$_3$SO$_3$** and 4 equiv. of $Ce^{IV}(ClO_4)_4$, [Ru] = 1 × 10^{-4} M.

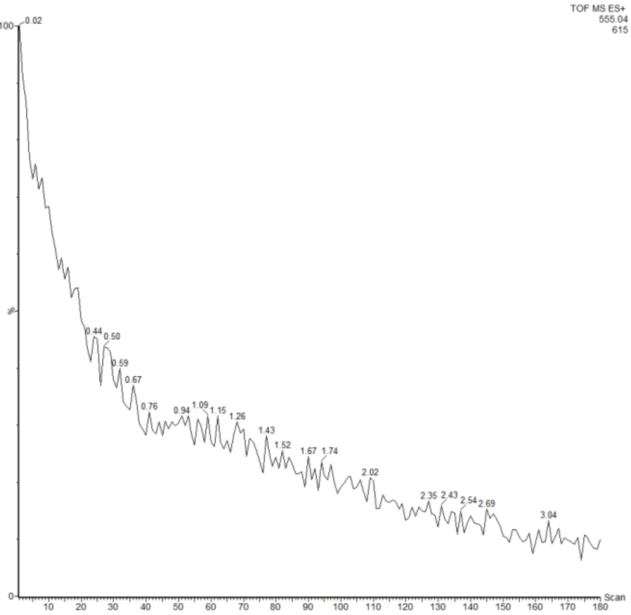

Fig. S18 Time trace from 0 to 3 min of the signal intensity of *m/z* = 555.05 ion.

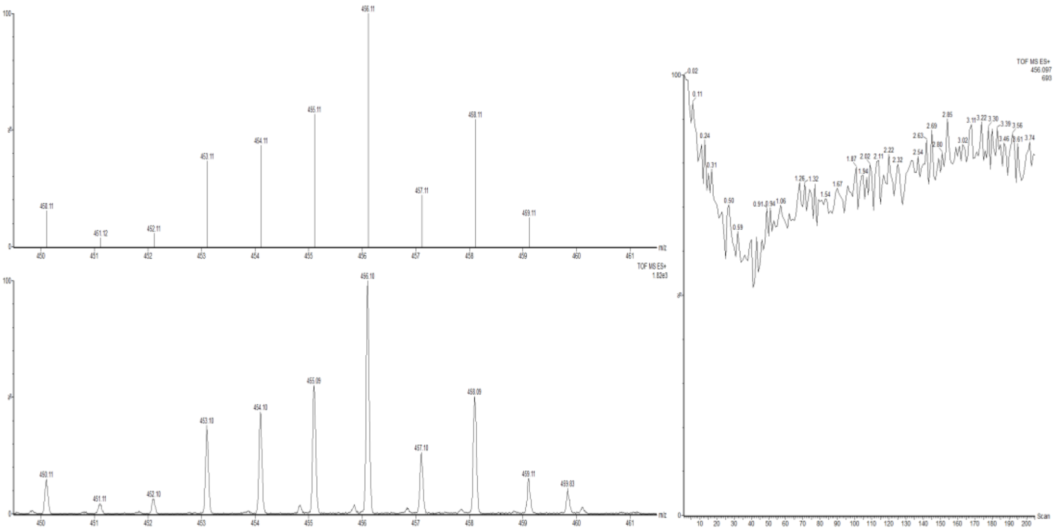

Fig. S19 (a) (Top-left) Simulation of $[(pdp)Ru^{V}(O)_2]^+$. (Bottom-left) Experimental ESI-MS signals for a reaction mixture of **3c·CF$_3$SO$_3$** and 6 equiv. of $Ce^{IV}(ClO_4)_4$, [Ru] = 1 × 10^{-4} M. **(b)** (Right) Time trace from 0 to 3 min of the signal intensity of *m/z* = 456.10 ion.

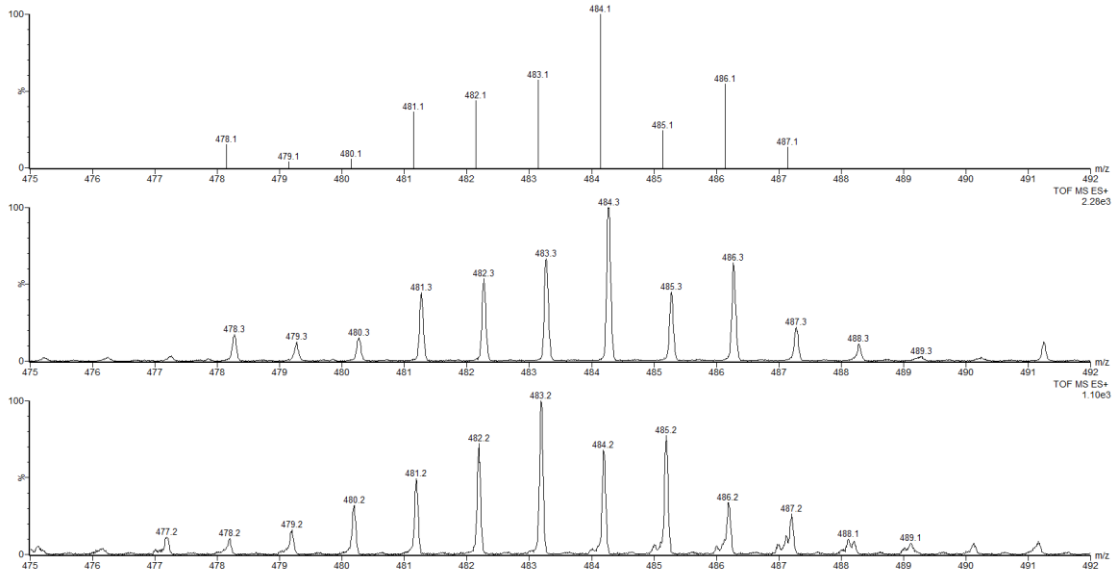

Fig. S20. (Top) Simulation of $[(Me_2pdp)Ru^{V}(O)_2]^{+}$. (Middle) Experimental ESI-MS signals for a reaction mixture of **4c·OTs** and 6 equiv. of CAN, [Ru] = 1 × 10^{-4} M. (Bottom) Experimental ESI-MS signals for a reaction mixture of **4c·OTs** and 10 equiv. of CAN, [Ru] = 1 × 10^{-4} M.

When an aqueous solution of *cis*-$[(Me_2pdp)Ru^{II}(OH_2)_2](OTs)_2$ (**4c·OTs)** was treated with CAN (6 equiv.), a new signal was detected at *m/z* 484.3 assignable to $[(Me_2pdp)Ru^{V}(O)_2]^{+}$ (see middle of Fig. S20). Subsequent increase of the CAN concentration to 10 equiv. did not produce signals attributed to Ru(VI) species. Instead, a shift of the *m/z* 484.3 signal to *m/z* 483.2 was observed (see bottom of Fig. S20). We propose that intramolecular oxidation of the *ortho*-methyl group has occurred via a highly reactive *cis*-dioxoruthenium(VI) intermediate:

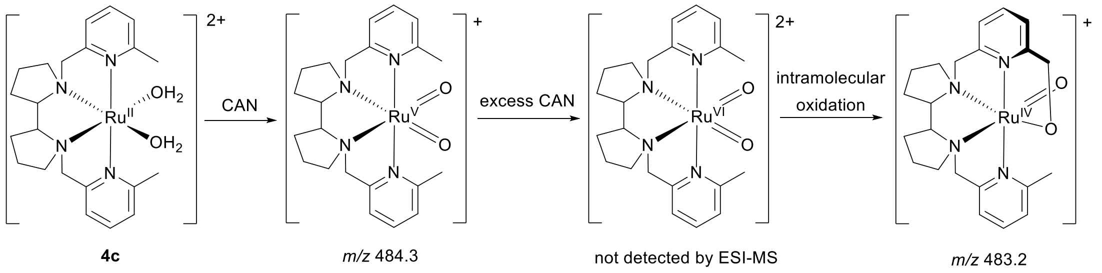

Similar oxygenation of aliphatic C–H bond adjacent to pyridine ring has been documented.[15]

NMR spectra of oxidation products P11–P14

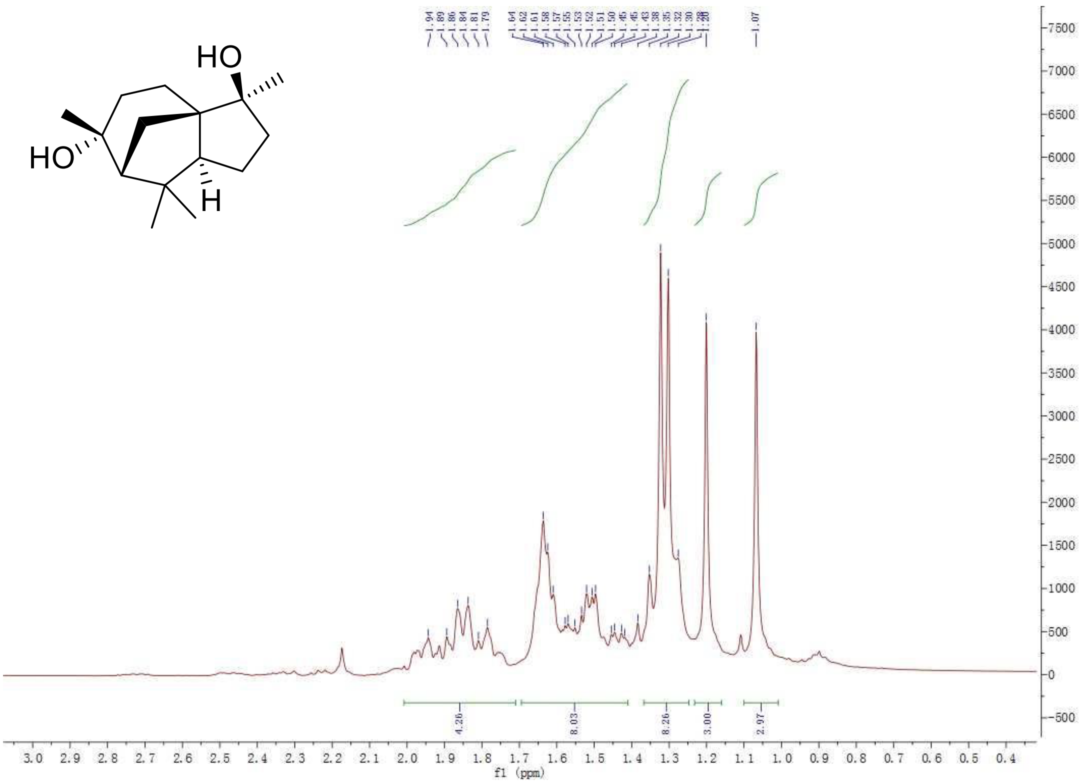

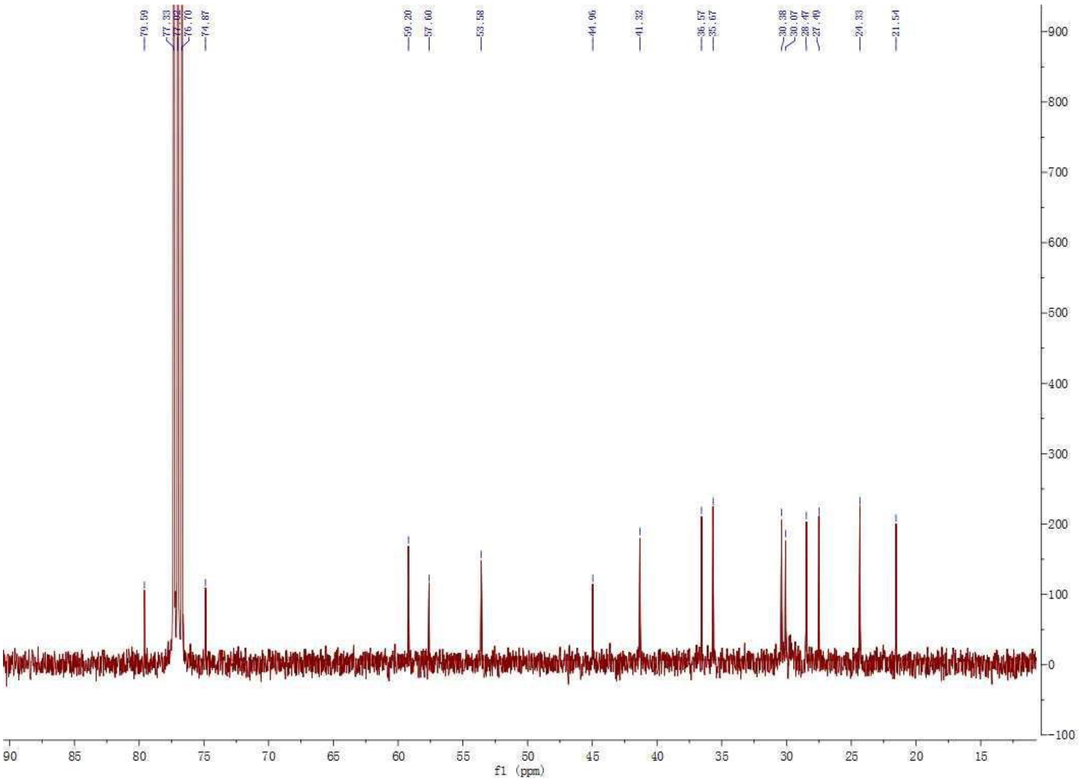

P11: Matches with reported NMR data (ref. 16).

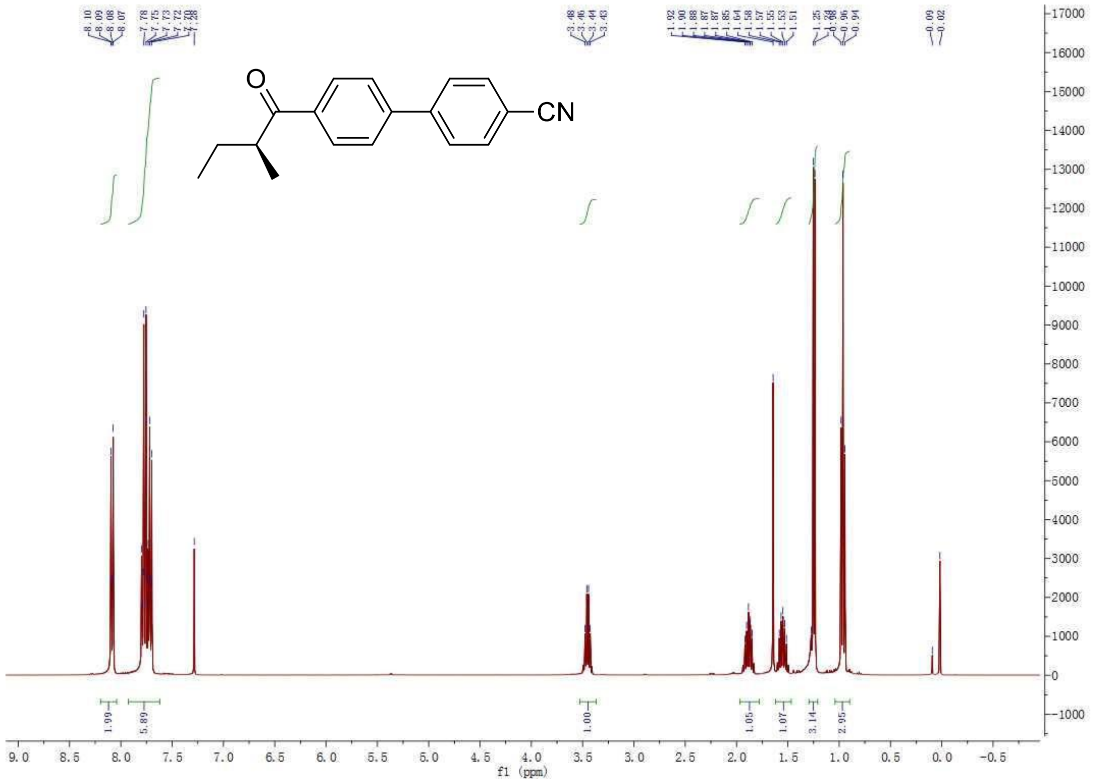

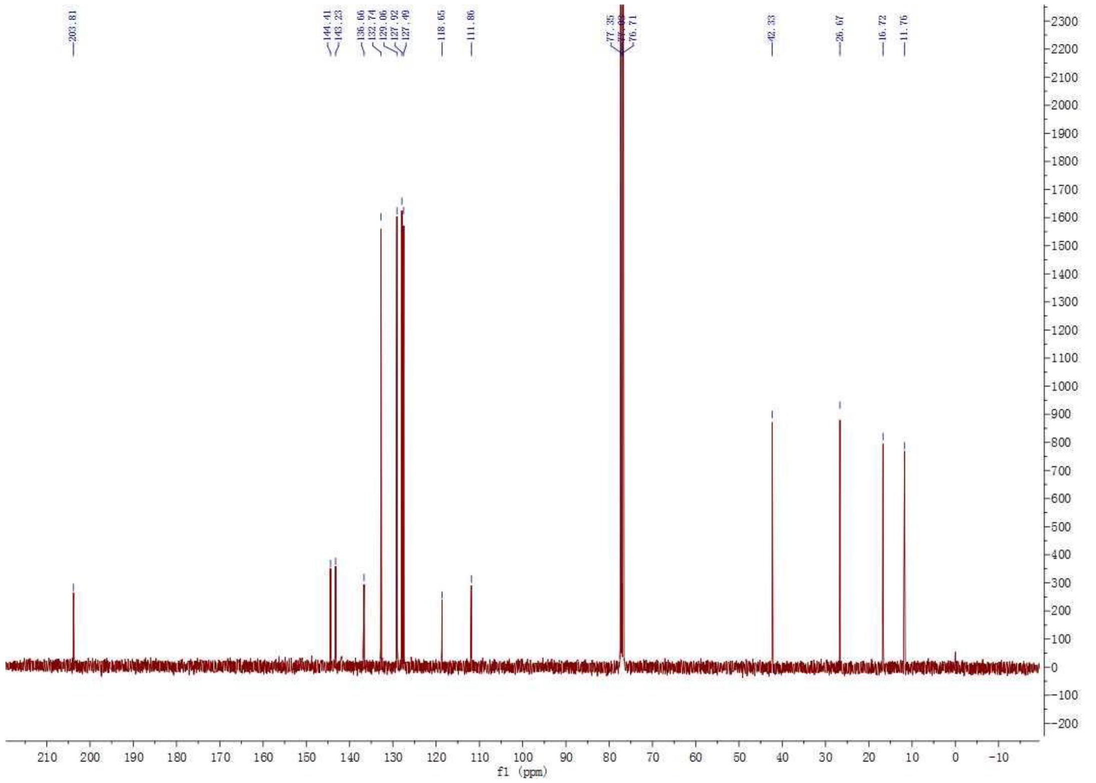

P12: ^{1}H NMR (400 MHz, $CDCl_3$): δ 8.07–8.10 (d, 2H), 7.70–7.78 (m, 6H), 3.43–3.48 (m, 1H), 1.85–1.92 (m, 1H), 1.51–1.58 (m, 1H), 1.24–1.25 (d, 3H), 0.94–0.98 (t, 3H). ^{13}C NMR (100 MHz, $CDCl_3$): δ 203.8, 144.4, 143.2, 136.6, 132.7, 129.1, 127.9, 127.5, 118.7, 111.9, 42.3, 26.7, 16.7, 11.8.

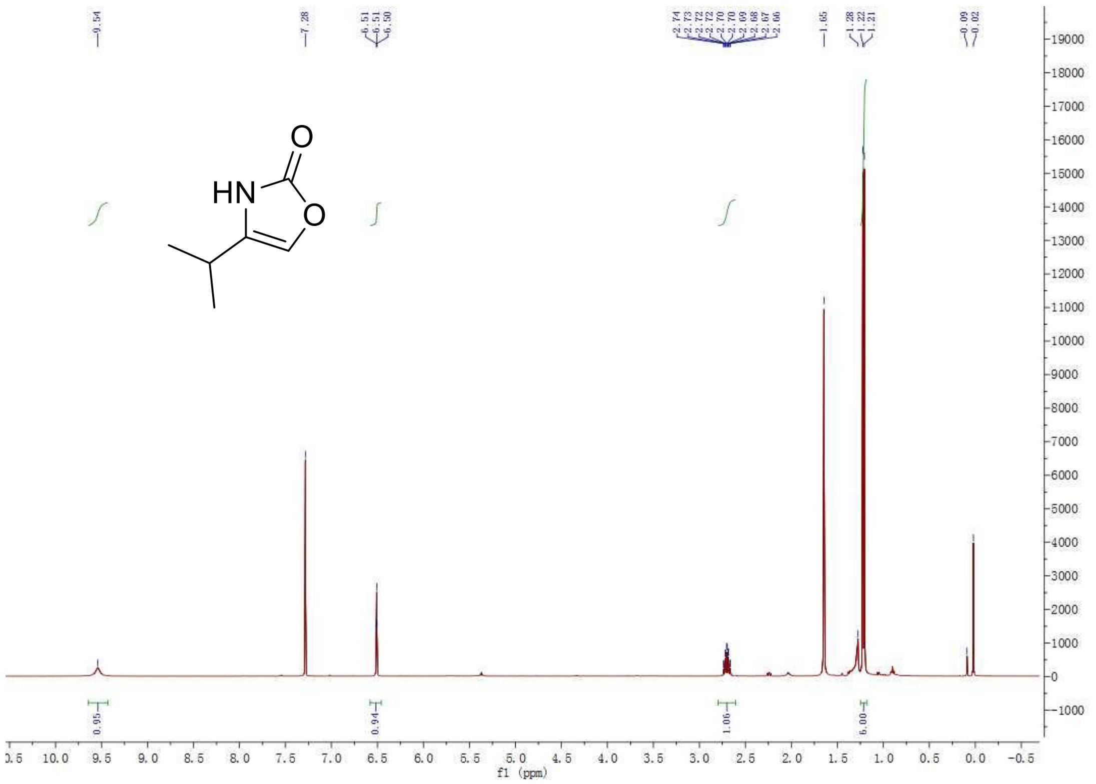

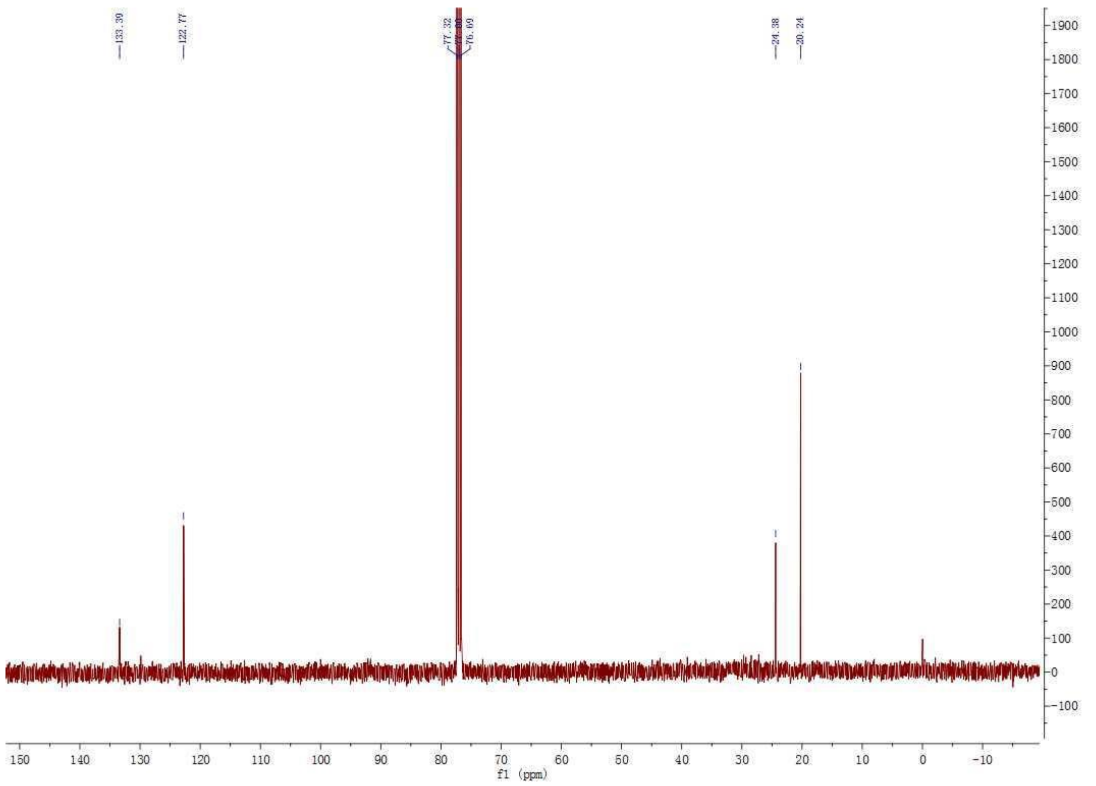

P13: ^{1}H NMR (400 MHz, $CDCl_3$): δ 9.54 (s, 1H), 6.51 (s, 1H), 2.66–2.74 (m, 1H), 1.21–1.28 (d, 6 H). ^{13}C NMR (100 MHz, $CDCl_3$): 158.2, 133.4, 122.8, 24.4, 20.2. MS (EI): 127.0

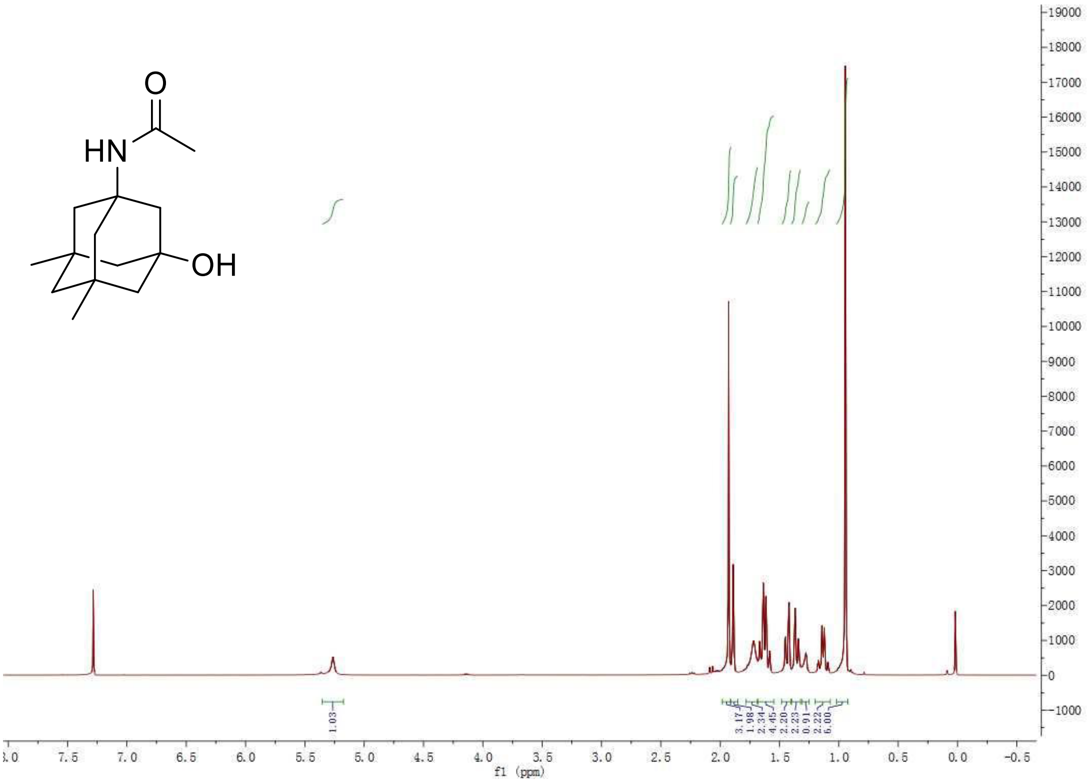

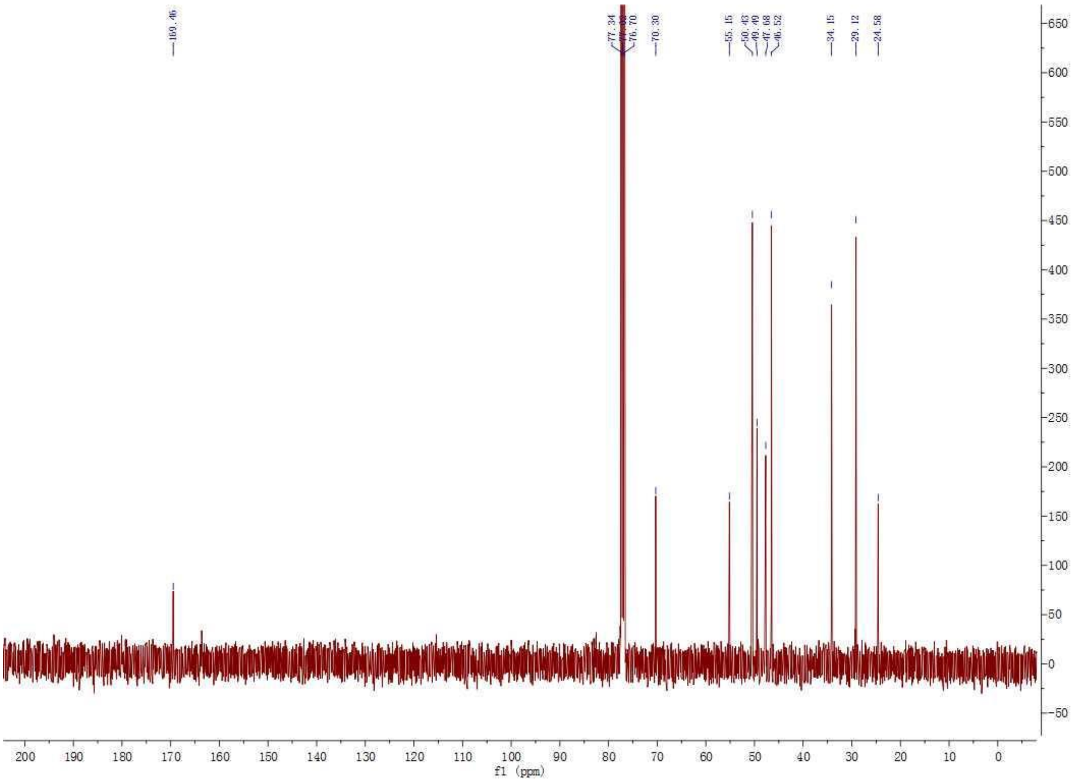

P14: ^{1}H NMR (400 MHz, $CDCl_3$): δ 5.26 (s, 1H), 1.93 (s, 3H), 1.89 (s, 2H), 1.58–1.64 (m, 4H), 1.29–1.45 (m, 5H), 1.09–1.18 (m, 2H), 0.94 (s, 6H). ^{13}C NMR (100 MHz, $CDCl_3$): 169.5, 70.3, 55.2, 50.4, 49.5, 47.7, 46.5, 34.2, 29.2, 24.6.

Notes and references

1 W. L. F. Armarego and C. L. L. Chai, *Purification of Laboratory Chemicals*, 6th edition; Elsevier Inc, 2009.

2 A. Murphy, G. Dubois and T. D. P. Stack, *J. Am. Chem. Soc.*, 2003, **125**, 5250–5251.

3 D. H. Jo, Y. M. Chiou and L. Que, Jr. *Inorg. Chem.*, 2001, **40**, 3181.

4 M. S. Chen and M. C. White, *Science*, 2007, **318**, 783.

5 K. Suzuki, P. D. Oldenburg and L. Que, Jr., *Angew. Chem., Int. Ed.*, 2008, **47**, 1887.

6 J. England, G. J. P. Britovsek, N. Rabadia and A. J. P. White, *Inorg. Chem.*, 2007, **46**, 3752.

7 C. Zang, Y. Liu, Z.-J. Xu, C.-W. Tse, X. Guan, J. Wei, J.-S. Huang and C.-M. Che, *Angew. Chem., Int. Ed.* 2016, **55**, 10253.

8 P. Bernhard, M. Biner and A. Ludi, *Polyhedron*, 1990, **9**, 1095.

9 C. Fellay and G. Laurenczy, *Inorg. Synth.*, 2010, **35**, 2010.

10 S. Guo, X. Zhang and P. Tang, *Angew. Chem. Int. Ed.*, 2015, **54**, 4065.

11 E. McNeill and J. Du Bois, *Chem. Sci.*, 2012, **3**, 1810.

12 The signals arising from tosylate anion (OTs^-) are omitted for clarity, which were present at δ 2.30 (s, 6H), 7.14–7.15 (d, 4H, *J* = 7.6 Hz), 7.58–7.60 (d, 4H, *J* = 7.6 Hz).

13 W.-C. Cheng, W.-H. Fung and C.-M. Che, *J. Mol. Catal. A - Chem.*, 1996, **113**, 311.

14 C.-M. Che, W.-P. Yip and W.-Y. Yu, *Chem. Asian. J.*, 2006, **1**, 453.

15 C.-M. Che, V. W.-W. Yam and T. C. W. Mak, *J. Am. Chem. Soc.*, 1990, **112**, 2284.

16 G. Maatooq, S. EL-Sharkawy, M. S. Afifi and J. P. N. Rosazza, *J. Nat. Prod.*, 1993, 1039.
